# Network Pharmacology of Red Ginseng (Part I): Effects of Ginsenoside Rg5 at Physiological and Sub-Physiological Concentrations

**DOI:** 10.3390/ph14100999

**Published:** 2021-09-29

**Authors:** Alexander Panossian, Sara Abdelfatah, Thomas Efferth

**Affiliations:** 1 EuroPharma USA Inc., Green Bay, WI 54311, USA; 2 Department of Pharmaceutical Biology, Institute of Pharmaceutical and Biomedical Sciences, Johannes Gutenberg University, 55131 Mainz, Germany; saabdelf@uni-mainz.de

**Keywords:** red ginseng, ginsenoside Rg5, gene expression, IPA pathways, network pharmacology, transcriptomics

## Abstract

Numerous in vitro studies on isolated cells have been conducted to uncover the molecular mechanisms of action of *Panax ginseng* Meyer root extracts and purified ginsenosides. However, the concentrations of ginsenosides and the extracts used in these studies were much higher than those detected in pharmacokinetic studies in humans and animals orally administered with ginseng preparations at therapeutic doses. Our study aimed to assess: (a) the effects of ginsenoside Rg5, the major “rare” ginsenoside of Red Ginseng, on gene expression in the murine neuronal cell line HT22 in a wide range of concentrations, from 10^−4^ to 10^−18^ M, and (b) the effects of differentially expressed genes on cellular and physiological functions in organismal disorders and diseases. Gene expression profiling was performed by transcriptome-wide mRNA microarray analyses in HT22 cells after treatment with ginsenoside Rg5. Ginsenoside Rg5 exhibits soft-acting effects on gene expression of neuronal cells in a wide range of physiological concentrations and strong reversal impact at high (toxic) concentration: significant up- or downregulation of expression of about 300 genes at concentrations from 10^−6^ M to 10^−18^ M, and dramatically increased both the number of differentially expressed target genes (up to 1670) and the extent of their expression (fold changes compared to unexposed cells) at a toxic concentration of 10^−4^ M. Network pharmacology analyses of genes’ expression profiles using ingenuity pathway analysis (IPA) software showed that at low physiological concentrations, ginsenoside Rg5 has the potential to activate the biosynthesis of cholesterol and to exhibit predictable effects in senescence, neuroinflammation, apoptosis, and immune response, suggesting soft-acting, beneficial effects on organismal death, movement disorders, and cancer.

## 1. Introduction

The *Panax ginseng* Meyer root has been traditionally used in China, Korea, and Japan for thousands of years for many conditions, including the age-related decline of cognitive function, general weakness, and enhancing longevity [1,2,3,4,5,6,7]. In Europe, ginseng preparations are recognized as a general tonic or adaptogen in cases of fatigue, weakness, and decreased mental and physical capacity at daily doses equivalent to 600–2000 mg of powdered herbal substance [3,4]. The results of many clinical trials suggesting the beneficial effects of ginseng on stress and cognitive functions were critically reviewed in several comprehensive and systematic review articles [3,8,9,10,11,12,13,14]. Overall, ginseng is a promising treatment for mental, industrial, and chronic fatigue [15,16,17,18,19], and for the cognitive enhancement performance of healthy subjects [6,19,20,21,22,23,24], and patients with mild cognitive impairments [25,26,27,28,29,30,31] and/or neurological disorders [7,26,27,28].

Numerous in vitro studies on isolated cells have been conducted to uncover the molecular mechanisms of action of ginseng extracts and isolated ginsenosides (Supplemental Table S1 in Supplement 1). However, the concentrations of ginsenosides and the extracts used in these studies [32,33,34,35,36,37,38,39,40,41,42,43] were incompatibly higher than those detected in pharmacokinetic (PK) studies in humans [44,45] and animals orally administrated with ginseng preparations at the highest therapeutic doses [46,47,48,49,50,51] (Supplemental Table S2 in Supplement 1).

As an example, ginsenoside Rg5, the major ginsenoside of Red Ginseng, was tested in vitro (Supplemental Table S2 in Supplement 1) mainly in the concentration range from 20 to 100 μM, while the maximal concentration of Rg5 was 2–10 nM (about 2000-fold lower) in the blood of human subjects taking ginseng orally at a dose of 9 g [44] (Supplemental Table S2 in Supplement 1). The maximal concentration of Rg5 was only 81 nM [48] (20-3300-fold lower) in the blood of dogs after orally administered ginseng at a dose of 5 g (corresponding human dose: 39 g). These discrepancies/inconsistencies raise the question about the clinical relevance of results obtained from in vitro models (where substantially higher concentrations were used) and the pharmacological activity of ginsenosides and their metabolites in nano-molar concentrations detected in animal blood and human subjects.

To the best of our knowledge, the only in vitro study where the pharmacological activity of ginsenosides had been tested at nano-molar concentrations was the study, where in ginsenosides Rg5, Rb1, and Rc protected neurons from glutamate-induced apoptosis in vitro in Huntington’s disease (HD) assay at concentrations of 1000, 100 and 10 nM, respectively [32]. Ginsenosides Rd, Re, Rg3, Rh1, Re, Rd, Rk1, Rh4, and Rk3 were inactive or exerted toxic effects [32]. Ginsenoside Rg5 was toxic at concentrations from 78 to 104 μM in experiments with murine hippocampal HT22 cells and exhibited a neuroprotective effect in heat stress-induced apoptosis [33]. The lower concentrations have not been studied in in vitro experiments even though ginsenosides and their metabolites were detected at nano-molar concentrations in the circulating blood system of human subjects (Supplemental Table S2 in Supplement 1). Furthermore, levels of brain hormones might not be the same as those in the blood, as it has been reported for estrogens that levels in the brain were 0.08–0.19 ng/g wet weight [52].

Our study aimed to assess: (a) the effects of ginsenoside Rg5, the major “rare” ginsenoside of Red Ginseng, on gene expression in the murine neuronal cell line HT22 in a wide range of concentrations from 10^−4^ to 10^−8^ M, and (b) the effects of differentially expressed genes on cellular and physiological functions in organismal disorders and diseases.

## 2. Results

### 2.1. Effect of Ginsenoside Rg5 on Gene Expression Profiles in the Murine Hippocampal Neuronal Cell Line HT22

**Figure 1:**
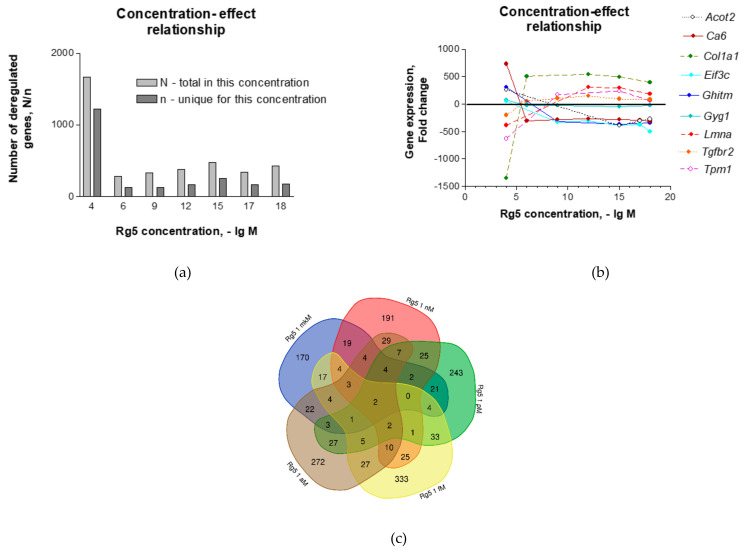
(**a**) The total number of genes deregulated by ginsenoside Rg5 in concentrations ranging from 1 μM to 1 aM; (**b**) ginsenoside Rg5 concentration-dependent fold change expression of selected differentially regulated genes in the hippocampal neuronal cell line HT22; (**c**) Venn diagram of genes deregulated by ginsenoside Rg5 at concentrations 1 μM, 1 nM, 1 pM, 1 gM and 1 aM.

Table 1 and Figure 1a show that the total number of genes deregulated by ginsenoside Rg5 in a concentration range from 1 μM to 1 aM is roughly the same—370 ± 69 (RSD = 18.53%), while at a toxic concentration of 100 μM Rg5 differentially regulated 1670 genes (~4.5-fold more). A majority (73%) of these genes (1215 of 1670) were uniquely differentially regulated by Rg5 only at this toxic concentration of 100 μM.

The effect size of gene expression was in a range from 300 to 800-fold change compared to their expression in control cells unexposed to Rg5 at all tested concentrations except the highest toxic concentration of 100 μM. In the latter case, the gene expression was increasing up to 2600-fold, particularly of genes which were not deregulated at lower concentrations (Supplemental Table S3a in Supplement 2).

At the highest (toxic) concentration, Rg5 dramatically increased the fold change of many genes in a dose-response reversal manner (Figure 1b), a common phenomenon known as hormesis. That was in line with other observations, where ginsenosides, including Rg5, exhibited hormetic response in various in vitro and in vivo studies [53].

Only nine of 2897 differentially regulated genes (Supplemental Table S3 in Supplement 2), were commonly deregulated by ginsenoside Rg5 at five or more tested concentrations (*Ca6, Cth, Col1a1, Dipk2a, Eif3c, Enox2, Gyg1, Tgfbr2* and *1700001F09Rik*), and only two genes (*Ca6* and *Tgfbr2*) at concentrations from 1 μM to 1 aM (Figure 1c, and Tables S3b–e,g in Supplement 2).

Only one gene, *Ca6* encoding extracellular enzyme carbonic anhydrase 6, was differentially regulated in all tested concentrations (Table 1), but in a reversed manner: upregulation at the highest 100 μM concentration, and downregulation at all other concentrations of Rg5.

These observations suggest that Rg5 was pharmacologically active in a wide range of concentrations of neuronal cells from 10^−6^ M to 10^−18^ M (Figure 1c) and had a significant impact on the gene expression of hippocampal neurons.

### 2.2. Effect of Ginsenoside Rg5 on Signaling Canonical Pathways

**Figure 2:**
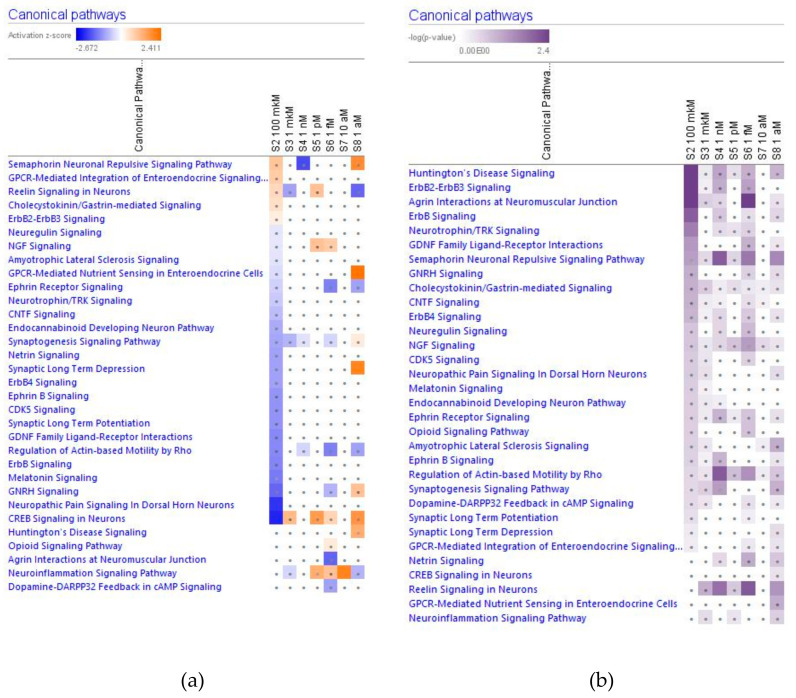
Effect of Rg5 in concentrations of 1 aM, 10 aM, 1 fM, 1 pM, 1 nM, 1 μM and 100 μM) on neurotransmitters and nervous system signaling. (**a**) Brown color depicts predicted activation, blue color—predicted inhibition of signaling pathway; symbol shows that the activation z-score was <2. (**b**) Symbol shows that the −log *p*-value was <1.3.

**Figure 3:**
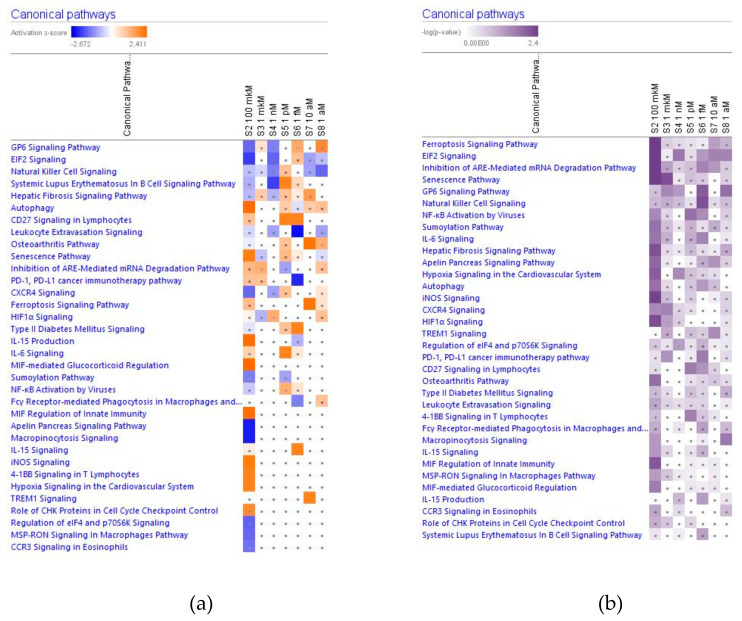
Effect of Rg5 in concentrations of 1 aM, 10 aM, 1 fM, 1 pM, 1 nM, 1 μM and 100 μM) on cellular immune response, stress and injury (including senescence and EIF2 signaling) signaling. (**a**) The brown color depicts predicted activation, blue color—predicted inhibition of signaling pathway; the symbol shows that the activation z-score was <2. (**b**) the symbol shows that the −log *p*-value was <1.3.

**Figure 4:**
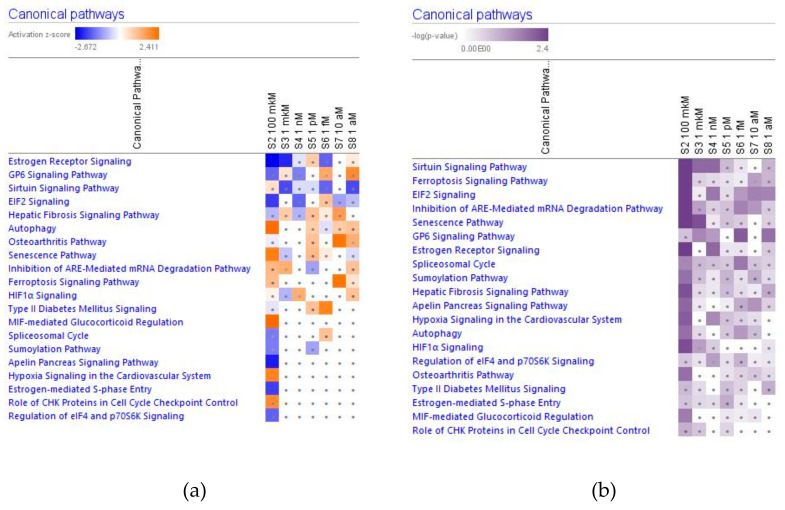
Effect of Rg5 in concentrations of 1 aM, 10 aM, 1 fM, 1 pM, 1 nM, 1 μM and 100 μM) on nuclear receptors and transcriptional regulation (including estrogen receptors and sirtuin) signaling. (**a**) The brown color depicts predicted activation, blue color—predicted inhibition of signaling pathway; the symbol shows that the activation z-score was <2. (**b**) The symbol shows that the −log *p*-value was <1.3.

**Figure 5:**
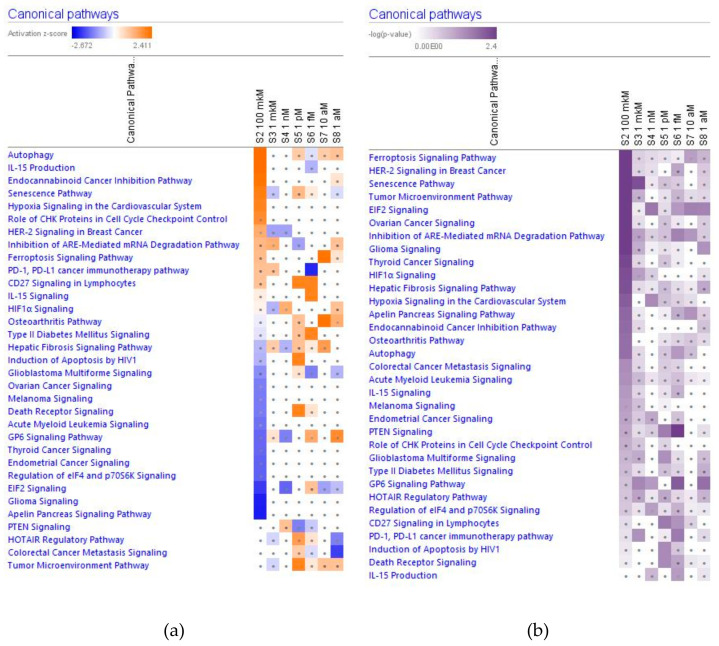
Effect of Rg5 in concentrations of 1 aM, 10 aM, 1 fM, 1 pM, 1 nM, 1 μM and 100 μM) on apoptosis and cancer (including death receptor signaling, programmed death PD-1 cancer immunotherapy and tumor microenvironment) canonical pathways. (**a**) Brown color depicts predicted activation, blue color—predicted inhibition of signaling pathway; symbol shows that the activation z-score was <2. (**b**) Symbol shows that the −log *p*-value was <1.3.

In Figure 2, Figure 3, Figure 4 and Figure 5 the effect (−log *p*-value > 1.3, z-score > 2) of various concentrations of ginsenoside Rg5 on deregulated intracellular signaling canonical pathways are presented: neurotransmitters and nervous system signaling (including neuroinflammation, and CREB signaling in neurons) (Figure 2); cellular immune response, stress and injury signaling (including senescence and EIF2 signaling) (Figure 3); nuclear receptors and transcriptional regulation signaling (including estrogen receptors and sirtuin signaling) (Figure 4); apoptosis and cancer (including death receptors, PD-1 cancer immunotherapy, and tumor microenvironment signaling) canonical pathways (Figure 5).

#### 2.2.1. Neurotransmitters and Nervous System Signaling

##### The Neuroinflammation Signaling Pathway

**Figure 6:**
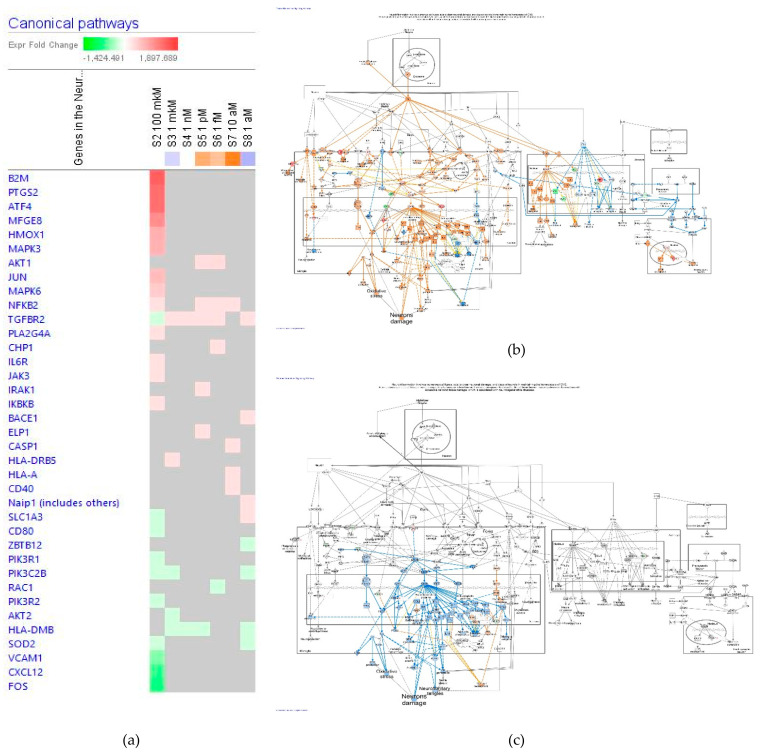
Effect of Rg5 on neuroinflammation signaling pathway: (**a**) Heatmap of gene expression (in fold changes compared to control, red, upregulation and green, downregulation), after exposure with ginsenoside Rg5 at different concentrations; the 100 μM signature is shown in the leftmost column as solid red or green squares indicating genes that are upregulated or downregulated, respectively; color intensity indicates the actual log-fold changes; (**b**) Rg5 in the concentration of 100 μM—predicted inhibition (blue) and activation (brown); (**c**) Rg5 at a concentration of 1 μM—predicted inhibition (blue) and activation (brown).

The effect of ginsenoside Rg5 in all tested concentrations (1 aM, 10 aM, 1 fM, 1 pM, 1 nM, 1 μM, and 100 μM) on gene expression involved in the neuroinflammation signaling pathway is shown in Figure 6a.

Figure 6b,c present the predicted activation and inhibition of the pathway at concentrations of 100 μM and 1 μM Rg5, respectively. The effects of Rg5 at all other tested concentrations are included in Supplement 3 in detail.

Table 2 demonstrates some primary endpoints of the neuroinflammation signaling pathway and the number of genes matching the pathway for predicting positive (+) or negative (−) effects of Rg5 in various diseases and cellular processes associated with neuroinflammation.

It is noteworthy that Rg5 likely inhibits the neuronal damage, amyloid β clearance and plague accumulation, blood-brain barrier disruption, major depression, and reactive oxygen species production both in acto-molar (10^−18^ M) and micromolar (10^−6^ M) concentrations, and induced reversal effect in the highest toxic concentration (10^−4^ M).

##### The cAMP/CREB Signaling Pathway in Neurons

**Figure 7:**
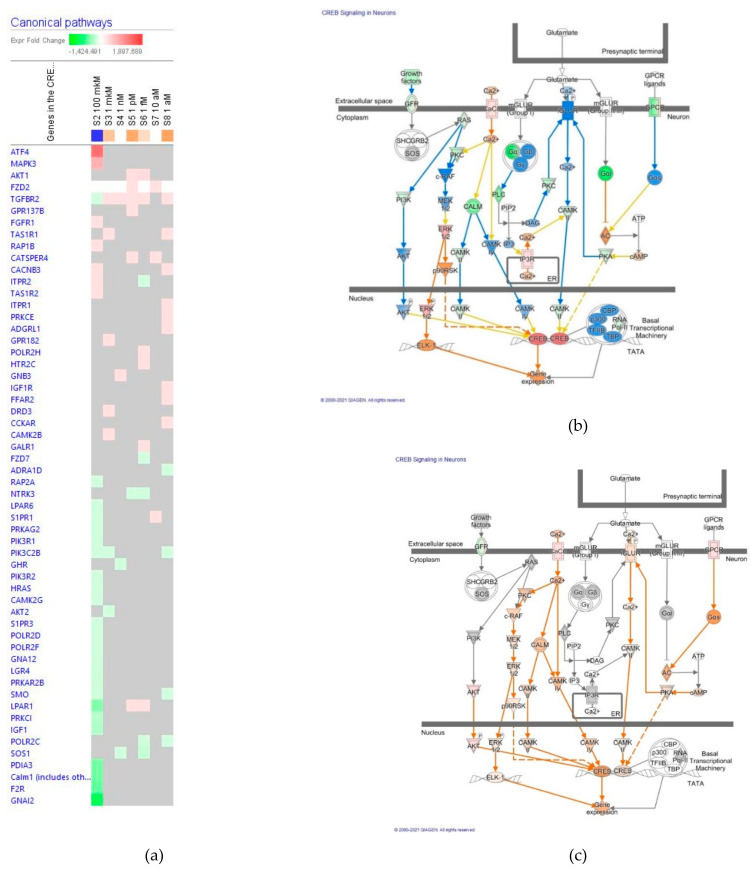
Effect of Rg5 on CREB signaling pathway: (**a**) Heatmap of gene expressions (in fold changes compared to control, red, upregulation and green, downregulation), after exposure with ginsenoside Rg5 at different concentrations; the 100 μM signature is shown in the leftmost column as solid red or green squares indicating genes that are upregulated or downregulated, respectively; color intensity indicates the actual log-fold changes; (**b**) Rg5 at a concentration of 100 μM—predicted inhibition (blue) and activation (brown); (**c**) Rg5 at a concentration of 1 μM—predicted inhibition (blue) and activation (brown).

The effect of ginsenoside Rg5 in all tested concentrations (1 aM, 10 aM, 1 fM, 1 pM, 1 nM, 1 μM and 100 μM) on gene expression involved in cAMP/CREB signaling pathway is shown in Figure 7a. Figure 7b,c present the predicted activation and inhibition of the pathway at concentrations 100 μM and 1 μM Rg5, respectively. The effects of Rg5 on CREB signaling pathway gene expression at all other tested concentrations are included in Supplement 4 in detail.

#### 2.2.2. Cellular Immune Response, Cellular stress and Injury Signaling

##### Senescence

**Figure 8:**
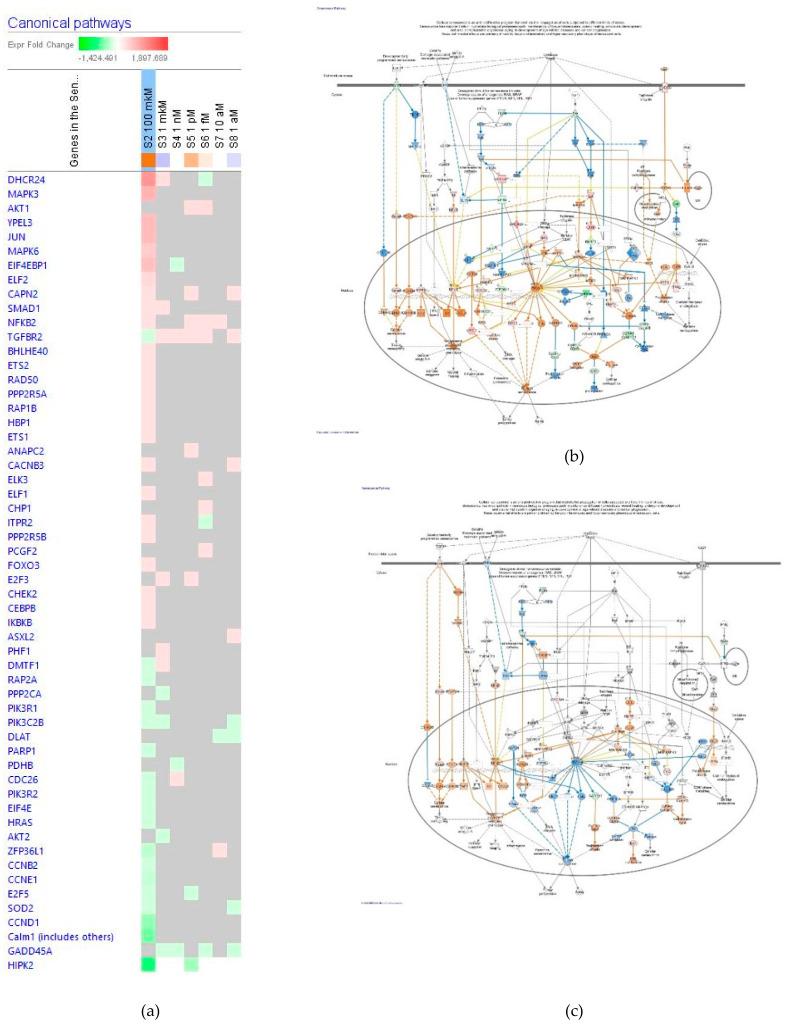
Effect of Rg5 on senescence signaling pathway: (**a**) Heatmap of gene expression (in fold changes compared to control, red, upregulation and green, downregulation), after exposure with ginsenoside Rg5 at different concentrations; the 100 μM signature is shown in the leftmost column as solid red or green squares indicating genes that are upregulated or downregulated, respectively; color intensity indicates the actual log-fold changes; (**b**) Rg5 at a concentration of 100 μM—predicted inhibition (blue) and activation (brown); (**c**) Rg5 at a concentration of 1 μM—predicted inhibition (blue) and activation (brown).

The effect of ginsenoside Rg5 at all tested concentrations (1 aM, 10 aM, 1 fM, 1 pM, 1 nM, 1 μM and 100 μM) on gene expression involved in the senescence signaling pathway is shown in Figure 8a. Figure 8b,c present the predicted activation and inhibition of the pathway at concentrations 100 μM and 1 μM Rg5, respectively. The effects of Rg5 at all other tested concentrations are included in Supplement 5 in detail.

Table 3 shows some primary endpoints of the estrogen receptors signaling pathway and the number of genes matching the pathway for predicting positive (+) or negative (−) effects of Rg5 in cellular functions associated with senescence signaling. Importantly, Rg5 at concentrations of 10^−6^–10^−18^ M activated the cell division cycle and inhibited cellular senescence, while at the concentration of 10^−4^ M reverse effects are noticed.

##### Eukaryotic Translation Initiation Factor EIF2 Signaling

**Figure 9:**
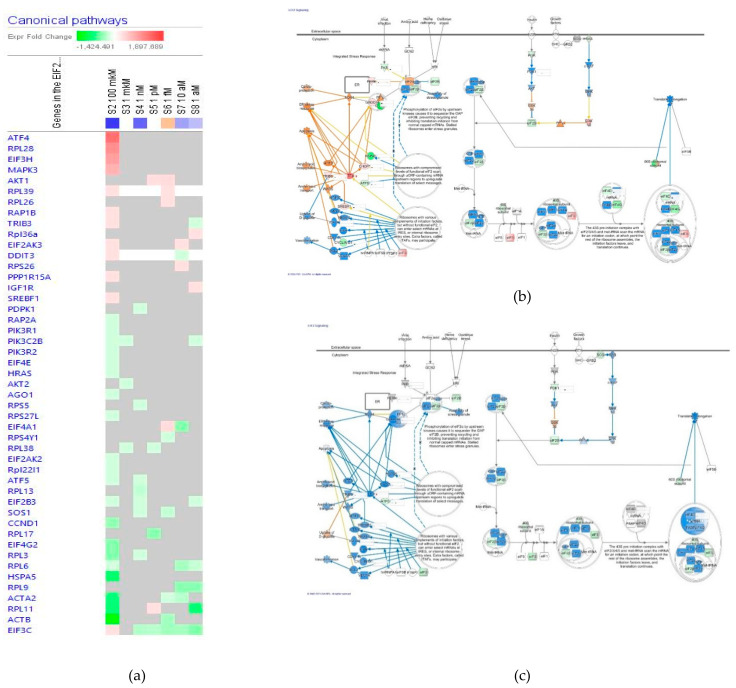
Effect of Rg5 on EIF2 signaling pathway: (**a**) Heatmap of gene expression (in fold changes compared to control, red, upregulation and green, downregulation), after exposure with ginsenoside Rg5 in different concentrations; the 100 μM signature is shown in the leftmost column as solid red or green squares indicating genes that are upregulated or downregulated, respectively; color intensity indicates the actual log-fold changes; (**b**) Rg5 at a concentration of 100 μM—predicted inhibition (blue) and activation (brown); (**c**) Rg5 at a concentration of 1 μM—predicted inhibition (blue) and activation (brown).

The effect of ginsenoside Rg5 at all tested concentrations (1 aM, 10 aM, 1 fM, 1 pM, 1 nM, 1 μM and 100 μM) on gene expression involved in the EIF2 signaling pathway is shown in Figure 9a. Figure 9b,c present predicted activation and inhibition of the pathway at concentrations of 100 μM and 1 μM Rg5, respectively. The effects of Rg5 at all other tested concentrations are included in Supplement 6 in detail. Table 4 shows some primary endpoints of the EIF2 signaling pathway and the number of genes matching the pathway for predicting positive (+) or negative (−) effects of Rg5 in various diseases and cellular functions associated with the EIF2 signaling.

Notably, Rg5 positively regulates glucose uptake and negatively endoplasmic reticulum stress response at all low concentrations from 10^−9^ M, and the reversal effect at the concentration of 10^−4^ M.

#### 2.2.3. Nuclear Receptors Signaling and Transcriptional Regulators Signaling

##### Estrogen Receptors Signaling

**Figure 10:**
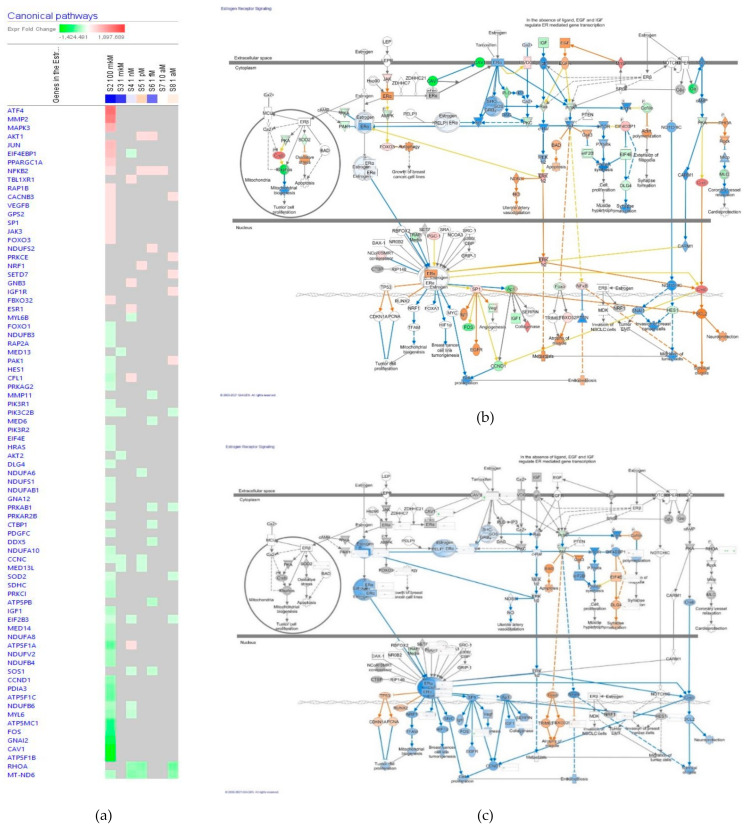
Effect of Rg5 on estrogen receptors signaling pathway: (**a**) Heatmap of gene expression (in fold changes compared to control, red, upregulation and green, downregulation), after exposure with ginsenoside Rg5 at different concentrations; the 100 μM signature is shown in the leftmost column as solid red or green squares indicating genes that are upregulated downregulated, respectively; color intensity indicates the actual log-fold changes; (**b**) Rg5 in the concentration of 100 μM—predicted inhibition (blue) and activation (brown); (**c**) Rg5 in the concentration of 1 μM—predicted inhibition (blue) and activation (brown).

The effect of ginsenoside Rg5 at all tested concentrations (1 aM, 10 aM, 1 fM, 1 pM, 1 nM, 1 μM and 100 μM) on gene expression involved in estrogen receptors signaling pathway is shown in Figure 10a. Figure 10b,c present predicted activation and inhibition of the pathway at concentrations of 100 μM and 1 μM Rg5, respectively. The effects of Rg5 at all other tested concentrations are included in Supplement 7 in detail.

Table 5 outlines some primary endpoints of the estrogen receptors signaling pathway and the number of genes matching the pathway for predicting positive (+) or negative (−) effects of Rg5 in various diseases and cellular functions associated with estrogen receptors signaling.

Notably, Rg5 is expected to increase neuroprotection and survival of cells in a wide range of concentrations from 10^−9^ to 10^−18^ M, but inhibits muscle atrophy and apoptosis at the concentrations from 10^−9^ to 10^−15^ M.

##### Sirtuin Signaling Pathway

**Figure 11:**
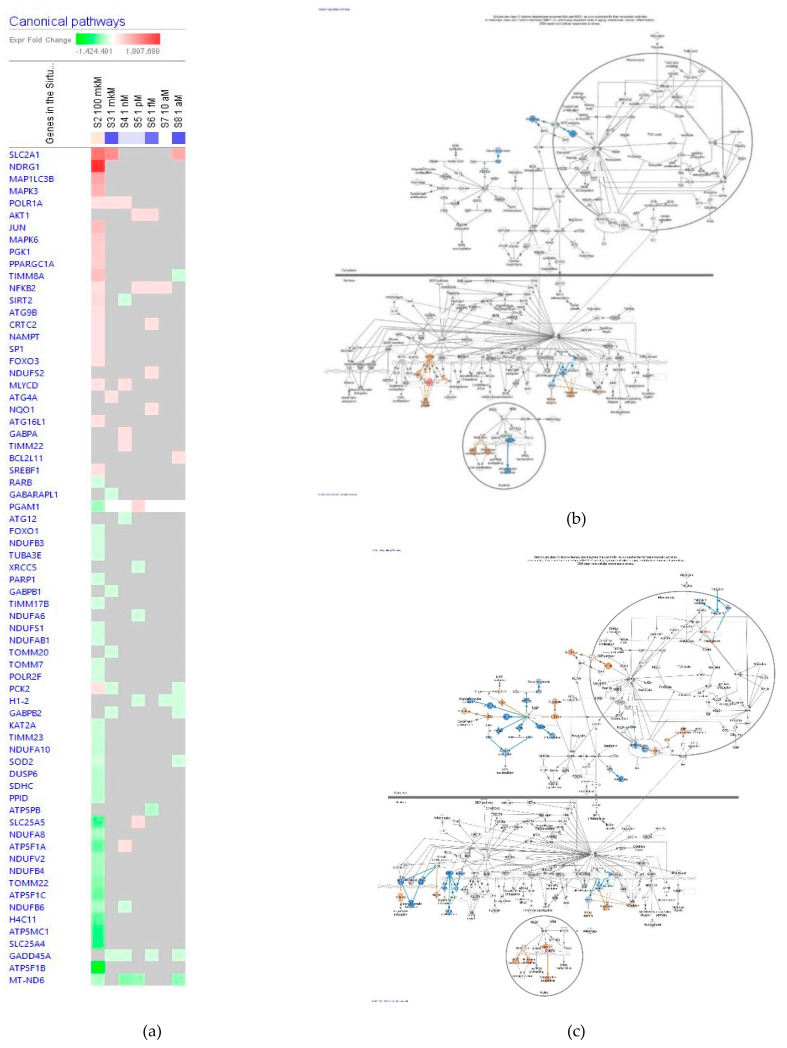
Effect of Rg5 on SIRT signaling pathway in neurons: (**a**)—Heatmap of gene expressions (in fold changes compared to control, red, upregulation and green—downregulation), after exposure of neurons with ginsenoside Rg5 in different concentrations; the 100 μM signature is shown in the leftmost column as solid red or green squares indicating genes that are upregulated or down-regulated, respectively; color intensity indicates the actual log-fold changes; (**b**) Rg5 in the concentration of 100 μM—predicted inhibition (blue) and activation (brown); (**c**) Rg5 in the concentration of 1 nM—predicted inhibition (blue) and activation (brown).

The effect of ginsenoside Rg5 at all tested concentrations (1 aM, 10 aM, 1 fM, 1 pM, 1 nM, 1 μM and 100 μM) on gene expression involved in SIRT signaling pathway is shown in Figure 11a. Figure 11b,c present the predicted activation and inhibition of the pathway at concentrations of 100 μM and 1 μM Rg5, respectively. The effects of Rg5 on SIRT signaling pathway gene expression at all other tested concentrations are included in Supplement 8 in detail.

#### 2.2.4. Apoptosis and Cancer Signaling

##### Death Receptor Signaling

**Figure 12:**
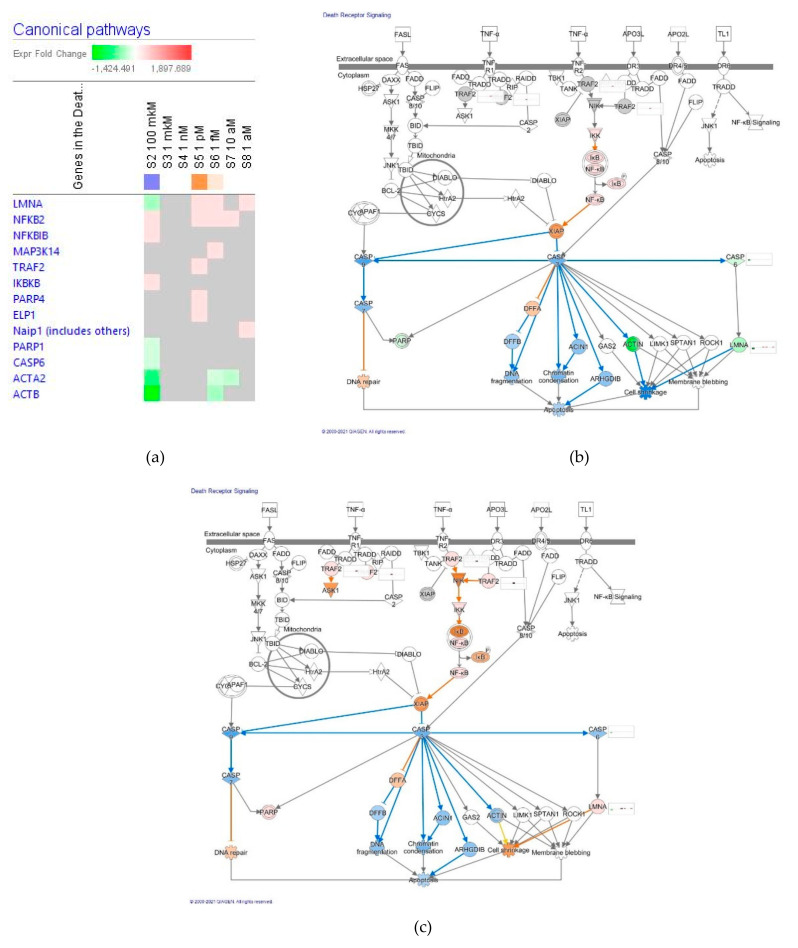
Effect of Rg5 on death receptors signaling pathway: (**a**) Heatmap of gene expression (in fold changes compared to control, red, upregulation and green, downregulation), after exposure with ginsenoside Rg5 at different concentrations; the 100 μM signature is shown in the leftmost column as solid red or green squares indicating genes that are upregulated or downregulated, respectively; color intensity indicates the actual log-fold changes; (**b**) Rg5 at a concentration of 100 μM—predicted inhibition (blue) and activation (brown); (**c**) Rg5 at a concentration of 1 μM—predicted inhibition (blue) and activation (brown).

The effect of ginsenoside Rg5 at all tested concentrations (1 aM, 10 aM, 1 fM, 1 pM, 1 nM, 1 μM and 100 μM) on gene expression involved in the death receptors signaling pathway is shown in Figure 12a.

Figure 12b,c present predicted activation and inhibition of the pathway at a concentration of 100 μM and 1 μM Rg5, respectively. The effects of Rg5 at all other tested concentrations are included in Supplement 9 in detail. Table 6 demonstrates some primary endpoints of the death receptors signaling pathway and the number of genes matching the pathway for predicting positive (+) or negative (−) effects of Rg5 in cellular functions associated with apoptosis.

Remarkably, Rg5 inhibits apoptosis, cell shrinkage, chromatin condensation, DNA fragmentation and activates DNA repair at all concentrations from 10^−4^ M to 10^−18^ M.

##### Tumor Microenvironment Pathway

**Figure 13:**
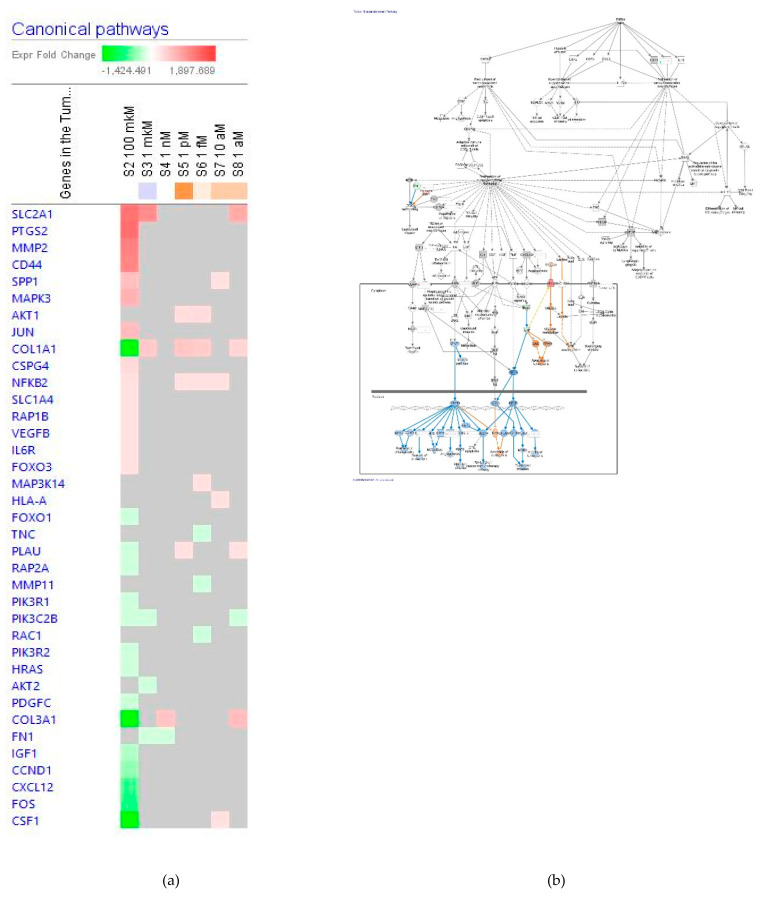
Effect of Rg5 on tumor microenvironment signaling pathway: (**a**) Heatmap of gene expression (in fold changes compared to control, red, upregulation and green, downregulation), after exposure with ginsenoside Rg5 in different concentrations; the 100 mM signature is shown in the leftmost column as solid red or green squares indicating genes that are upregulated or downregulated, respectively; color intensity indicates the actual log-fold changes; (**b**) Rg5 at a concentration of 1 μM—predicted inhibition (blue) and activation (brown).

The effect of ginsenoside Rg5 at all tested concentrations (1 aM, 10 aM, 1 fM, 1 pM, 1 nM, 1 μM and 100 μM) on gene expression involved in tumor microenvironment signaling pathway is shown in Figure 13a. Figure 13b presents the predicted inhibition of the pathway at a concentration 1 μM Rg5. The effects of Rg5 at all other tested concentrations are included in Supplement 10 in detail.

Table 7 demonstrates some primary endpoints of the tumor microenvironment signaling pathway and the number of genes matching the pathway for predicting positive (+) or negative (−) effects of Rg5 in various diseases and cellular functions associated with the tumor microenvironment signaling.

Importantly, Rg5 may activate apoptosis of tumor cells, increase viability and survival of tumor cells and metastasis, whereas proliferation of tumor cells and tumor cell invasion are potentially reduced at the concentration of 10^−4^ M.

##### The Programmed Cell Death Receptor PD-1 Cancer Immunotherapy Pathway

**Figure 14:**
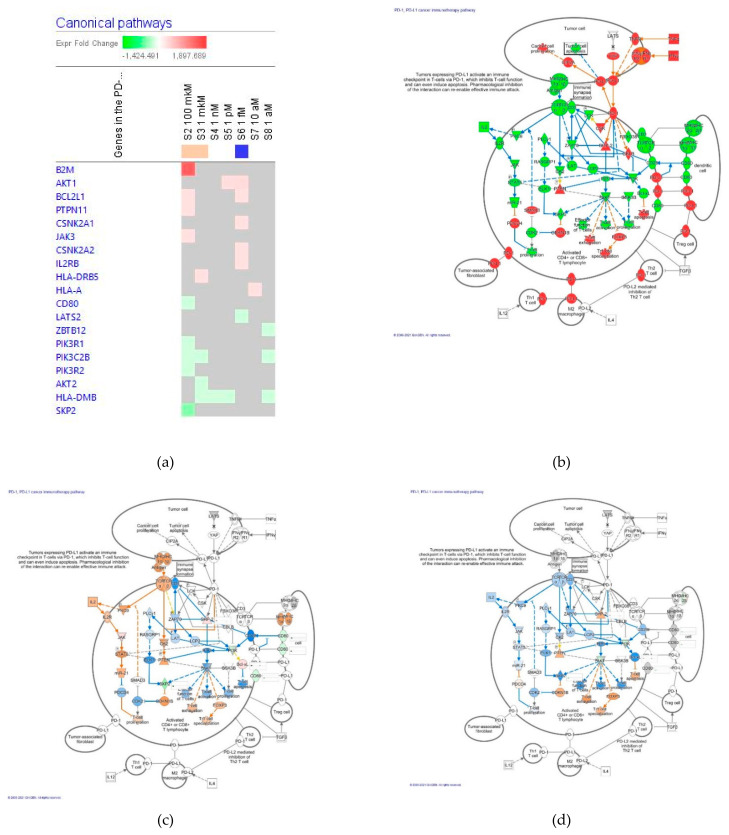
Effect of Rg5 on PD-1 cancer immunotherapy signaling pathway: (**a**) Heatmap of gene expression (in fold changes compared to control, red, upregulation and green, downregulation), after exposure with ginsenoside Rg5 at different concentrations; the 100 μM signature is shown in the leftmost column as solid red or green squares indicating genes that are upregulated or downregulated, respectively; color intensity indicates the actual log-fold changes; (**b**) canonical pathway activation state; (**c**) Rg5 at a concentration of 100 μM—predicted inhibition (blue) and activation (brown); (**d**) Rg5 at a concentration of 1 μM—predicted inhibition (blue) and activation (brown).

The effect of ginsenoside Rg5 at all tested concentrations (1 aM, 10 aM, 1 fM, 1 pM, 1 nM, 1 μM and 100 μM) on gene expression involved in the programmed cell death receptor PD−1 cancer immunotherapy signaling pathway is shown in Figure 14a.

Figure 14b illustrates the predicted activation of the PD-1 cancer immunotherapy signaling pathway, while Figure 14c,d show the effects of Rg5 at concentrations 100 μM and 1 μM, respectively. The effects of Rg5 at all other tested concentrations are included in Supplement 11 in detail. Table 8 outlines some primary endpoints of the apoptosis receptor PD-1 cancer immunotherapy signaling pathway and the number of genes matching the pathway for predicting positive (+) or negative (−) effects of Rg5 in various diseases and cellular functions associated with the PD-1 signaling

Overall, Rg5 activates the PD-1 signaling pathway at concentrations between 10^−4^ and 10^−6^ M.

### 2.3. Effect of Ginsenoside Rg5 on Metabolic Pathways

**Figure 15:**
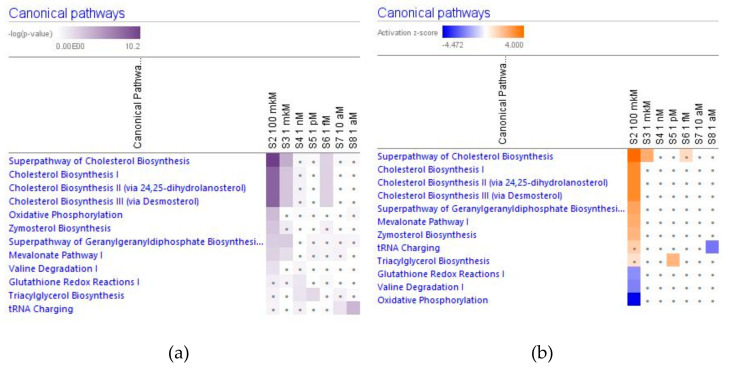
Effect of Rg5 at concentrations of 1 aM, 10 aM, 1 fM, 1 pM, 1 nM, 1 μM and 100 μM) on significantly deregulated metabolic signaling pathways. (**a**) The brown color shows the predicted activation, blue color—predicted inhibition of signaling pathway; the symbol shows that the activation z-score was <2. (**b**) the symbol shows that the −log *p*−value was <1.3.

Figure 15 shows the effect of various concentrations of ginsenoside Rg5 on significantly (−log p-value > 1.3, z-score > 2) deregulated canonical metabolic pathways.

**Figure 16:**
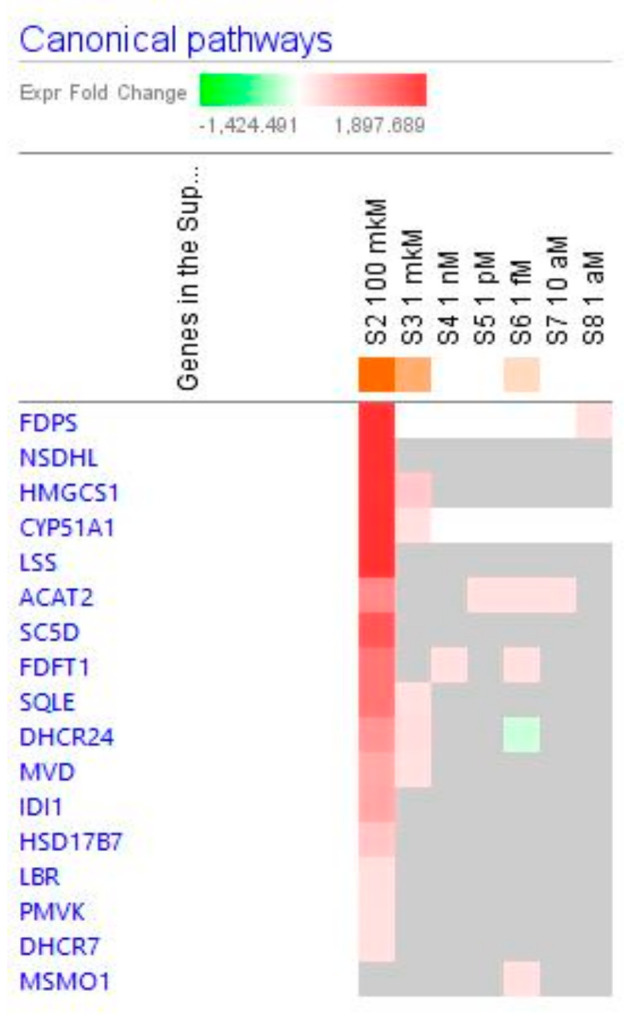
Effect of Rg5 on cholesterol biosynthesis metabolic pathway: Heatmap of gene expression (in fold changes compared to control, red, upregulation and green, downregulation), after exposure with ginsenoside Rg5 at different concentrations; the 100 μM signature is shown in the leftmost column as solid red or green squares indicating genes that are upregulated or downregulated, respectively; color intensity indicates the actual log-fold changes.

Some of them were observed at all tested concentrations, including the cholesterol biosynthesis metabolic super-pathway (Figure 16).

### 2.4. Predicted Effects of Ginsenoside Rg5

#### 2.4.1. Molecular and Cellular Functions

**Figure 17:**
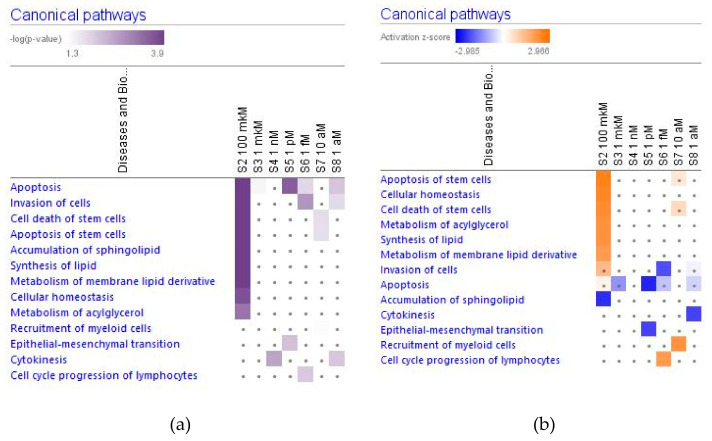
Effect of Rg5 at concentrations of 1 aM, 10 aM, 1 fM, 1 pM, 1 nM, 1 μM and 100 μM) on molecular and cellular functions (including apoptosis of neurons). (**a**) the brown color shows the predicted activation, blue color the predicted inhibition of signaling pathway; the symbol shows that the activation z-score was <2. (**b**) the symbol shows that the −log *p*−value was <1.3.

**Figure 18:**
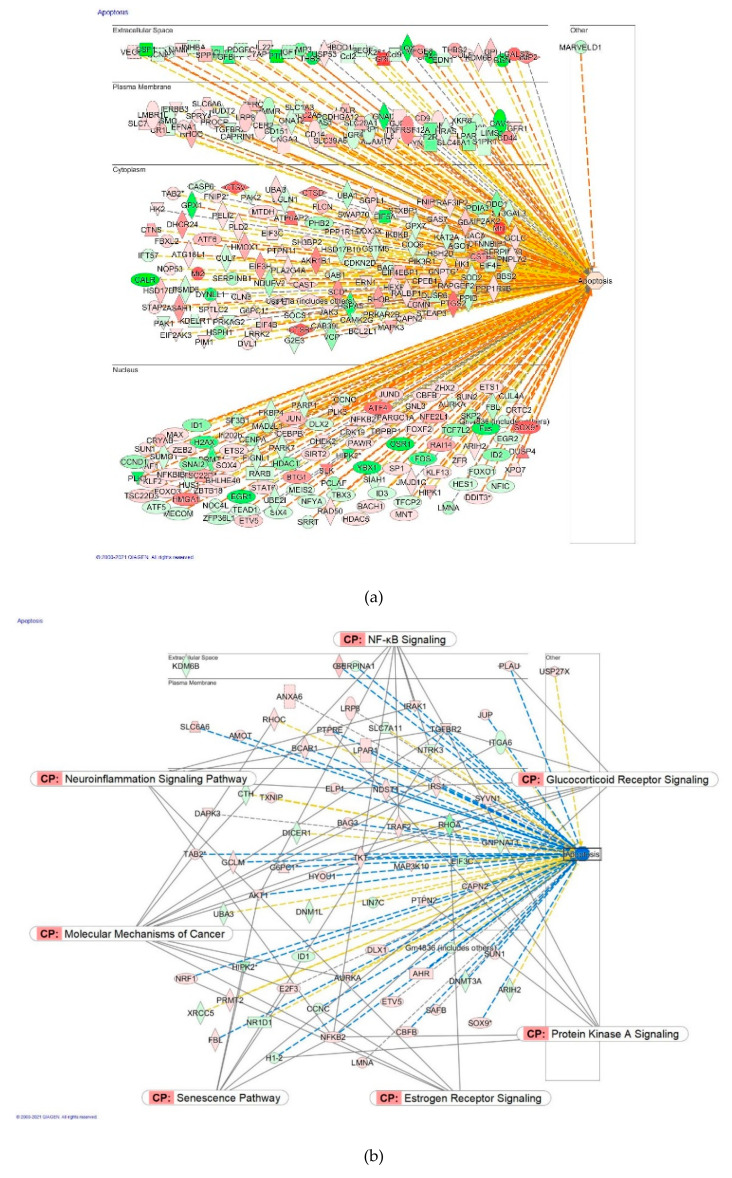
Predicted effects of Rg5 on apoptosis: (**a**) activation (brown) at a concentration of 100 μM; (**b**)—predicted inhibition (blue) at a concentration of 1 pM.

Figure 17 demonstrates that Ginsenoside Rg5 can have a significant impact on some of the cellular functions only at the highest concentration. On the contrary, at low concentrations, Rg5 has no impact on some other cellular functions at the highest concentration but exhibits a significant effect in low concentrations. Rg5 has a trend to inhibit the apoptosis of neuronal cells at a wide range of concentrations with the maximal impact at the concentration of 1 pM. Figure 18 demonstrates the molecular networks of deregulated genes that contribute to the inhibition of apoptosis at the concentration of Rg5 1 pM and the reversal effect at the toxic concentration of 100 μM.

#### 2.4.2. Physiological Functions

**Figure 19:**
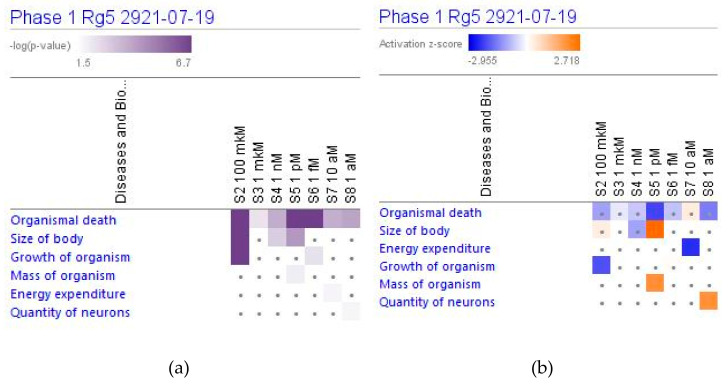
Effect of Rg5 at concentrations of 1 aM, 10 aM, 1 fM, 1 pM, 1 nM, 1 μM and 100 μM) on physiological functions, including organismal death. (**a**) The brown color shows the predicted activation, blue color the predicted inhibition of signaling pathway; the symbol shows that the activation z-score was <2. (**b**) The symbol shows that the −log *p*-value was <1.3.

Ginsenoside Rg5 can have a significant impact on some of the cellular functions only at the highest concentration, Figure 19. On the contrary, at low concentrations, Rg5 has no impact on some other cellular functions at the highest concentration but exhibits a significant effect in low concentrations.

#### 2.4.3. Diseases and Disorders

**Figure 20:**
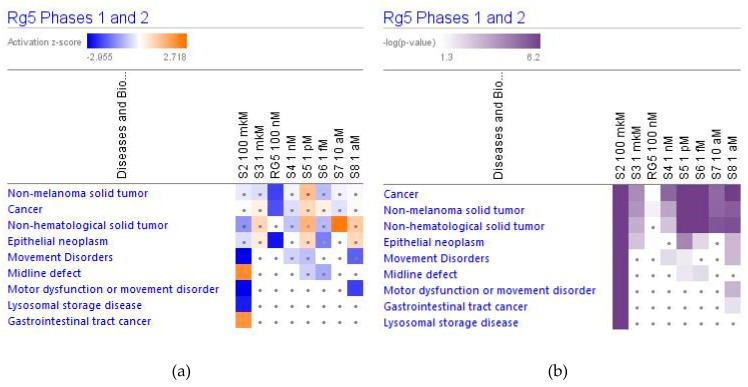
Effect of Rg5 at concentrations of 1 aM, 10 aM, 1 fM, 1 pM, 1 nM, 100 nM *, 1 μM and 100 μM) on diseases and disorders, including movement disorders. (**a**) The brown color shows the predicted activation, blue color the predicted inhibition of signaling pathway; the symbol shows that the activation z-score was <2. (**b**) the symbol shows that the −log *p*-value was <1.3. *: Results from Network Pharmacology of Red Ginseng (Part II).

Figure 20 shows that ginsenoside Rg5 can have a significant impact on some types of cancer and movement disorders both at low and high concentrations.

**Figure 21:**
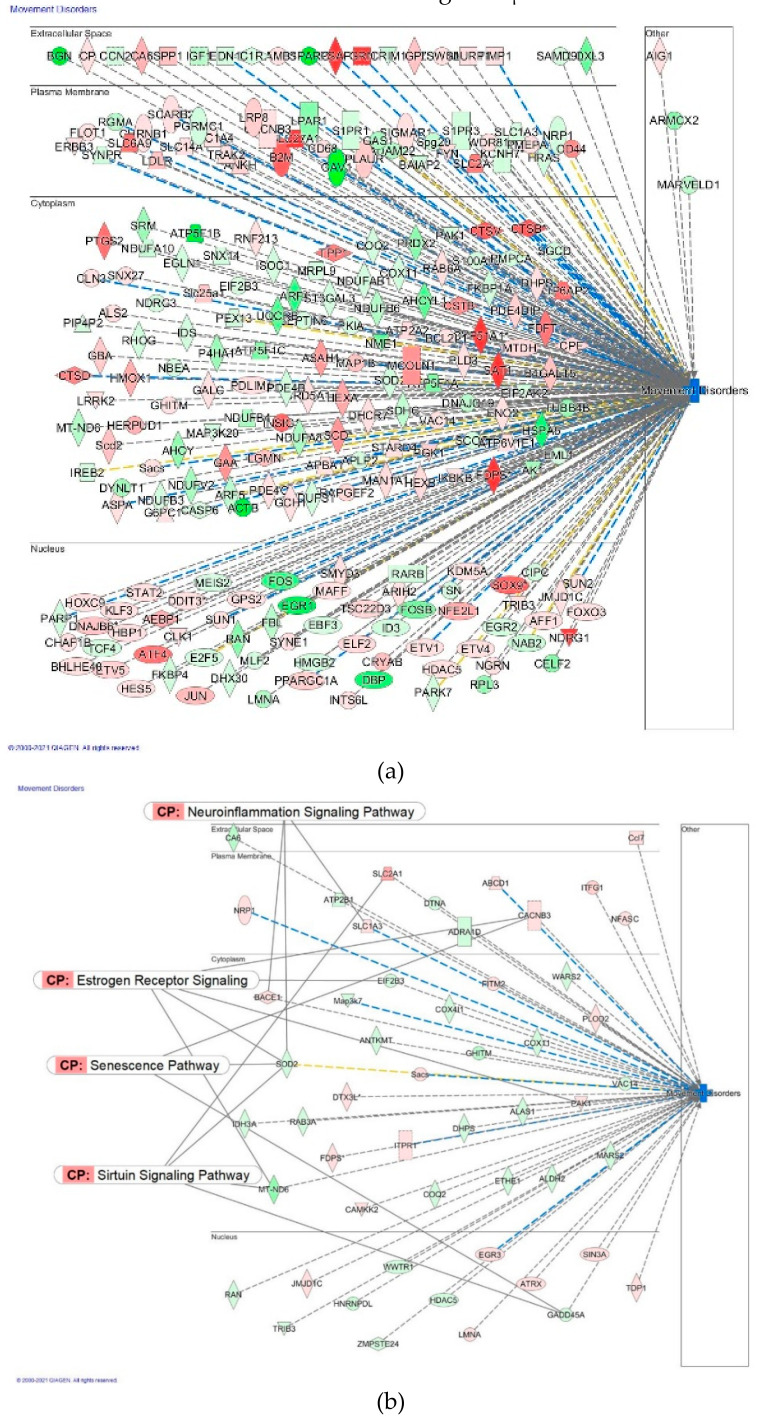
Predicted effects of Rg5 on movement disorders: (**a**) activation (brown) at a concentration of 100 μM; (**b**) predicted inhibition (blue) at a concentration of 1 aM.

**Figure 22:**
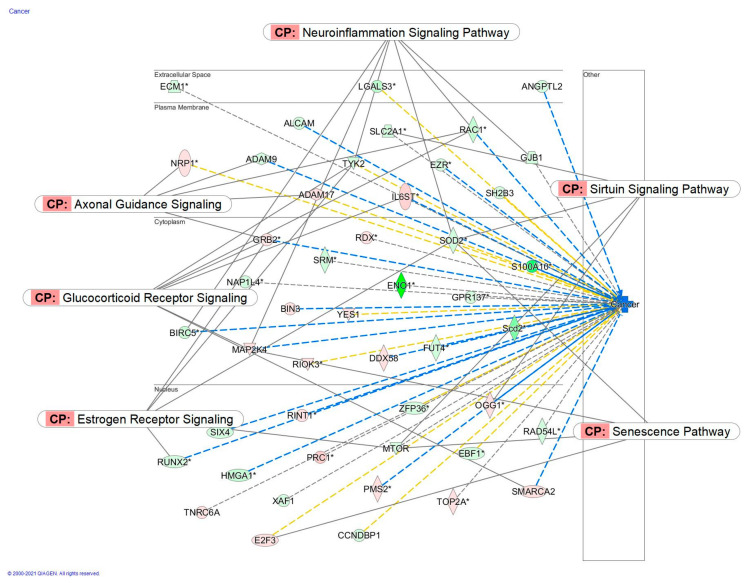
Predicted effects of Rg5 in cancer: predicted inhibition (blue) at the concentration of 100 nM. *: Results from Network Pharmacology of Red Ginseng (Part II).

Molecular networks of deregulated genes that contribute to the movement disorders and cancer at the concentration of Rg5 100 μM and 1 aM are sown in Figure 21 and Figure 22.

## 3. Discussion

Results of available pharmacological studies of ginsenoside Rg5 conducted in several in vitro (Supplemental Table S1 in Supplement 1) and in vivo (Supplemental Table S6 in Supplement 1) experimental animal models of neurological, inflammatory, metabolic disorders, and cancer have been recently reviewed [54]. It was concluded that ginsenoside Rg5 has the potential as an anticancer and anti-inflammatory drug, particularly in neurodegenerative diseases including mild cognitive impairments, Alzheimer’s disease, Huntington’s disease, and Parkinson’s disease.

In all of these animal studies [16,32,34,35,36,39,43,55,56,57,58], ginsenoside Rg5 was active at a dose of 10 mg/kg BW, which corresponds to a human dose of 5 g Red Ginseng dry root containing 2% Rg5 (Supplemental Table S6 in Supplement 1).

The maximal concentration of Rg5 in the blood of human subjects taking ginseng orally at a dose of 9 g was 2–10 nM [44]. Ginsenoside Rg5 protected neuronal cells from glutamate-induced apoptosis in an in vitro Huntington’s disease assay at a concentration of 1000 nM [32] and exhibited a neuroprotective effect in heat stress-induced apoptosis at concentrations of 26–52 μM in experiments with murine hippocampal neuronal HT22 cells, but was toxic at concentrations of 78–104 μM [33]. In our study, ginsenoside Rg5 was pharmacologically active both at micro-molar and nano-molar concentrations as well as in a wide/broad range of physiological sub-physiological concentrations from pico-molar (10^−12^ M) to act-molar (10^−18^ M), significantly changing the expression (in about 300−800−fold change) of almost the same number of genes (370 ± 69; RSD = 18.53%) (Table 1, Figure 1). The broad range concentration—gene expression relationships were not linear for many genes. Some of them were reversed at the toxic concentration of 100 μM (Figure 1b) if the number of Rg5 molecules was over 2 × 10^9^ per one neuronal cell (Table 1, Figure 1).

Considering that the blood level of ginsenoside Rg5 is not steady and varies over time during absorption and clearance for 24–72 h after repeated oral administration, the target cells of various tissues, including brain tissues, are continuously exposed to different doses/concentrations of ginsenoside Rg5. In this context, the difference in gene expression profiles to varying concentrations of Rg5 and predicted effects on signaling and metabolic pathways, molecular networks, cellular, physiological functions in various concentrations of Rg5 (Figure 2, Figure 3, Figure 4, Figure 5, Figure 6, Figure 7, Figure 8, Figure 9, Figure 10, Figure 11, Figure 12, Figure 13, Figure 14, Figure 15, Figure 16, Figure 17, Figure 18, Figure 19, Figure 20, Figure 21 and Figure 22) was remarkable.

Notably, relatively few genes were deregulated at several low concentrations, and only one gene, *Ca6*, encoding the extracellular enzyme carbonic anhydrase 6 was regulated by ginsenoside Rg5 at all tested concentrations. Rg5 down−regulated *Ca6* at all low concentrations (Figure 1b), suggesting that Rg5 acted like the antihypertensive *Ca6* inhibitors acetazolamide and chlorothiazide [59,60].

Most deregulated genes were specific at every single concentration (concentration-specific gene expression signatures) (Figure 6a, Figure 7a, Figure 8a, Figure 9a, Figure 10a, Figure 11a, Figure 12a, Figure 13a, Figure 14a and Figure 16). This had an impact on the expected activation or inhibition signaling (Figure 2, Figure 3, Figure 4, Figure 5, Figure 6, Figure 7, Figure 8, Figure 9, Figure 10, Figure 11, Figure 12, Figure 13 and Figure 14) and metabolic (Figure 15) canonical pathways, cellular (Figure 17 and Figure 18), and physiological (Figure 19) functions, as well as organismal disorders and diseases (Figure 20, Figure 21 and Figure 22) at various concentrations.

The IPA analyses of the datasets revealed that ginsenoside Rg5 has the potential to activate the biosynthesis of cholesterol (the precursor of all steroid hormones).

Furthermore, Rg5 exhibited predictable beneficial effects in senescence, neuroinflammation, apoptosis, organismal death, movement disorders, and cancer.

Typical features of all “low dose” effects compared to the “high dose” effects were somewhat soft-acting, which was characterized by considerably fewer genes involved in regulating signaling pathways and cellular physiological functions and disorders.

At first glance, some of the results were contradictory to those observed in other studies, where significantly higher (micromolar) concentrations (Supplementary Table S1 in Supplement 1) have been applied, which have no clinical significance simply since the corresponding human doses are far beyond the traditionally used (Supplementary Tables S1 and S4 in Supplement 1). For example, it was reported that ginsenoside Rg5 inhibits NF-kB signaling in macrophages, human epidermal keratinocytes, HeLa, A549, and 293T cancer cells at the concentration 20–50 μM [38,39,40] (Table S1 in Supplement 1), but in our study ginsenoside, Rg5 induces mild upregulation (53- to103--fold) of *Nfkb2* gene expression both at low and highest concentrations (10 aM, 1 fM, 1 pM, and 100 μM).

However, the key point is that the functional role of NF-κB in neurons is different from other cells [61,62]. In neurons, NF-κB signaling plays a role not only in neuroprotection, neurodegeneration, inflammation, and apoptosis but also in neuronal development, learning, memory, and synaptic plasticity [61,62,63]. Activation of constitutive NF-κB is involved in learning and memory processes via activation of PKA signaling molecules [62]. PKA activates P-CREB protein which leads to the release of neurotrophins in the neurons for learning and memory. The inhibition of NF-κB signaling resulted in behavior or memory deficits associated with suppression of CREB phosphorylation [64].

Activation of constituent NF−kB can also prevent the death of neurons by inducing the production of anti-apoptotic proteins such as Bcl-2, IAPs, and manganese superoxide dismutase [61,62]. On the contrary, abnormal activation of inducible NF-κB triggers pro-apoptotic, pro-inflammatory genes encoding caspases and Bax, IL-12 and IL-17, and neurotoxic glutamate and iNOS, triggering further cellular damage [62]. In this context, mild the upregulation of expression of NF-κB is an adaptive (hormetic) stress response of neuronal cells [53,61,62,63,64,65,66] to ginsenoside Rg5, which is typical for adaptogens increasing cell survival [67]. Furthermore, ginsenoside Rg5 activates CREB signaling pathway at four low concentrations (1 aM, 1 fM, 1 pM, 1 μM) that is also in line with the mechanisms discussed above and predicted beneficial effects of ginsenoside Rg5 in neurodegeneration learning and memory.

There are several limitations of this study. One of them is the lack of scientific literature about the direction of correlations between gene expression and physiological function or disease that can be used in Silico analysis for predictions related to some findings in our experiments. The second limitation is related to the number of concentrations points in the dose-response correlation study: more intermediate points in the range of 1000 fold difference will show smooth changes from point to point. Other studies where different experimental outcome measures will be used are required. Finally, clinical studies in predicted diseases in human subjects are needed.

Overall, this is the first evidence of pharmacological activity of ginsenoside Rg5 in concentrations detected in human pharmacokinetic studies.

## 4. Materials and Methods

All materials and methods used in the present study have been described in detail in our previously published studies [68,69]. Therefore, only a short description of mRNA microarray hybridization, and Ingenuity pathway analysis (IPA) is provided below.

### 4.1. mRNA Microarray Hybridization

Murine hippocampal neuronal HT22 cells were seeded and attached for 24 h prior to drug treatment. Cells were then treated for 24 h at various combinations and concentrations of drugs or DMSO as solvent control (0.5%). Total RNA was isolated using the InviTrap^®^ Spin Universal RNA mini kit (250 preps; Stratec Molecular). RNA concentrations were determined using the NanoDrop spectrophotometer (NanoDrop Technologies, Wilmington, Delaware, DE, United States). The quality of total RNA was confirmed by gel analysis using the total RNA Nanochip assay on an Agilent 2100 bioanalyzer (Agilent Technologies, Santa Clara, CA, USA). Only samples with RNA index values > 8.5 were selected for expression profiling. The experiments were performed in duplicate for treated samples and for control samples by the Genomics and Proteomics Core Facility at the German Cancer Research Center in Heidelberg, Germany. For mRNA microarray hybridization the Affymetrix GeneChips^®^ with mouse Clariom S assays have been used according to the manufacturer’s instructions. Data analysis was carry out by normalization of the signals using the quantile normalization algorithm without background subtraction, and differentially regulated genes were defined by calculating the standard deviation differences of a given probe in a one-by-one comparison of samples or groups. The data were further processed using Chipster software, version 4 (The Finnish IT Center for Science CSC).

### 4.2. Ingenuity Pathway Analysis (IPA)

Microarray data were analyzed by the Ingenuity Pathways Analysis (IPA) software, Summer release 2021 (QIAGEN Bioinformatics, Aarhus C, Denmark). Using IPA, we performed different predictive algorithmic calculations on transcriptomic datasets, resulting in varying analyses, including (i) canonical pathways, which displayed the molecules of interest within well-established signaling or metabolic pathways; and (ii) upstream analyses, which predicted the upstream regulators (any molecule that can influence the transcription or expression of another molecule) that might be activated or inhibited to explain the expression changes in test datasets.

The interpretation of gene expression data was facilitated by consideration of prior biologic knowledge. IPA software relies on the Ingenuity Knowledge Base, a frequently updated database containing biologic and chemical interactions and functional annotations with a large gathering of observations with more than 8.1 million findings manually curated from the biomedical literature or integrated from 45 third-party databases. The network contains 40,000 nodes that represent mammalian genes, molecules, and biologic functions. Nodes are linked by 1,480,000 edges representing experimentally observed cause−effect relationships that relate to gene expression, transcription, activation, molecular metabolism, and binding. Network edges are also associated with a direction (either activating or inhibiting) of the causal effect [70].

To obtain information about the impact of test samples on cellular signaling pathways and networks for biologic functions and diseases downstream of the genes, whose expression has been altered in a dataset, we used the IPA Core Analysis tool for all tested datasets. Analysis of transcriptomic data enabled us to predict regulators that are activated or inhibited based on the distinct up- and downregulation patterns of the expressed genes, and to determine which causal relationships previously reported in the literature are likely to be relevant for the biologic mechanisms underlying the data.

### 4.3. Statistical Analysis

Two statistical methods of analysis of gene expression data were used in Ingenuity Pathway analysis: (a) Gene-set-enrichment method, where differentially expressed genes are intersected with sets of genes that are associated with a particular biological function or pathway providing an ‘enrichment’ score [Fisher’s exact test *p*-value] that measures overlap of observed and predicted regulated gene sets [71,72]; (b) The method that based on previously observed cause-effect relationships related to the direction of effects reported in the literature [73,74] providing so-called z-scores assessing the match of observed and predicted up/down-regulation patterns [70]. The predicted (z-score > 2, or −log FET *p*-value >1.3) effects are based on changes of gene expression in the experimental samples relative to the control.

## 5. Conclusions

For the first time, we have demonstrated that ginsenoside Rg5 exhibits soft-acting effects in a wide range of physiological concentrations and a strong reversal impact at the highest (toxic) concentrations on gene expression of neuronal cells.

Network pharmacology analyses of genes expression profiles using IPA software showed that ginsenoside Rg5 have the potential to activate the biosynthesis of cholesterol (the precursor of all steroid hormones), and to exhibit predictable effects in neuroinflammation, senescence, apoptosis, and immune response, suggesting soft-acting, beneficial impact on organismal death, movement disorders, and cancer (carcinoma, genitourinary tumor, solid malignant tumor, and sarcoma).

## Figures and Tables

**Table 1:** Number of genes deregulated^1^ by ginsenoside Rg5 in different concentrations in the murine hippocampal neuronal cell line HT22. For details, see Supplemental Table S3 in Supplement 2.

Sample Name	Rg5 Concentration	Molecules of Rg5 per Cells, Ratio	Number of Deregulated Genes	Number of Deregulated Genes Unique Only in Selected Conc.	Fold Changes of the Only Gene, *Ca6*, Which is Deregulated in All Conc.
S2	10^−4^ M	^1^ 2 × 10^11^	1670	1215	731
S3	10^−6^ M	^1^ 2 × 10^9^	280	120	−306
S4	10^−9^ M	^1^ 2 × 10^6^	328	122	−286
S5	10^−12^ M	^1^ 2 × 10^3^	380	163	−274
S6	10^−15^ M	^1^ 2	471	252	−281
S7	10^−17^ M	2:10^2^	343	159	−293
S8	10^−18^ M	2:10^3^	422	178	−313

^1^ >20-fold compared to control.

**Table 2:** The number of genes matching neuroinflammation signaling pathway and predicted effects of Rg5 in various diseases and cellular processes associated with neuroinflammation ^1^.

Concentration of Rg5, M		10^−4^	10^−6^	10^−9^	10^−12^	10^−15^	10^−17^	10^−18^
No. of matching genes		23	5	2	6	5	4	8
Amyloid-β plaque accumulation	disease	+	0	−	−	0	+	−
Astrogliosis	disease	+	−	+	+	+	+	−
Aβ formation/generation	disease	+	−	+	+	+	+	+
Blood-brain barrier disruption	disease	+	−	+/−	+	+	+	−
Major depression	disease	+	−	+	+	+	+	−
Oxidative stress	disease	+	−	−	+	−	+	−
Neuron’s damage	disease	+	−	+	+	+	+	−
Neuron’s survival	process	−	−	+	+	+	+	+
Neuron’s apoptosis	process	+	0	−	− +	+	+	+ −
Th1 cell recruitment	process	−	+	−	−	−	−	+
T cell recruitment	process	+	0	−	+	+	+	−
GABAergic neuron density	process	+	−	+	+	+	+	−
Amyloid-β precursor protein	protein	+	0	−	−	0	+	−

^1^ 0—no effect; (+)—activation, (−)—inhibition compared to control.

**Table 3:** The number of genes matching senescence signaling pathway and predicted effects of Rg5 in cellular processes associated with neurotransmission ^1^.

Concentration of Rg5, M		10^−4^	10^−6^	10^−9^	10^−12^	10^−15^	10^−17^	10^−18^
No. of matching genes		43	10	5	8	9	3	8
Cell division cycle	function	−	+	+	+	+	+	+
Cellular senescence	function	+	−	−	−	−	−	−

^1^ 0—no effect; (+)—activation, (−)—inhibition compared to control.

**Table 4:** The number of genes matching EIF2 signaling pathway and predicted effects of Rg5 in various diseases and cellular functions associated with intracellular signaling ^1^.

Concentration of Rg5, M		10^−4^	10^−6^	10^−9^	10^−12^	10^−15^	10^−17^	10^−18^
No. of matching genes		32	3	8	5	9	8	9
Cardio-protection	disease	+	0	−	−	−	−	−
ER stress response	function	+	0	−	−	−	−	−
Uptake of D-glucose	function	−	0	+	+	+	+	+
Vascularization	function	−	0	−	−	0	0	+
Assembly of stress granule	function	−	0	−	−	0	0	−
Translation/protein elongation	function	−	−	−	−	+	−	−

^1^ 0—no effect; (+)—activation, (−)—inhibition compared to control.

**Table 5:** The number of genes matching estrogen receptors signaling pathway and predicted effects of Rg5 in various diseases and cellular functions associated with neurotransmission ^1^.

Concentration of Rg5, M		10^−4^	10^−6^	10^−9^	10^−12^	10^−15^	10^−17^	10^−18^
No. of matching genes		56	5	13	8	10	1	11
Atrophy of muscle	disease	+	+	−	−	−	0	+
Breast cancer cell line tumorigenesis	disease	0	−	+	0	−	0	+
Metastasis	disease	+	0	−	0	−	0	0
Oxidative stress	disease	+	0	0	0	0	0	+
Tumor cell proliferation	disease	0	−	+	0	−	0	+0
Angiogenesis	function	0	−	+	+	−	0	+
Apoptosis	function	+	+	−	−	−	0	+
Cell proliferation	function	−	−	+	0	−	0	+
Coronary vessel relaxation	function	−	0	−	+	0	0	+
Neuroprotection	function	+	−	+	+	+	+	+
Survival of cells	function	+	−	+	+	+	+	0
Synapse maturation	function	−	+	+	−	−	0	+

^1^ 0—no effect; (+)—activation, (−)—inhibition compared to control.

**Table 6:** The number of genes matching senescence signaling pathway and predicted effects of Rg5 in cellular processes associated with apoptosis ^1^.

Concentration of Rg5, M		10^−4^	10^−6^	10^−9^	10^−12^	10^−15^	10^−17^	10^−18^
No. of matching genes		8	0	0	5	5	2	2
Apoptosis	function	−	0	0	−	−	−	−
Cell shrinkage	function	−	0	0	−	−	−	+
Chromatin condensation	function	−	0	0	−	−	−	−
DNA fragmentation	function	−	0	0	−	−	−	−
DNA repair	function	+	0	0	+	+	+	+

^1^ 0—no effect; (+)—activation, (−)—inhibition compared to control.

**Table 7:** The number of genes matching tumor microenvironment signaling pathway and predicted effects of Rg5 in various diseases and cellular processes associated with neurotransmission ^1^.

Concentration of Rg5, M		10^−4^	10^−6^	10^−9^	10^−12^	10^−15^	10^−17^	10^−18^
No. of matching genes		29	5	2	4	7	4	5
Apoptosis of tumor cells	Disease	− +	++	0	− −	− −	− +	− +
Viability of tumor cells	function	+	−	0	+	+	+	+
Survival of tumor cells	disease	+	−	0	+	+	+	+
Proliferation of tumor cells	disease	−	−	0	+	+	+	+
Metastasis	disease	+	−	0	+	+	+	+
Tumor cell invasion	disease	−	−	0	+	+	+	+

^1^ 0—no effect; (+)—activation, (−)—inhibition compared to control.

**Table 8:** The number of genes matching apoptosis receptor PD−1 cancer immunotherapy signaling pathway and predicted effects of Rg5 in various diseases and cellular processes associated with cancer immunotherapy ^1^.

Concentration Rg5, M		10^−4^	10^−6^	10^−9^	10^−12^	10^−15^	10^−17^	10^−18^
No. of matching genes		9	4	1	2	6	1	3
Cancer cell proliferation	disease	+	−	−	0	+	+	+
T-cell exhaustion	disease	+	+	0	−	−	0	+
Effector function of T cells	function	−	−	0	+	+	0	−
T-cell apoptosis	function	−	+	0	0	−	−	+
T-cell proliferation	function	−	−	0	+	+	0	0
T-cell activation	function	−	−	0	+	+	0	−
Tr1 cell specialization	function	+	+	0	−	−	0	+

^1^ 0—no effect; (+)—activation, (−)—inhibition compared to control.

## Data Availability

Data is contained in the article and Supplementary Materials.

## Supplementary Materials

The following are available online at https://www.mdpi.com/article/10.3390/ph14100999/s1, Supplementary data 1–11.
