# Supplementary material for: Network Pharmacology of Red Ginseng (Part I): Effects of Ginsenoside Rg5 at Physiological and Sub-Physiological Concentrations
Source: Pharmaceuticals (Basel). 2021 Sep 29;14(10):999. doi: 10.3390/ph14100999 (PMC8537973; doi:10.3390/ph14100999)
Supplement: Supplementary file 1 [file pharmaceuticals-14-00999-s001.zip › Supplement 1.pdf]

Supplement 1.

Table S1. The pharmacological activity of Ginsenoside Rg5 in *in vitro* experiments

Reference	Gin-seno-sides	In vitro conc. µM	In vitro conc. µg/ml	Cells	Results
Wu et al, 2009	**Rg5**<br><br>**Rb1,**<br><br><br>**Rc**	1<br><br>0.01,<br>0.1<br><br>0.01	0.767<br><br>0.011<br>0,11<br><br>0.108	primary medium spiny striatal neuronal cultures (MSN) from the YAC128 HD mouse	Ginsenosides Rg5, Rb1 and Rc protected neurons from glutamate-induced apoptosis in vitro Huntington's disease (HD) assay.<br>Ginsenosides Rd, Re, Rg3, Rh1, Re, Rd, Rk1, Rh4 and Rk3 were inactive or exerted toxic effects in MSN cultures.
Choi et al., 2017	**Rg5**	26-52<br><br>Toxic in 78-104	20-40<br><br>Toxic in 60-80	hippocampal HT22 cells	Rg5 exhibits a neuroprotective effect in heat stress-induced apoptosis.<br>**Rg5 decreases** the production of thermal stress-induced **increase of NO,** which is an oxidative stress marker,<br>Rg5 **reduces** antioxidant enzymes **HO-1/Nrf2 and glutathione reductase** production and **AchE** activity in thermal stress-exposed HT22 cells.<br>Rg5 prevents thermal stress-induced inhibition of **CREB, BDNF, and GSK3b**, increasing their production in thermal stress-exposed HT22 cells.
Cho et al, 2015	**Rg5**	10<br>20	7.7<br>15.4	Human umbilical vein endothelial cells	Rg5 promotes angiogenesis and vasorelaxation by activating signal transduction pathways downstream of IGF-1R , including ERK, FAK, Akt/eNOS/NO, and Gi-mediated phospholipase C/Ca2/eNOS dimerization pathways. The vasodilative activity of Rg5 was mediated by the eNOS/NO/cGMP axis.
Yang et al, 2017	Rg5	0.1<br>1<br>10	-<br>0.767<br>7.67	Neonatal rat ventricular myocytes	Rg5 regulation of mitochondrial dysfunctions through **Akt** activation, and increased **ATP** production.
Kim et al., 2015	**Rg5**	25-100	19-96	breast cancer cells (MCF-7)	Rg5 **promotes breast cancer cell (MCF-7) apoptosis** by:<br>• increasing expression of p53, p21WAF1/CIP1 and p15INK4B<br>• decreasing expression of Cyclin D1, Cyclin E2, and CDK4
Liu et al., 2019	**Rg5**	100-200	77-154	human gastric cancer cells	Ginsenoside Rg5 induces G2/M phase arrest, apoptosis, and autophagy via **activating** ROS-mediated **MAPK** pathways in human gastric cancer

Song et al.,2021	**Rg5**	50	38	HeLa, A549 and 293T cancer cells	Rg5 inhibits cancer cell migration by **inhibiting the NF-kB** and erythropoietin-producing hepatocellular receptor A2 signaling pathways
Kim et al, 2019	**Rg5**	20<br>40	15.4<br>30.8	Human umbilical vein endothelial cells	Rg5 **suppressed** the production of TNF-α and IL-6 and the activation of NF-κB and ERK 1/2 by HMGB1-mediated septic responses.<br>Rg5 reduced HMGB1 release in LPS-activated HUVECs via activation the **SIRT1**-mediated deacetylation of HMGB1
Ahn et al, 2016	**Rg5**			RAW264.7 Macrophages and human epidermal keratinocytes - HaCaT cells	Rg5 suppresses NF-κB/p38 MAPK/STAT1 signaling
Park et al, 2015	**Rg5**			Pig Kidney EpithelialLLC-PK1 cells	Rg5 ameliorates renal cell damage by inhibiting inflammation and preventing apoptosis
Liang et al., 2015	**Rg5**	1.25-20	1-15	human cervical cancer cells	Rg5 induces apoptosis and DNA damage
Cheng et al., 2019	**Rg5**<br>**Rb1**<br>**Rg3**<br>**Rg1**<br>**Rh2**	131	100	PC12 cells	**Rg5 inhibits reactive oxygen species (ROS) and apoptosis**

CREB, cAMP-response element-binding protein; GR, gluthathione reductase; HO-1, heme oxygenase-1

Table S2. Doses, bioavailability and the maximal concentration of Ginsenosides in blood of human subjects and animals in pharmacokinetic studies of Ginseng

Reference	Gin-seno-sides	Conc. in blood	Conc. in blood	Bioavaila-bility	Human daily dose Ginseng dry prepara-tion	Human saily dose, Ginseno-sides	In vivo dose of dry preparation, mice/ rats/ dogs	Corres-ponding dose of Ginseng dry preparation in humans
**Units**		**nM**	**ng/ml**	**%**	**mg**	**mg**	**mg/kg**	**mg**
Xu et al., 2003	**Rb1**<br>**Rg1**	61447<br>9504	47130<br>7290	4.35<br>18.4			600	5800
Chen et al., 2016	**Rb1**<br>**Rc**<br>**Rd**<br>**Re**	104-195<br>13-26<br>13-26<br>4-6.5	80-150<br>10-20<br>10-20<br>3-5				454	14700
Ma et al., 2021	**Rg5**<br>**Rk1**			0.97<br>0.67				
Yoo et al., 2021	**Rg5**<br>**Rk1**<br>**Rg3**<br>**Ck**<br>**Rb1**<br>**Rh2**	2-10<br>1-4<br>2-10<br>15<br>2-4<br>2-22	1.4-7.6<br>0.7-2.8<br>1.6-7.3<br>11.7<br>1.8-2.8<br>1.6-16.9		**9000**	7-73<br>1-33<br>5-39<br>-<br>18-49<br>-		
Zhou et al., 2017	**Rg5**<br>**Rk1**<br>**Rg3**<br>**Ck**<br>**Rb1**<br>**Rh2**<br>**Rh3**	80.8<br>48.2<br>100.4<br>48.2<br>275.1<br>328.6<br>35.2	62<br>37<br>77<br>37<br>211<br>252<br>27				4000	39000
Kim et al., 2013	**Rb1**<br>**Ck**	5.1<br>11.0	3.9<br>8.4		9000			
Kim et al., 2018	**Rb1**<br>**Rg3**<br>**Ck**<br>**Rh2**<br>**Gin seng**			4.3<br>2.63<br>35.0<br>5,4, 6			4.3<br>2.63<br>35.0<br>5,4, 6<br>1000	9730
Won et al., 2019	**Rb1**<br>**Rg1**<br>**Rh2**<br>**Ginseng**			4.35<br>2.63<br>6.4			4.35<br>2.63<br>6.4<br>1000	9730

Table S4. The pharmacological activity of Ginsenoside Rg5 in *in vivo* experiments

First author, year	Experimental animals	Dose of Rg5, mg/kg	Corresponding human dose of Red Ginseng dry root, mg /BW	Disease model	Effect
Cheng et al., 2019	Male Sprague-Dawley rats	10	5000	Neurodegenerative Disorders	Rg5 protects the brain against ischemic injury *in vivo,* **reducing** cerebral ischemic injury, cerebral infarction volume, brain neurological dysfunction of ischemia-reperfusion.<br>The neuroprotective mechanism is associated with **inhibition of** the expression of Toll-like receptor 4 (**TLR4),** nuclear transcription factor P65 (**NF-kB**), the expression of interleukines **IL-1β, IL-6 and TNF-**α and silencing information regulator (**SIRT1**) in the hippocar region of rats
Chu et al, 2014	Mail Wistar rats	5<br>10<br>20	2500<br>5000<br>10000	Neurodegenerative Disorders	Rg5 improved cognitive dysfunction and attenuated neuroinflammatory responses in streptozotocin (STZ)-induced memory impaired rats.<br>Ginsenoside Rg5:<br>• **decreased** levels of cytokines TNF-α and IL-1β and acetylcholinesterase (**AChE**) activity, but<br>• **enhanced** choline acetyltransferase (**ChAT**) activity, the expressions of insulin-like growth factors 1 (**IGF-**1) brain derived neurotrophic factor **(BDNF**) and Aβ deposition in the hippocampus and cerebral cortex of STZ rats.
Kim et al, 2013	Male ICR mice	10	2500	Neurodegenerative Disorders	Ginsenoside Rg5 protects memory deficit by **inhibiting AChE activity** and **increasing BDNF expression** and **CREB activation**
Shao et al, 2018	Male Kunming mice			Mood and behavioral disorders Depression	Ginsenoside Rg5 can exert sedative and hypnotic effects by affecting the GABA nervous system and the serotonin nervous system.<br>Rg5 reduce the locomotor activity of mice and promote the sleep quality index, the sleep latency and prolong the sleep time of mice. Furthermore, Rg5 augmented the GABA/Glu ratio, up-regulating the expression of the GABAA receptor and the GABAB receptor.
Yang et al, 2017	Male ICR mice	50	12000	Metabolic disorders Cardioprotection	Ginsenoside Rg5 increases cardiomyocyte resistance to ischemic injury

Xiao et al, 2017	Male C57BL/6J mice	50	12000	Metabolic disorders Diabetes	Ginsenoside Rg5 Inhibits Succinate-induced Lipolysis in Adipose Tissue and Prevents Muscle Insulin Resistance.<br>Rg5 treatment **reduced** cellular energy charge, suppressed Endoplasmatic Reticulum stress, , inflammation and cAMP/PKA activation, contributing to lipolysis and insulin resistance.<br>Ginsenoside Rg5 treatment suppressed NLRP3 inflammasome activation, preserved PDE3B expression and then reduced cAMP accumulation, contributing to inhibition of lipolysis.
Zhu et al, 2020	C57BL/6 diabetic mice	30<br>60	7000<br>14000	Metabolic disorders Diabetes	Rg5 attenuated renal injury in diabetic mice by inhibiting oxidative stress and NLRP3 inflammasome activation to reduce inflammatory responses<br>including ROS production, oxidative stress markers (MDA, SOD, and GSH-PX), Nox4 and the expression levels of the NLRP3 inflammasome (NLRP3, ASC, and Caspase-1), the inflammatory cytokines IL-1β and IL-18, the expression of NF-kB and the phosphorylation of p38 MAPK in kidney
Kim et al, 2019	Male C57BL/6 mice	0.061<br>1.1 μM	15	Inflammation	Rg5 inhibited HMGB1-mediated hyperpermeability, leukocyte migration, the sepsis-related mortality, and tissue injury in mice.

References

An, X., Fu, R., Ma, P., Ma, X., & Fan, D. Ginsenoside Rk1 inhibits cell proliferation and promotes apoptosis in lung squamous cell carcinoma by calcium signaling pathway. RSC advances, 2019, 9, 25107-25118. doi: 10.1039/c9ra05037j

Chen S, Xu HQ, Zhang J, Wang CX, Liu JQ, Peng LH, Cheng JL, Liu A. A systematic study of the dissolution and relative bioavailability of four ginsenosides in the form of ultrafine granular powder, common powder and traditional pieces of Panax quinquefolius L, in vitro and in beagles. J Ethnopharmacol. 2016 Jun 5;185:9-16. doi: 10.1016/j.jep.2016.03.032. Epub 2016 Mar 11. PMID: 26976765.

Cheng Z, Zhang M, Ling C, Zhu Y, Ren H, Hong C, Qin J, Liu T, Wang J. Neuroprotective Effects of Ginsenosides against Cerebral Ischemia. Molecules. 2019 Mar 20;24(6):1102. doi: 10.3390/molecules24061102. PMID: 30897756; PMCID: PMC6471240.

Choi SY, Kim KJ, Song JH, Lee BY. Ginsenoside Rg5 prevents apoptosis by modulating heme-oxygenase-1/nuclear factor E2-related factor 2 signaling and alters the expression of cognitive impairment-associated genes in thermal stress-exposed HT22 cells. J Ginseng Res. 2018 Apr;42(2):225-228. doi: 10.1016/j.jgr.2017.02.002. Epub 2017 Feb 28. PMID: 29719470; PMCID: PMC5926500.

Hong Y, Fan D. Ginsenoside Rk1 induces cell cycle arrest and apoptosis in MDA-MB-231 triple negative breast cancer cells. Toxicology. 2019 Apr 15;418:22-31. doi: 10.1016/j.tox.2019.02.010. Epub 2019 Feb 21. PMID: 30797898.

Hong Y, Fan D. Ginsenoside Rk1 induces cell death through ROS-mediated PTEN/PI3K/Akt/mTOR signaling pathway in MCF-7 cells, Journal of Functional Foods. 2019; 57: 255-265. https://doi.org/10.1016/j.jff.2019.04.019.

Kim H, Lee JH, Kim JE, Kim YS, Ryu CH, Lee HJ, Kim HM, Jeon H, Won HJ, Lee JY, Lee J. Micro-/nano-sized delivery systems of ginsenosides for improved systemic bioavailability. J Ginseng Res. 2018 Jul;42(3):361-369. doi: 10.1016/j.jgr.2017.12.003. Epub 2018 Jan 9. PMID: 29983618; PMCID: PMC6026383.

Kim HK. Pharmacokinetics of ginsenoside Rb1 and its metabolite compound K after oral administration of Korean Red Ginseng extract. J Ginseng Res. 2013 Oct;37(4):451-6. doi: 10.5142/jgr.2013.37.451. PMID: 24235859; PMCID: PMC3825860.

Kim SJ, Kim AK. Anti-breast cancer activity of Fine Black ginseng (Panax ginseng Meyer) and ginsenoside Rg5. J Ginseng Res. 2015 Apr;39(2):125-34. doi: 10.1016/j.jgr.2014.09.003. Epub 2014 Oct 18. PMID: 26045685; PMCID: PMC4452536.

Liang LD, He T, Du TW, Fan YG, Chen DS, Wang Y. Ginsenoside-Rg5 induces apoptosis and DNA damage in human cervical cancer cells. Mol Med Rep. 2015 Feb;11(2):940-6. doi: 10.3892/mmr.2014.2821. Epub 2014 Oct 30. PMID: 25355274; PMCID: PMC4262516.

Liu Y , Fan D . Ginsenoside Rg5 induces apoptosis and autophagy via the inhibition of the PI3K/Akt pathway against breast cancer in a mouse model. Food Funct. 2018 Nov 14;9(11):5513-5527. doi: 10.1039/c8fo01122b. PMID: 30207362.

Liu Y, Fan D. Ginsenoside Rg5 induces G2/M phase arrest, apoptosis and autophagy via regulating ROS-mediated MAPK pathways against human gastric cancer. Biochem Pharmacol. 2019 Oct;168:285-304. doi: 10.1016/j.bcp.2019.07.008. Epub 2019 Jul 10. PMID: 31301277.

Ma C, Lin Q, Xue Y, Ju Z, Deng G, Liu W, Sun Y, Guan H, Cheng X, Wang C. Pharmacokinetic studies of ginsenosides Rk1 and Rg5 in rats by UFLC-MS/MS. Biomed Chromatogr. 2021 Mar 2:e5108. doi: 10.1002/bmc.5108. Epub ahead of print. PMID: 33650162.

Pan W, Xue B, Yang C, Miao L, Zhou L, Chen Q, Cai Q, Liu Y, Liu D, He H, Zhang Y, Yin T, Tang X. Biopharmaceutical characters and bioavailability improving strategies of ginsenosides. Fitoterapia. 2018 Sep;129:272-282. doi: 10.1016/j.fitote.2018.06.001. Epub 2018 Jun 5. PMID: 29883635.

Sharma A, Lee HJ. Ginsenoside Compound K: Insights into Recent Studies on Pharmacokinetics and Health-Promoting Activities. Biomolecules. 2020 Jul 10;10(7):1028. doi: 10.3390/biom10071028. PMID: 32664389; PMCID: PMC7407392.

Song L, Yang F, Wang Z, Yang L, Zhou Y. Ginsenoside Rg5 inhibits cancer cell migration by inhibiting the nuclear factor-κB and erythropoietin-producing hepatocellular receptor A2 signaling pathways. Oncol Lett. 2021 Jun;21(6):452. doi: 10.3892/ol.2021.12713. Epub 2021 Apr 8. PMID: 33907562; PMCID: PMC8063271.

Won HJ, Kim HI, Park T, Kim H, Jo K, Jeon H, Ha SJ, Hyun JM, Jeong A, Kim JS, Park YJ, Eo YH, Lee J. Non-clinical pharmacokinetic behavior of ginsenosides. J Ginseng Res. 2019 Jul;43(3):354-360. doi: 10.1016/j.jgr.2018.06.001. Epub 2018 Jun 18. PMID: 31308806; PMCID: PMC6606970.

Xu D, Wang C, Zhao W, Gao S, Cui Z. Antidepressant-like effects of ginsenoside Rg5 in mice: Involving of hippocampus BDNF signaling pathway. Neurosci Lett. 2017 Apr 3;645:97-105. doi: 10.1016/j.neulet.2017.02.071. Epub 2017 Mar 1. PMID: 28257788.

Xu QF, Fang XL, Chen DF. Pharmacokinetics and bioavailability of ginsenoside Rb1 and Rg1 from Panax notoginseng in rats. J Ethnopharmacol. 2003 Feb;84(2-3):187-92. doi: 10.1016/s0378-8741(02)00317-3. PMID: 12648814.

Yoo S, Park BI, Kim DH, Lee S, Lee SH, Shim WS, Seo YK, Kang K, Lee KT, Yim SV, Soung DY, Kim BH. Ginsenoside Absorption Rate and Extent Enhancement of Black Ginseng (CJ EnerG) over Red Ginseng in Healthy Adults. Pharmaceutics. 2021 Apr 2;13(4):487. doi: 10.3390/pharmaceutics13040487. PMID: 33918329; PMCID: PMC8067055.

Zhou QL, Zhu DN, Yang YF, Xu W, Yang XW. Simultaneous quantification of twenty-one ginsenosides and their three aglycones in rat plasma by a developed UFLC-MS/MS assay: Application to a pharmacokinetic study of red Ginseng. J Pharm Biomed Anal. 2017 Apr 15;137:1-12. doi: 10.1016/j.jpba.2017.01.009. Epub 2017 Jan 6. PMID: 28086165.

Zhu Y, Zhu C, Yang H, Deng J, Fan D. Protective effect of ginsenoside Rg5 against kidney injury via inhibition of NLRP3 inflammasome activation and the MAPK signaling pathway in high-fat diet/streptozotocin-induced diabetic mice. Pharmacol Res. 2020 May;155:104746. doi: 10.1016/j.phrs.2020.104746. Epub 2020 Mar 7. PMID: 32156650.
