# Supplementary material for: Network Pharmacology of Red Ginseng (Part I): Effects of Ginsenoside Rg5 at Physiological and Sub-Physiological Concentrations
Source: Pharmaceuticals (Basel). 2021 Sep 29;14(10):999. doi: 10.3390/ph14100999 (PMC8537973; doi:10.3390/ph14100999)
Supplement: Supplementary file 1 [file pharmaceuticals-14-00999-s001.zip › Suppl 5 SIRT signaling S2-S8.pdf]

Sirtuins are class III histone deacetylase enzymes that use NAD+ as a co-substrate for their enzymatic activities.
In mammals, there are 7 sirtuin members (SIRT1-7), which play important roles in aging, metabolism, cancer, inflammation,
DNA repair and cellular responses to stress.

100 µM

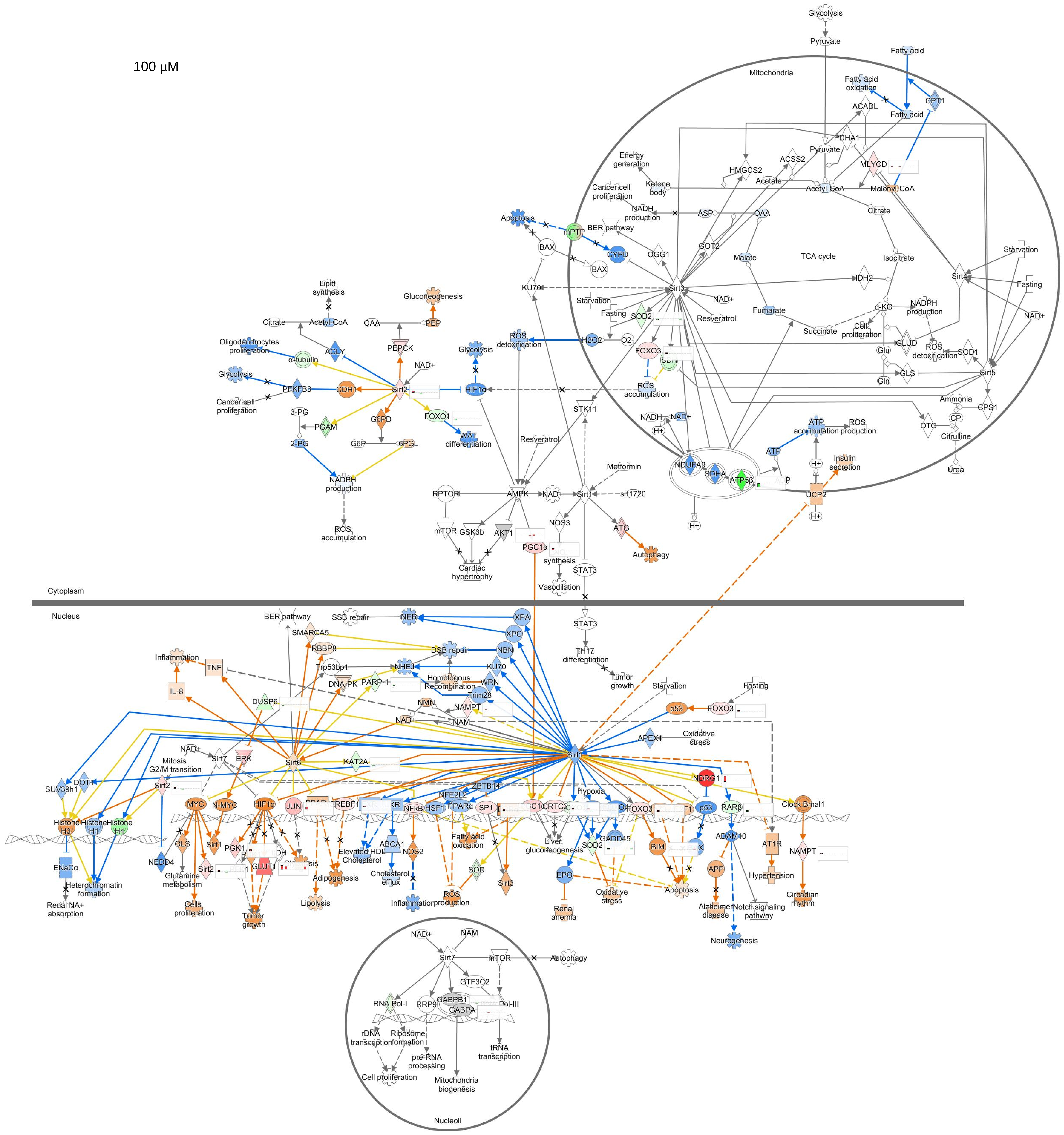

© 2000-2021 QIAGEN. All rights reserved.

Sirtuins are class III histone deacetylase enzymes that use NAD+ as a co-substrate for their enzymatic activities.
In mammals, there are 7 sirtuin members (SIRT1-7), which play important roles in aging, metabolism, cancer, inflammation,
DNA repair and cellular responses to stress.

1 µM

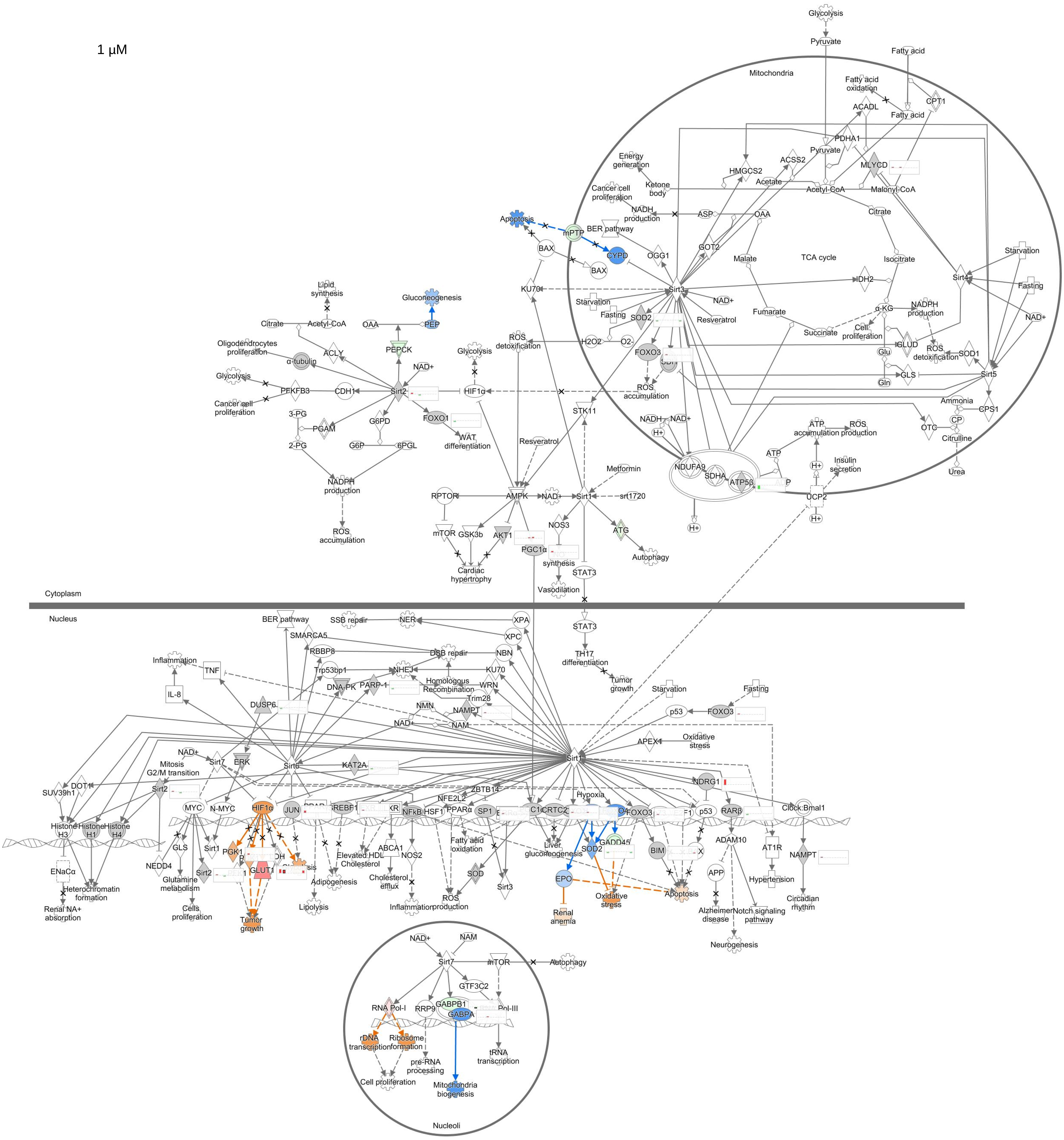

© 2000-2021 QIAGEN. All rights reserved.

Sirtuins are class III histone deacetylase enzymes that use NAD+ as a co-substrate for their enzymatic activities.
In mammals, there are 7 sirtuin members (SIRT1-7), which play important roles in aging, metabolism, cancer, inflammation,
DNA repair and cellular responses to stress.

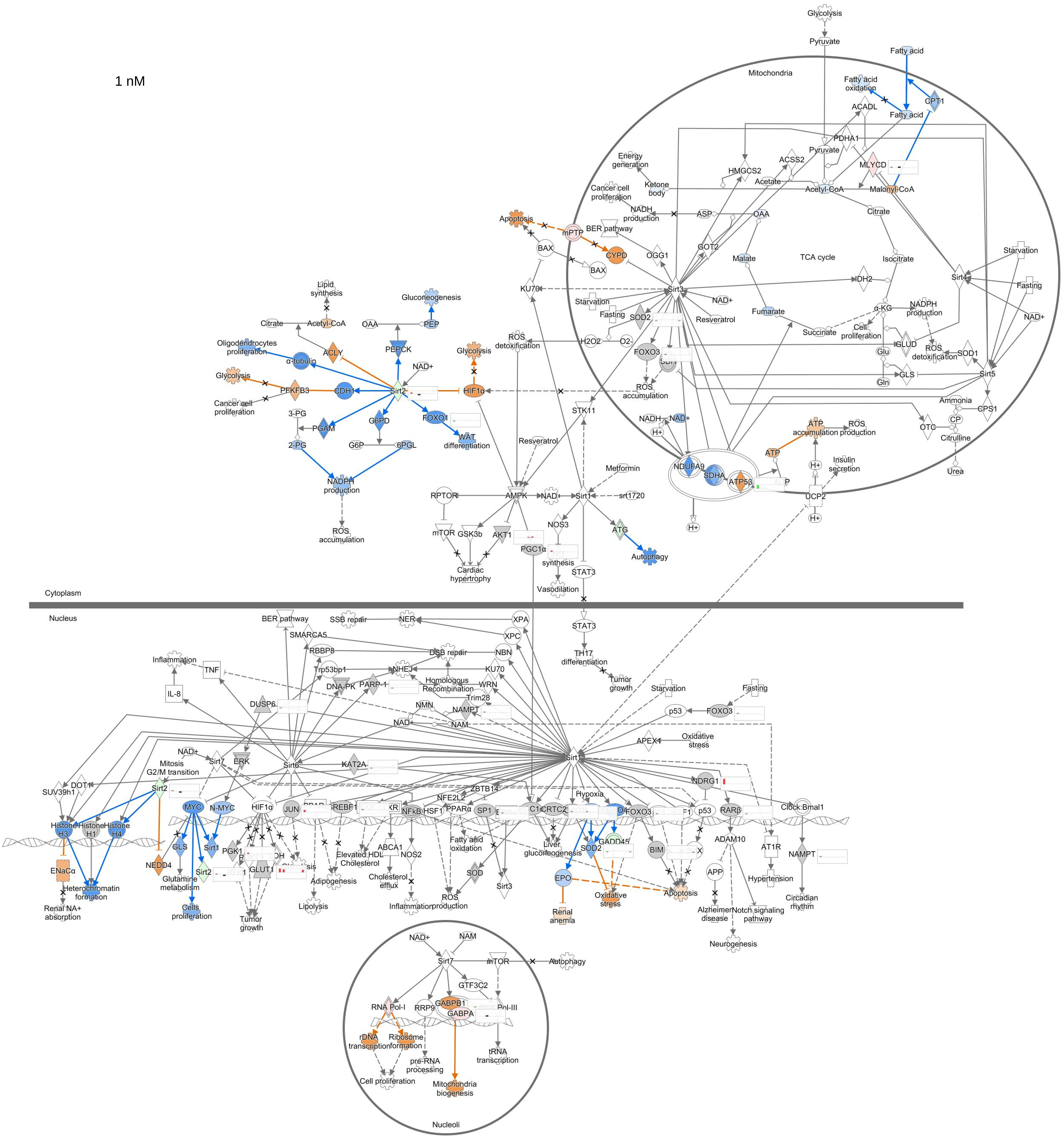

© 2000-2021 QIAGEN. All rights reserved.

Sirtuins are class III histone deacetylase enzymes that use NAD+ as a co-substrate for their enzymatic activities.
In mammals, there are 7 sirtuin members (SIRT1-7), which play important roles in aging, metabolism, cancer, inflammation,
DNA repair and cellular responses to stress.

1 pM

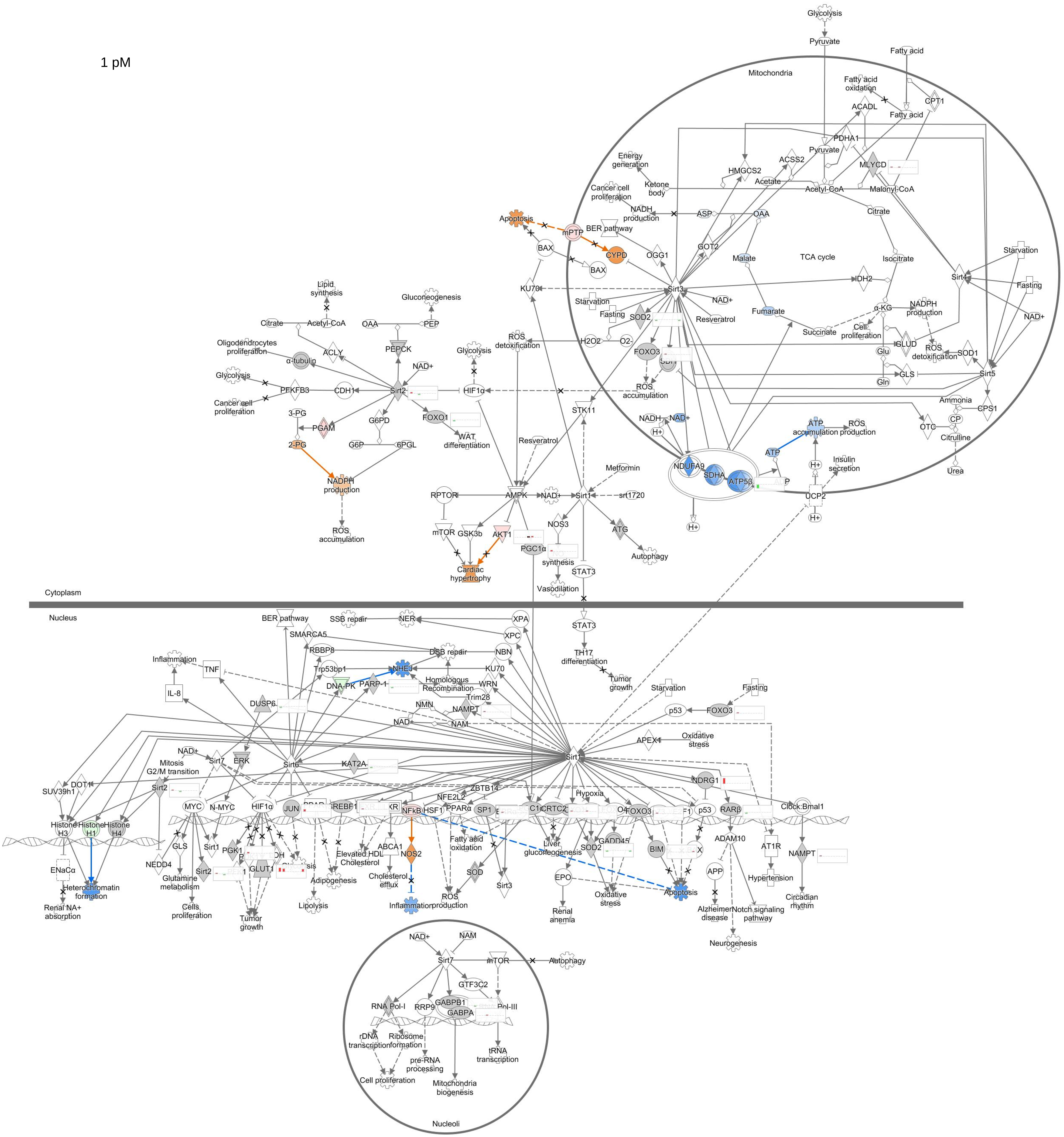

© 2000-2021 QIAGEN. All rights reserved.

Sirtuins are class III histone deacetylase enzymes that use NAD+ as a co-substrate for their enzymatic activities.
In mammals, there are 7 sirtuin members (SIRT1-7), which play important roles in aging, metabolism, cancer, inflammation,
DNA repair and cellular responses to stress.

1 fM

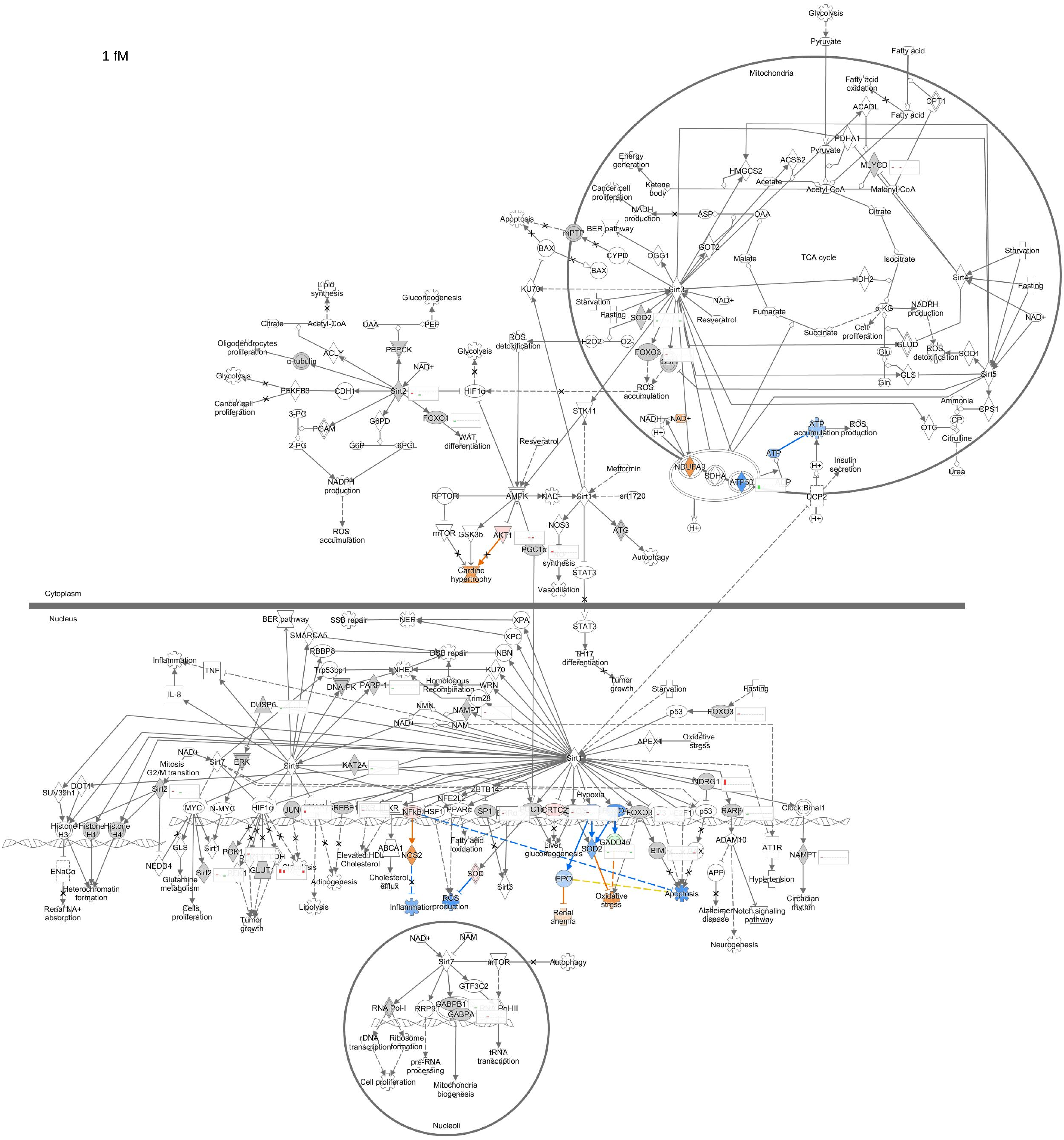

© 2000-2021 QIAGEN. All rights reserved.

Sirtuins are class III histone deacetylase enzymes that use NAD+ as a co-substrate for their enzymatic activities.
In mammals, there are 7 sirtuin members (SIRT1-7), which play important roles in aging, metabolism, cancer, inflammation,
DNA repair and cellular responses to stress.

10 aM

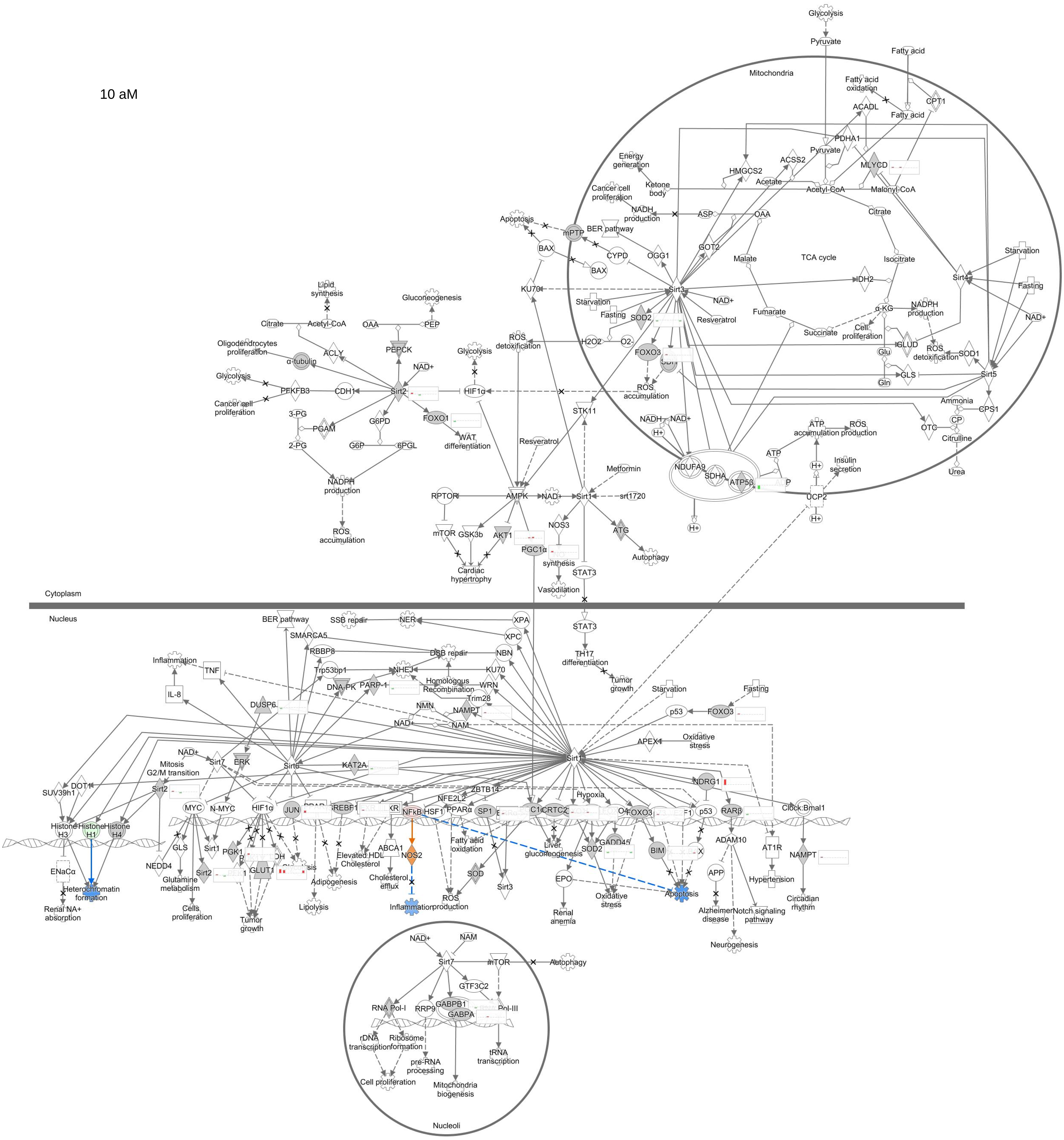

© 2000-2021 QIAGEN. All rights reserved.

Sirtuins are class III histone deacetylase enzymes that use NAD+ as a co-substrate for their enzymatic activities.
In mammals, there are 7 sirtuin members (SIRT1-7), which play important roles in aging, metabolism, cancer, inflammation,
DNA repair and cellular responses to stress.

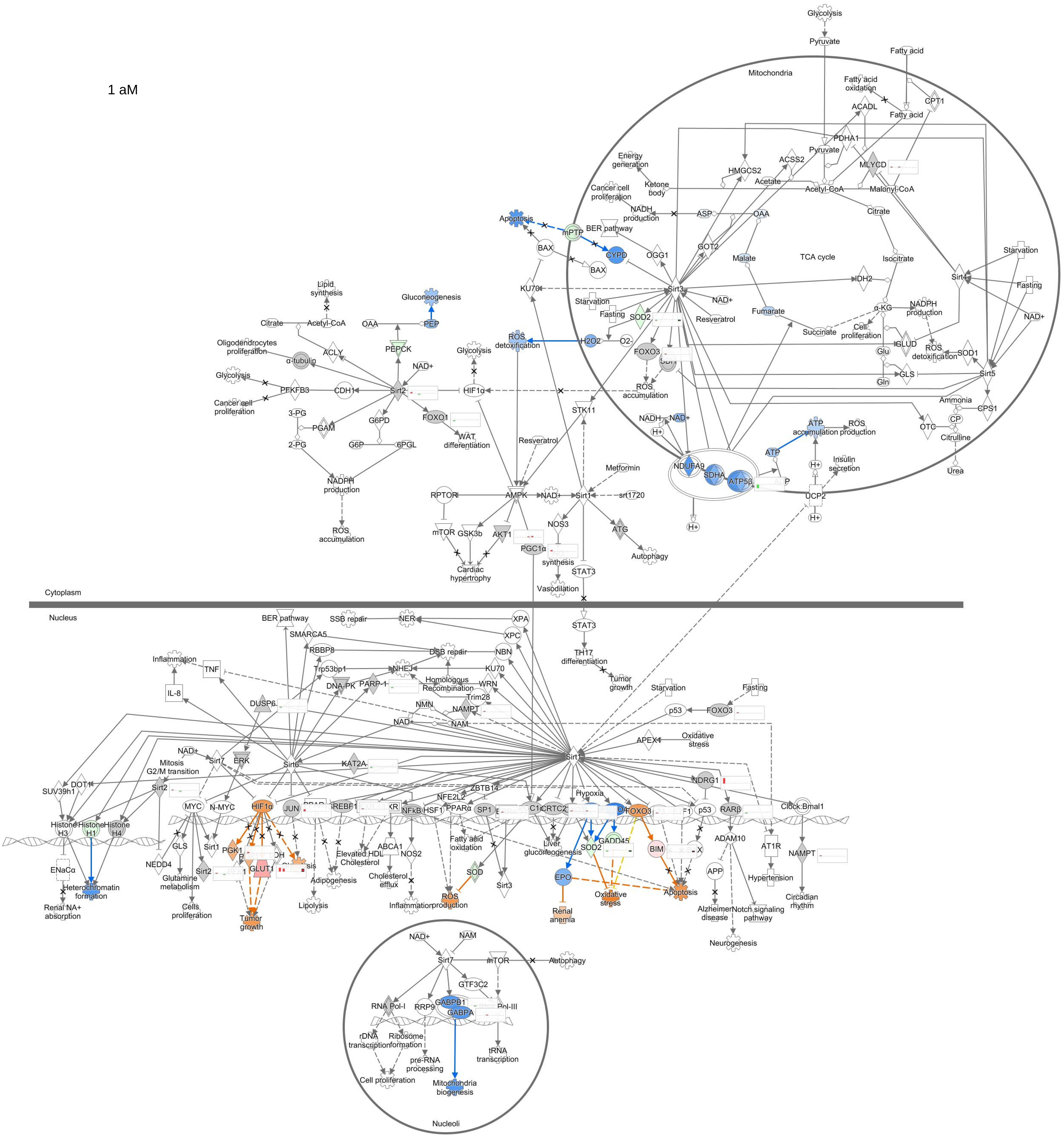

© 2000-2021 QIAGEN. All rights reserved.
