# Supplementary material for: Network Pharmacology of Red Ginseng (Part I): Effects of Ginsenoside Rg5 at Physiological and Sub-Physiological Concentrations
Source: Pharmaceuticals (Basel). 2021 Sep 29;14(10):999. doi: 10.3390/ph14100999 (PMC8537973; doi:10.3390/ph14100999)
Supplement: Supplementary file 1 [file pharmaceuticals-14-00999-s001.zip › Suppl 11 PD immunotherapy pathways.pdf]

PD-1, PD-L1 cancer immunotherapy pathway

100 µM

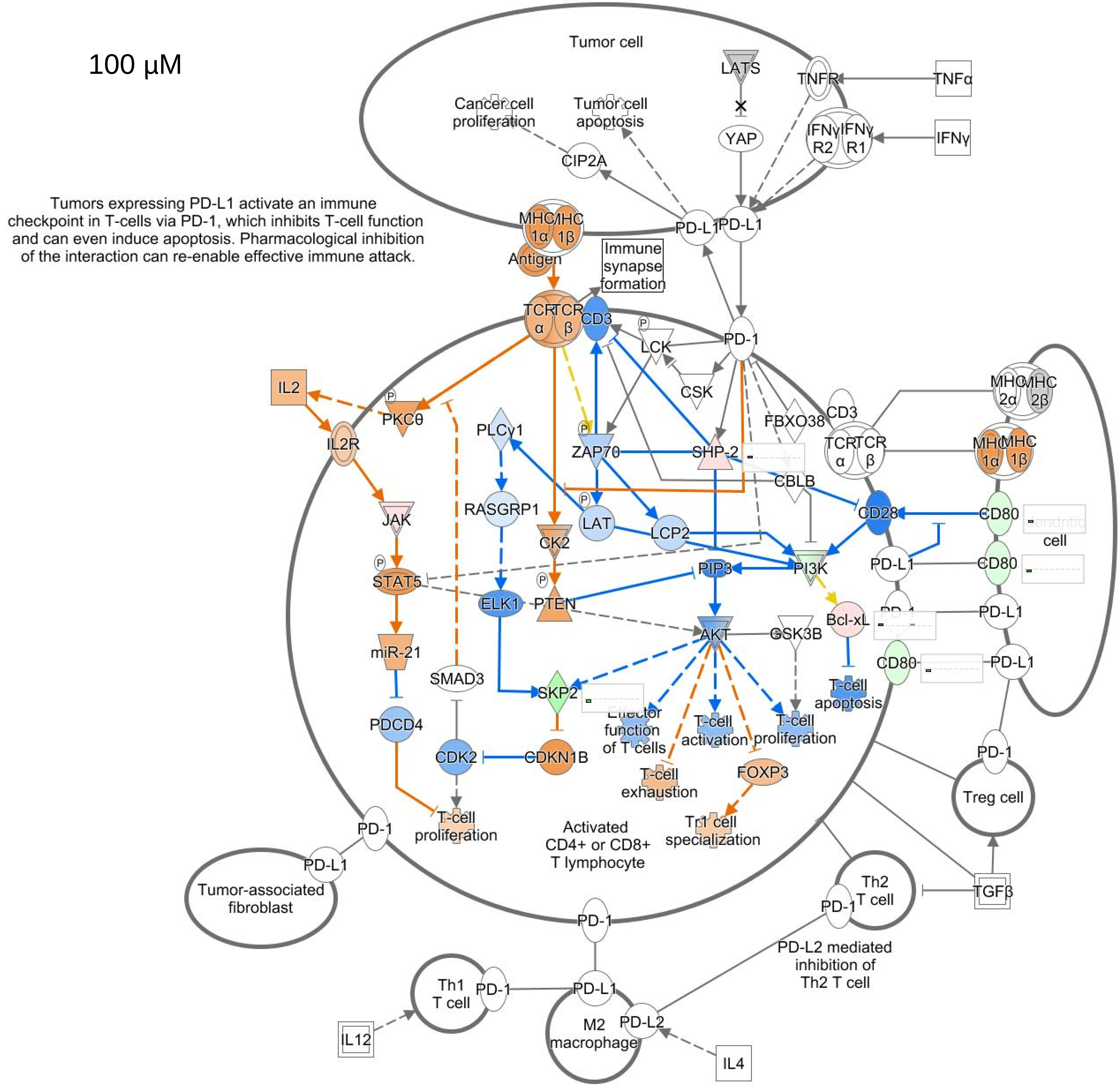

© 2000-2021 QIAGEN. All rights reserved.

PD-1, PD-L1 cancer immunotherapy pathway

1 µM

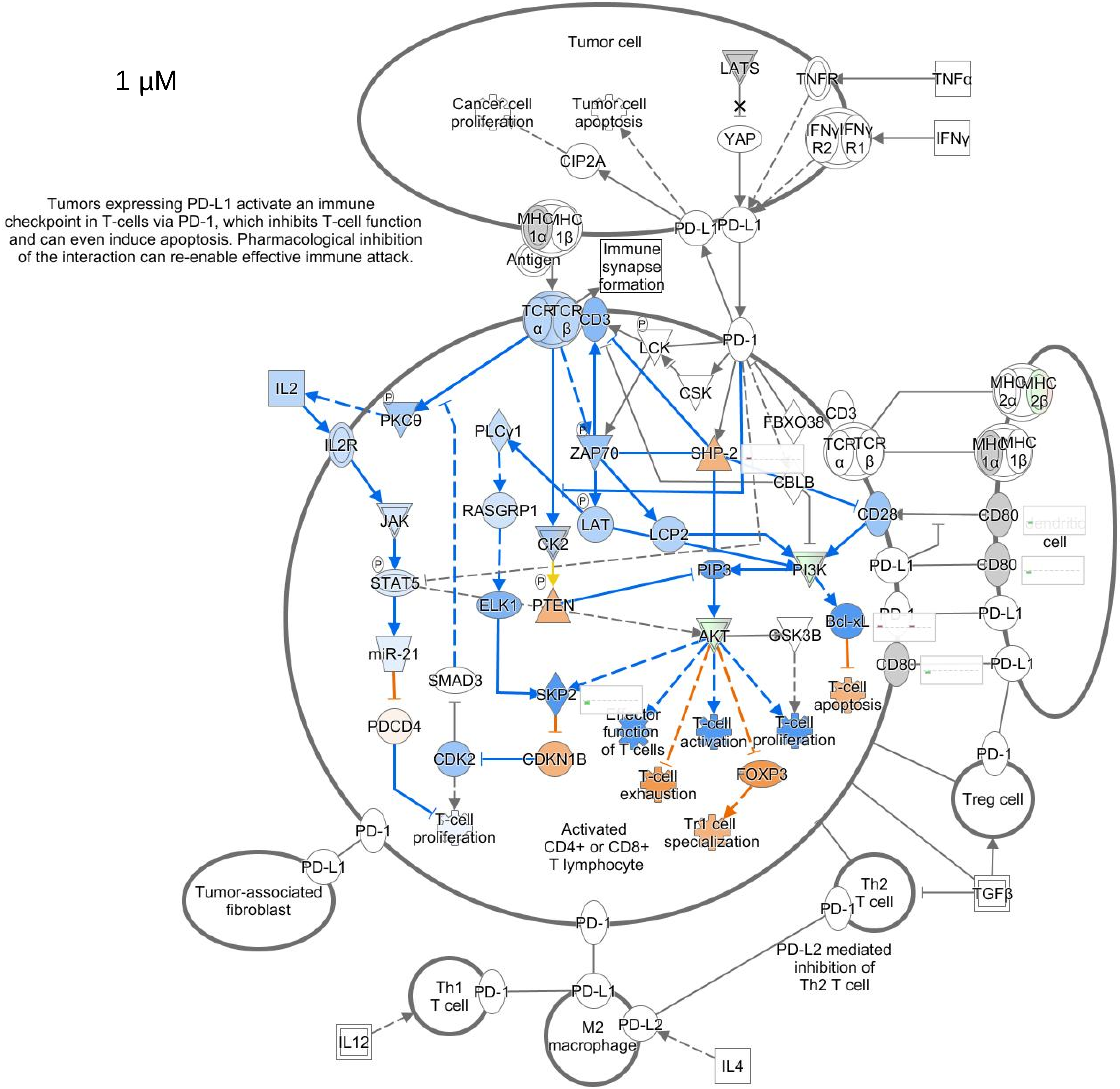

© 2000-2021 QIAGEN. All rights reserved.

1 nM

Tumors expressing PD-L1 activate an immune checkpoint in T-cells via PD-1, which inhibits T-cell function and can even induce apoptosis. Pharmacological inhibition of the interaction can re-enable effective immune attack.

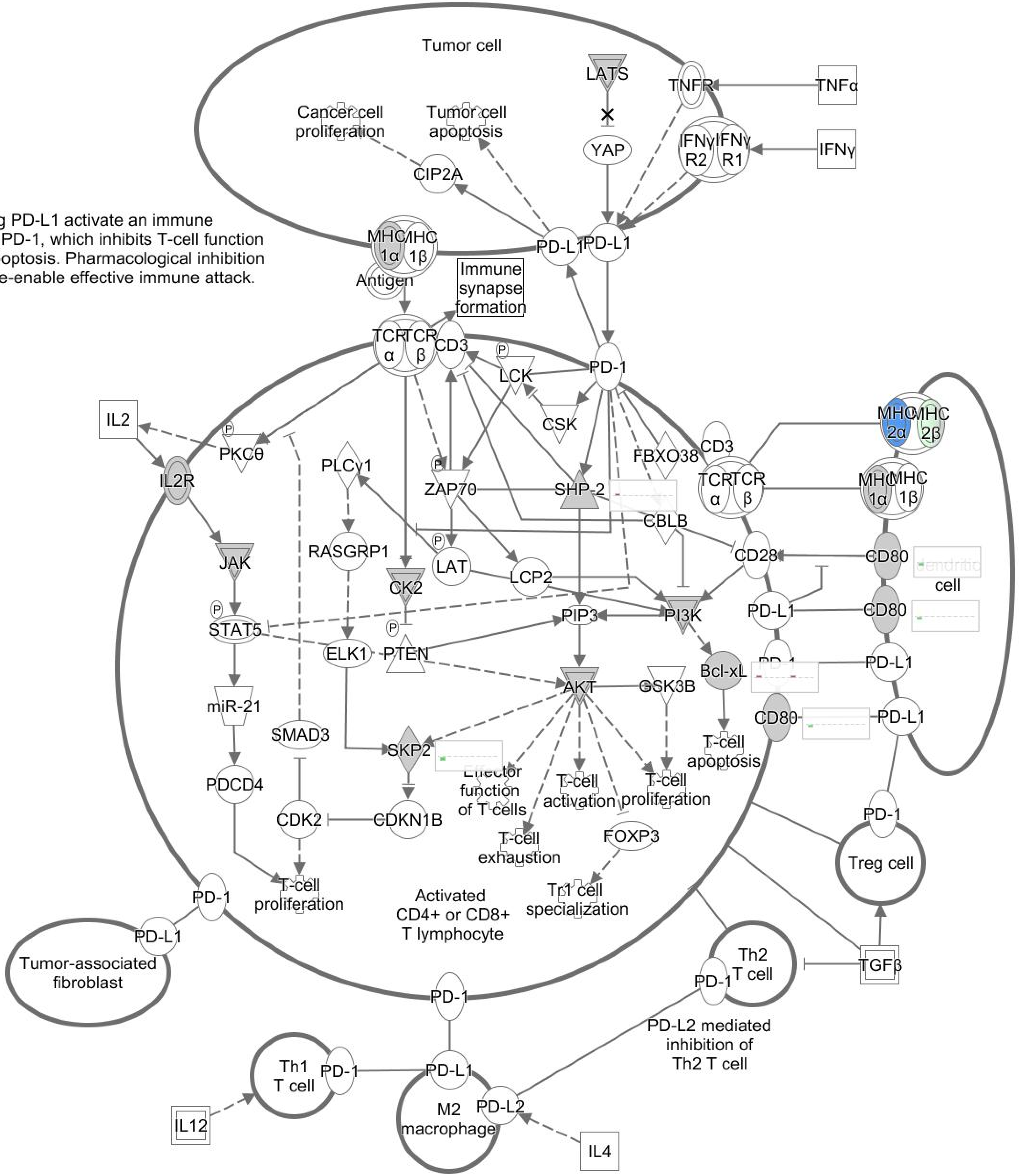

© 2000-2021 QIAGEN. All rights reserved.

1 pM

Tumors expressing PD-L1 activate an immune checkpoint in T-cells via PD-1, which inhibits T-cell function and can even induce apoptosis. Pharmacological inhibition of the interaction can re-enable effective immune attack.

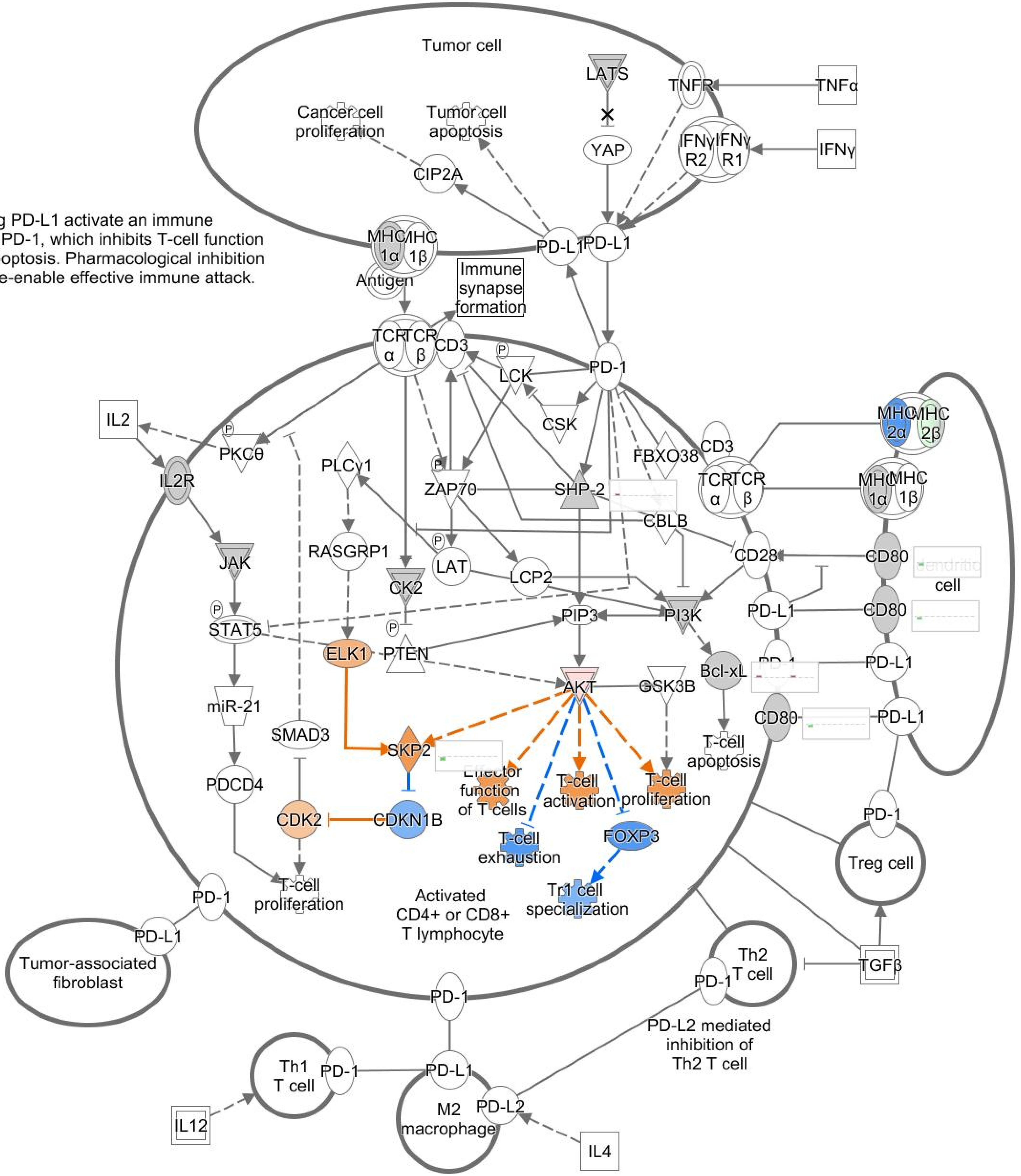

© 2000-2021 QIAGEN. All rights reserved.

PD-1, PD-L1 cancer immunotherapy pathway

1 fM

Tumors expressing PD-L1 activate an immune checkpoint in T-cells via PD-1, which inhibits T-cell function and can even induce apoptosis. Pharmacological inhibition of the interaction can re-enable effective immune attack.

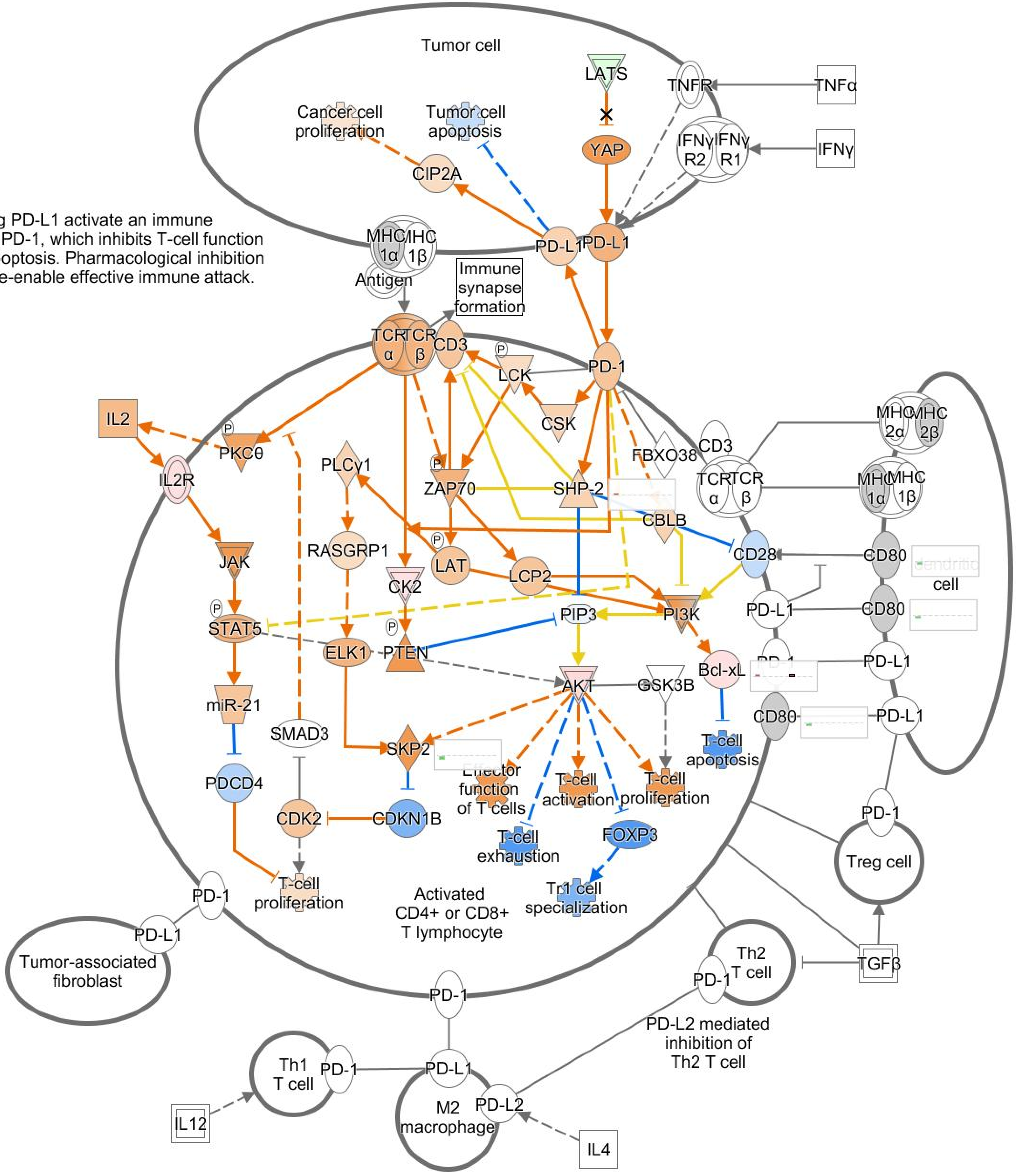

© 2000-2021 QIAGEN. All rights reserved.

10 aM

Tumors expressing PD-L1 activate an immune checkpoint in T-cells via PD-1, which inhibits T-cell function and can even induce apoptosis. Pharmacological inhibition of the interaction can re-enable effective immune attack.

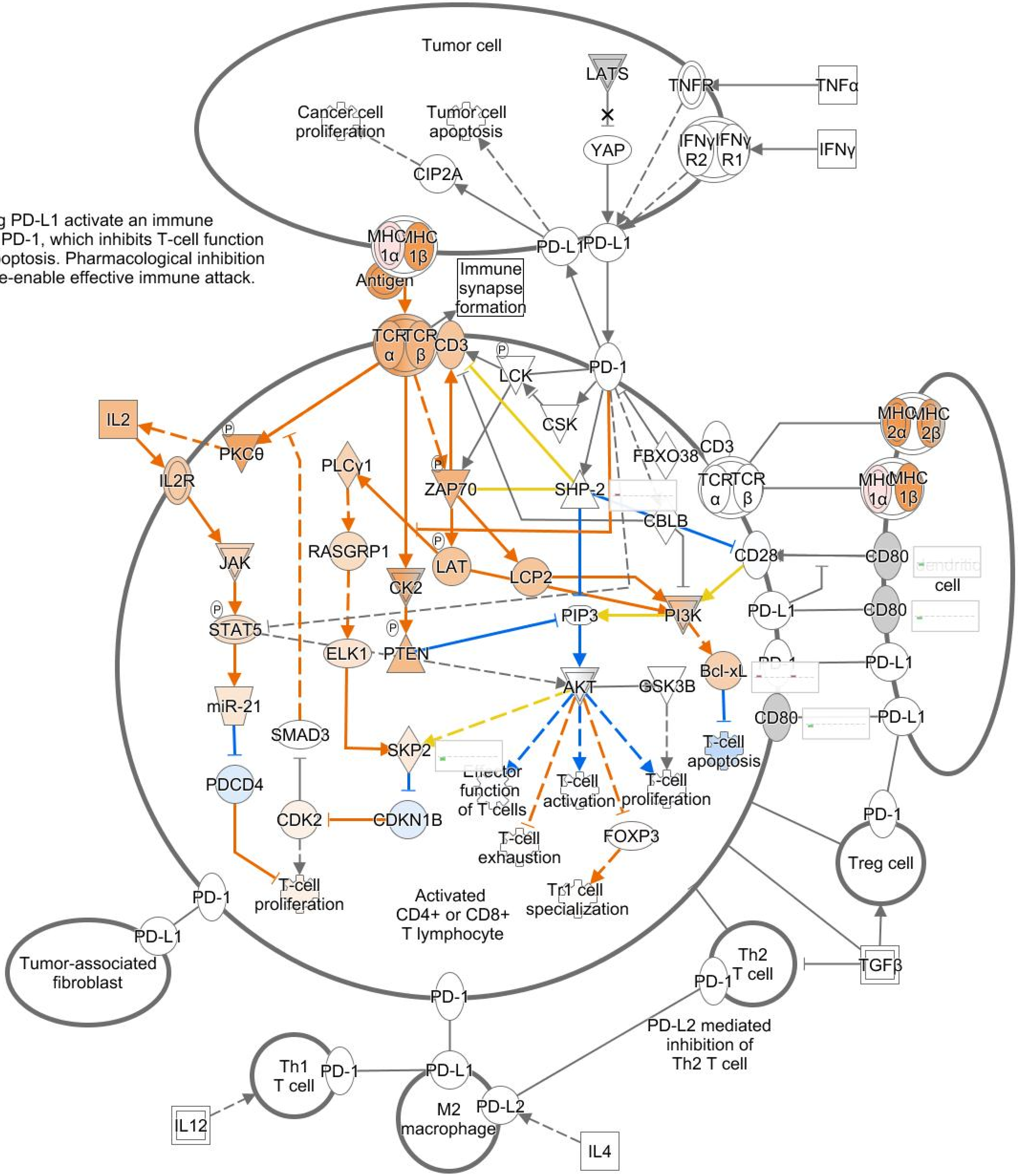

© 2000-2021 QIAGEN. All rights reserved.

1 aM

Tumors expressing PD-L1 activate an immune checkpoint in T-cells via PD-1, which inhibits T-cell function and can even induce apoptosis. Pharmacological inhibition of the interaction can re-enable effective immune attack.

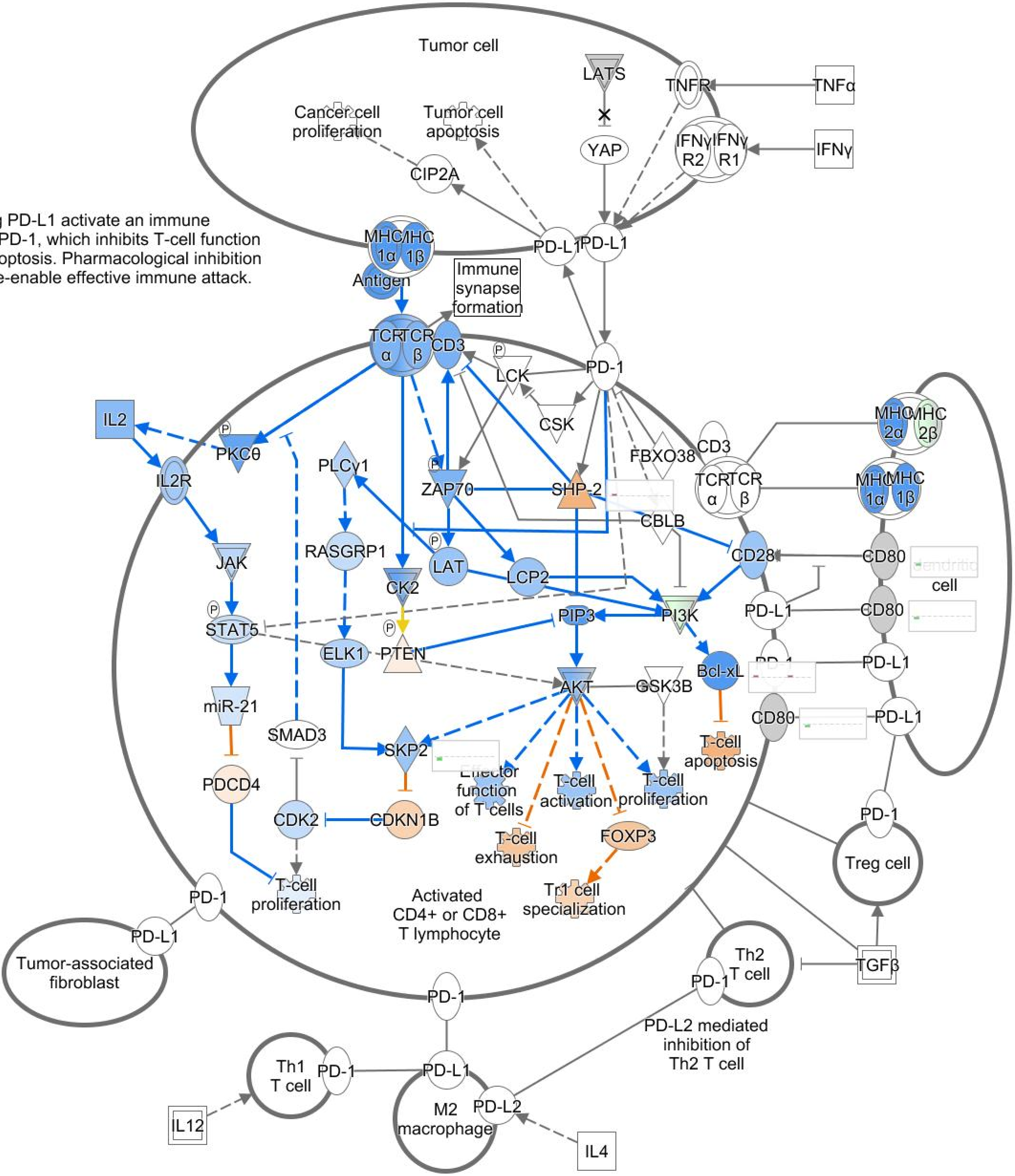

© 2000-2021 QIAGEN. All rights reserved.
