# Supplementary material for: Network Pharmacology of Red Ginseng (Part I): Effects of Ginsenoside Rg5 at Physiological and Sub-Physiological Concentrations
Source: Pharmaceuticals (Basel). 2021 Sep 29;14(10):999. doi: 10.3390/ph14100999 (PMC8537973; doi:10.3390/ph14100999)
Supplement: Supplementary file 1 [file pharmaceuticals-14-00999-s001.zip › Suppl 3 Neuroinflammation signaling.pdf]

Neuroinflammation involves numerous cell types, acts to clear neuronal damage, and plays a key role in maintaining the homeostasis of CNS. Homeostasis can be lost through various regulatory failures, or when humoral immune components cross the blood-brain barrier, causing chronic inflammation with excessive cell and tissue damage, which is associated with neurodegenerative diseases.

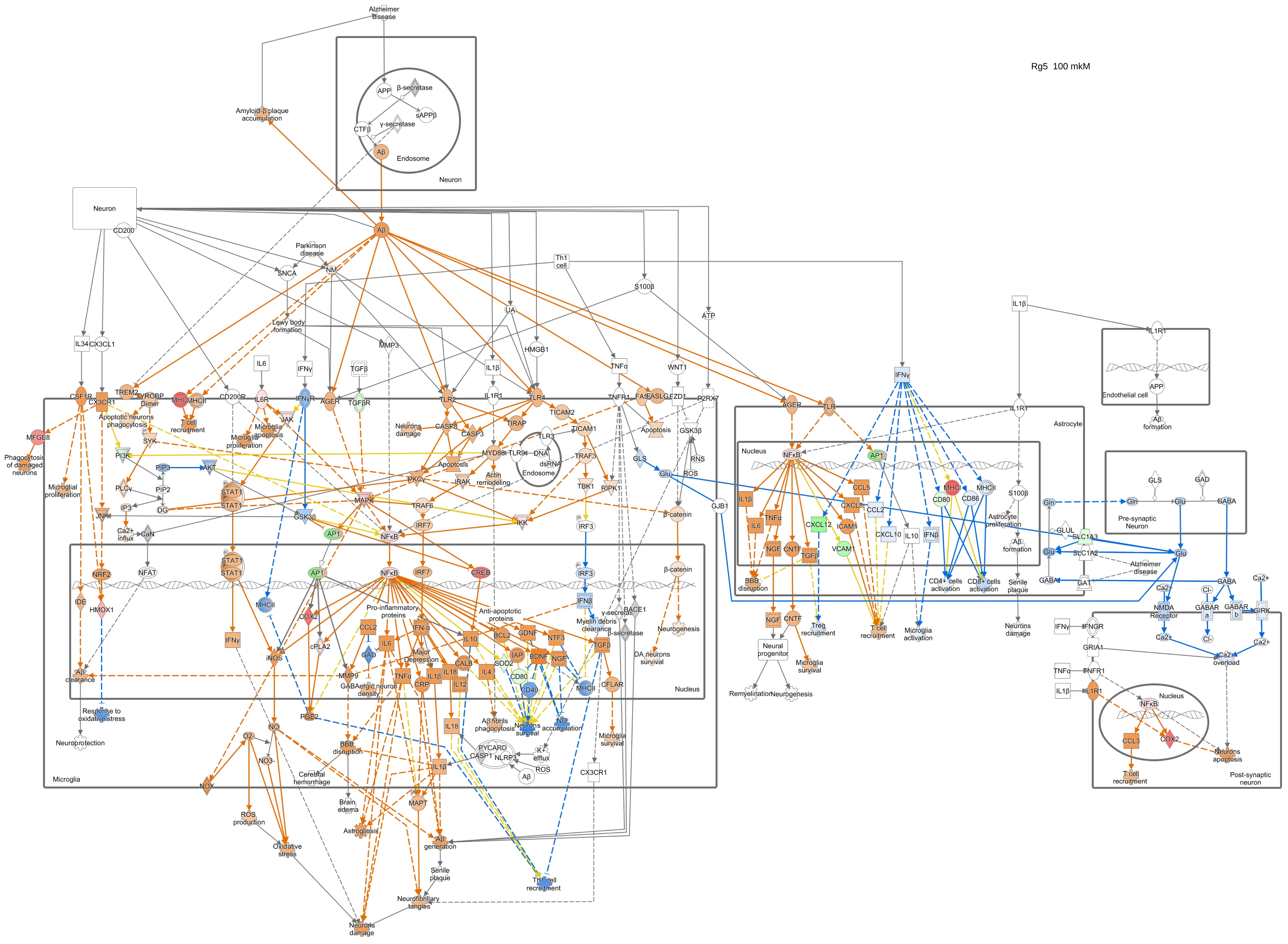

© 2000-2021 QIAGEN. All rights reserved.

Neuroinflammation involves numerous cell types, acts to clear neuronal damage, and plays a key role in maintaining the homeostasis of CNS. Homeostasis can be lost through various regulatory failures, or when humoral immune components cross the blood-brain barrier, causing chronic inflammation with excessive cell and tissue damage, which is associated with neurodegenerative diseases.

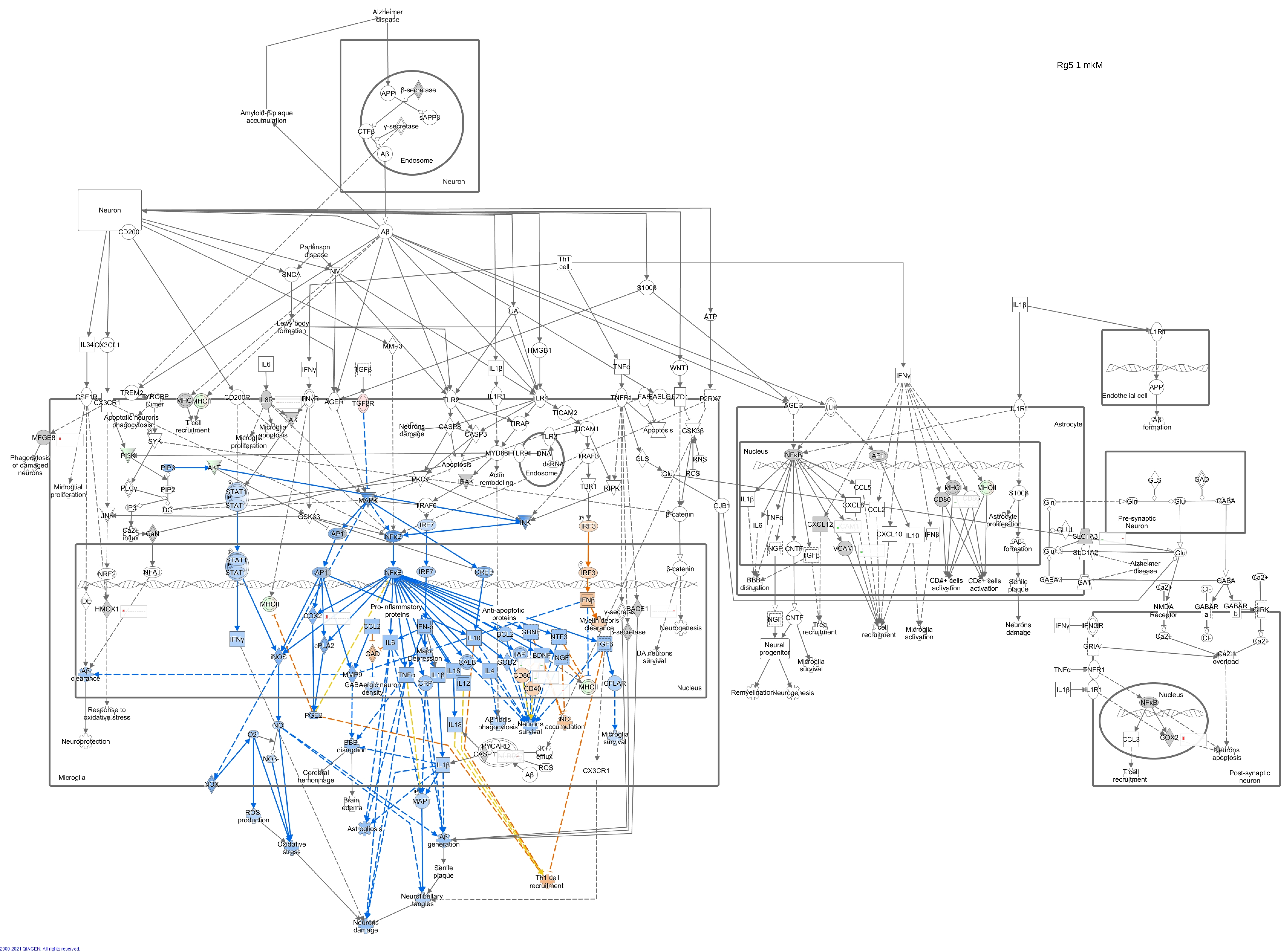

© 2000-2021 QIAGEN. All rights reserved.

Neuroinflammation involves numerous cell types, acts to clear neuronal damage, and plays a key role in maintaining the homeostasis of CNS.
Homeostasis can be lost through various regulatory failures, or when humoral immune components cross the blood-brain barrier, causing chronic inflammation with excessive cell and tissue damage, which is associated with neurodegenerative diseases.

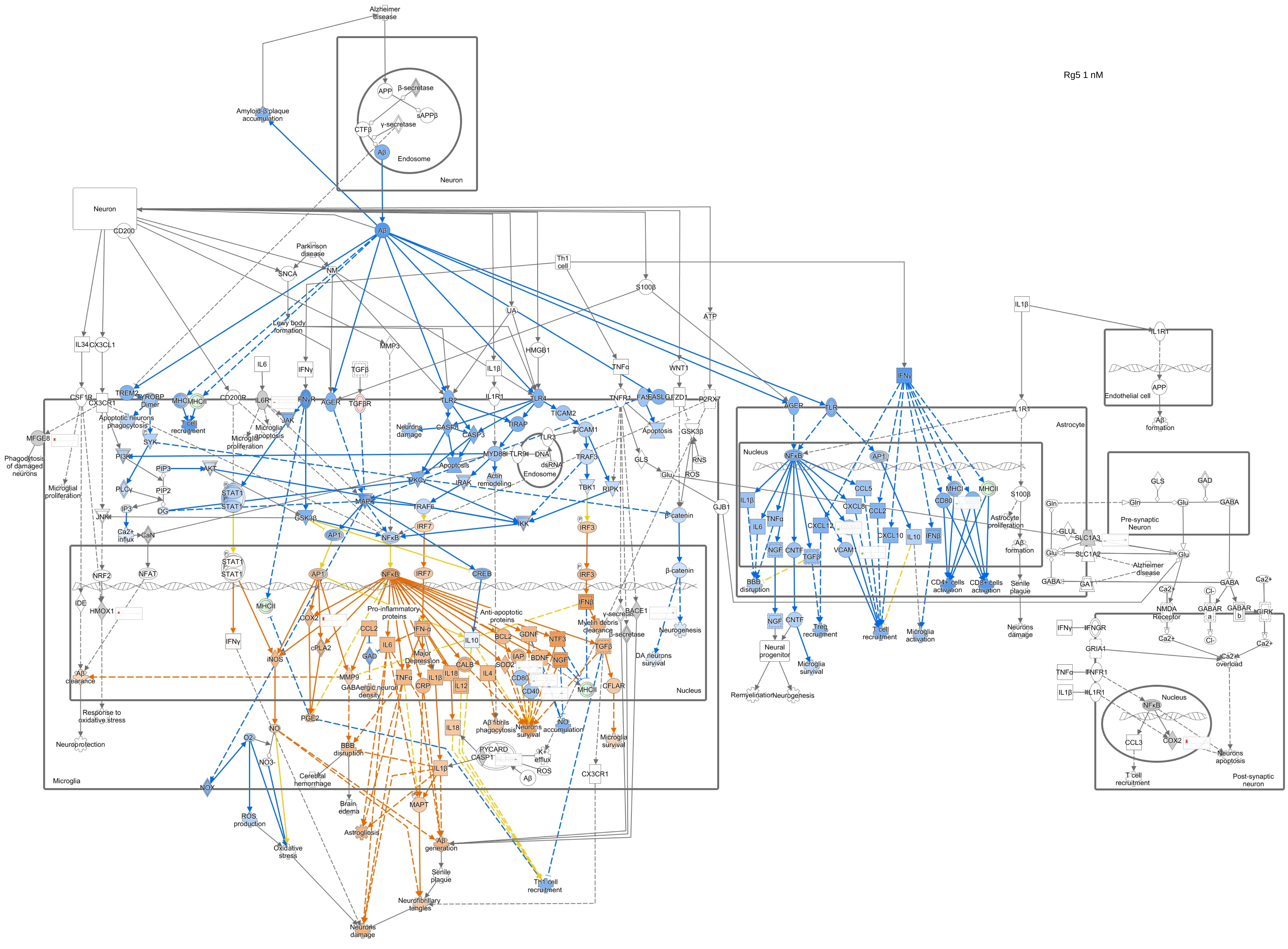

© 2000-2021 QIAGEN. All rights reserved.

Neuroinflammation Signaling Pathway

Neuroinflammation involves numerous cell types, acts to clear neuronal damage, and plays a key role in maintaining the homeostasis of CNS. Homeostasis can be lost through various regulatory failures, or when humoral immune components cross the blood-brain barrier, causing chronic inflammation with excessive cell and tissue damage, which is associated with neurodegenerative diseases.

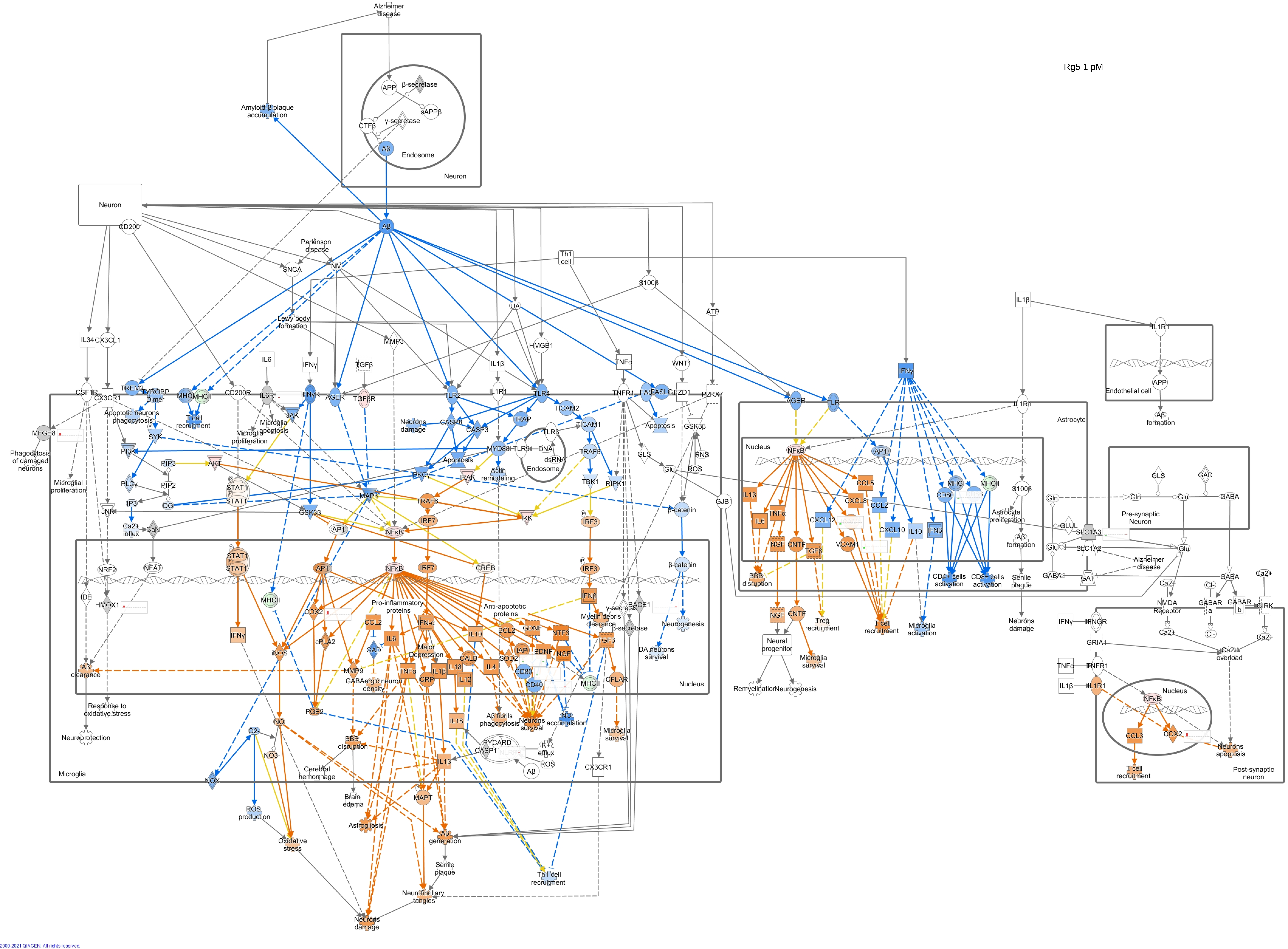

© 2000-2021 QIAGEN. All rights reserved.

Neuroinflammation Signaling Pathway

Neuroinflammation involves numerous cell types, acts to clear neuronal damage, and plays a key role in maintaining the homeostasis of CNS. Homeostasis can be lost through various regulatory failures, or when humoral immune components cross the blood-brain barrier, causing chronic inflammation with excessive cell and tissue damage, which is associated with neurodegenerative diseases.

Rg5 1 fM

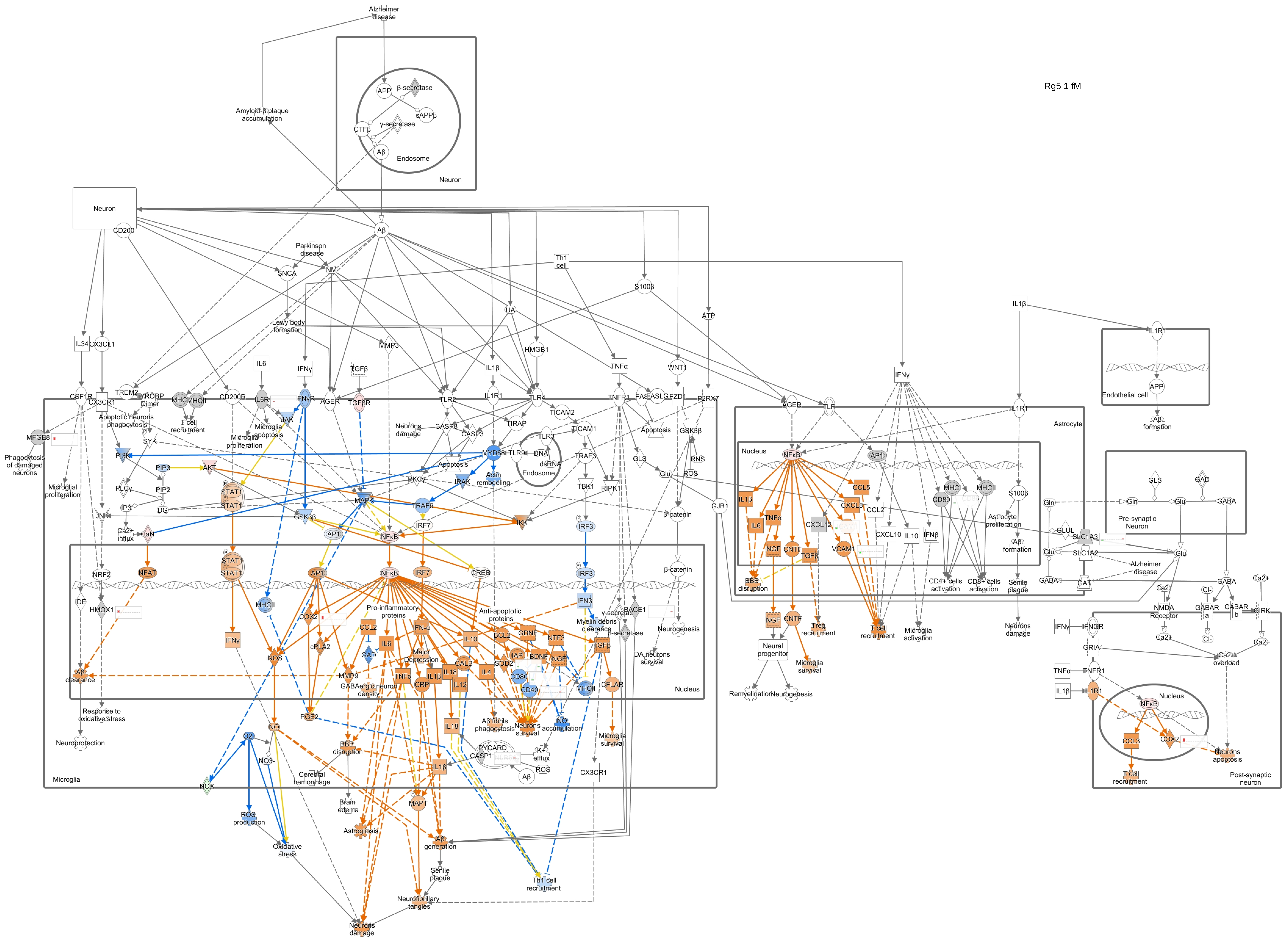

© 2000-2021 QIAGEN. All rights reserved.

Neuroinflammation involves numerous cell types, acts to clear neuronal damage, and plays a key role in maintaining the homeostasis of CNS. Homeostasis can be lost through various regulatory failures, or when humoral immune components cross the blood-brain barrier, causing chronic inflammation with excessive cell and tissue damage, which is associated with neurodegenerative diseases.

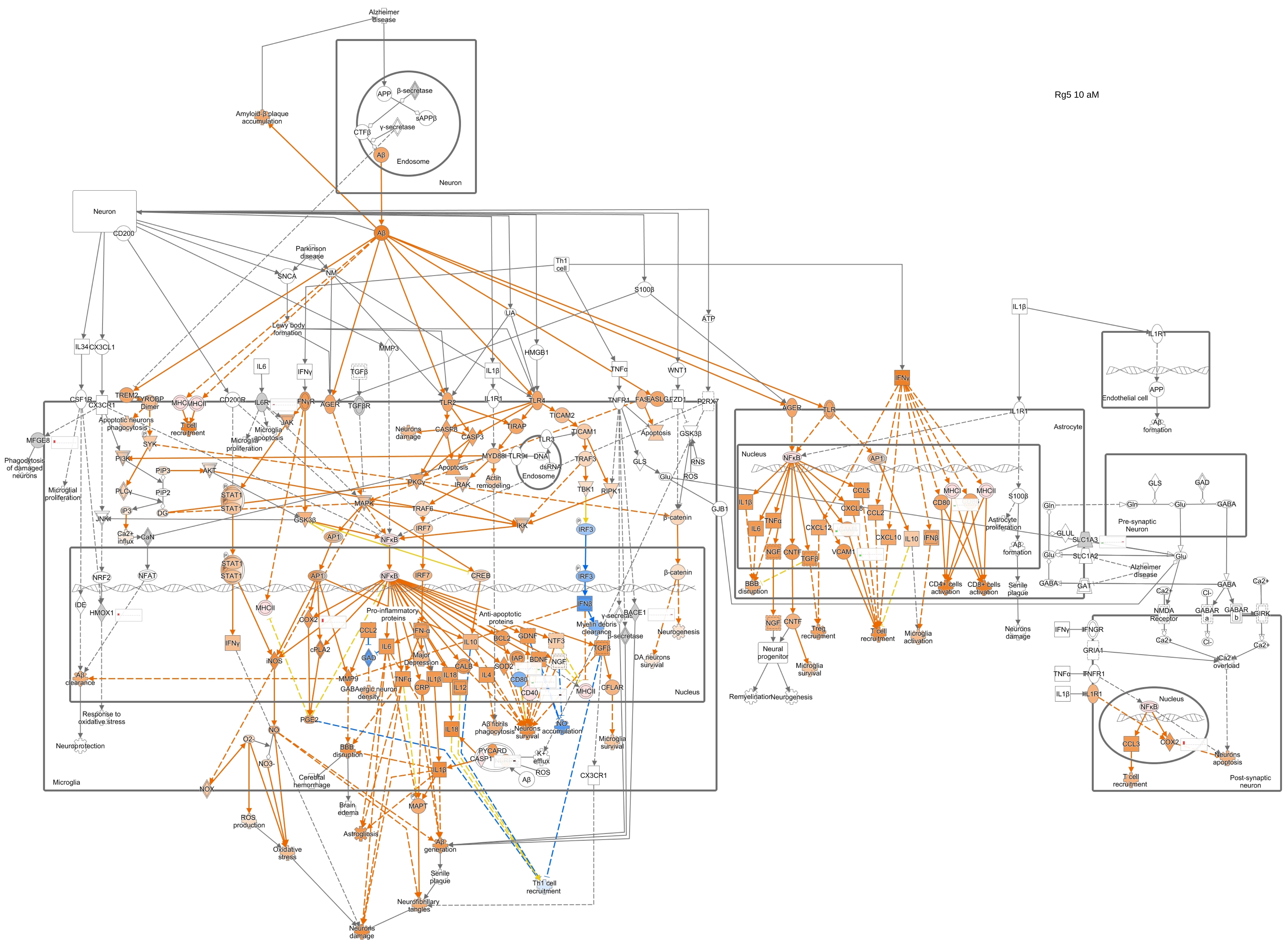

© 2000-2021 QIAGEN. All rights reserved.

Neuroinflammation Signaling Pathway

Neuroinflammation involves numerous cell types, acts to clear neuronal damage, and plays a key role in maintaining the homeostasis of CNS. Homeostasis can be lost through various regulatory failures, or when humoral immune components cross the blood-brain barrier, causing chronic inflammation with excessive cell and tissue damage, which is associated with neurodegenerative diseases.

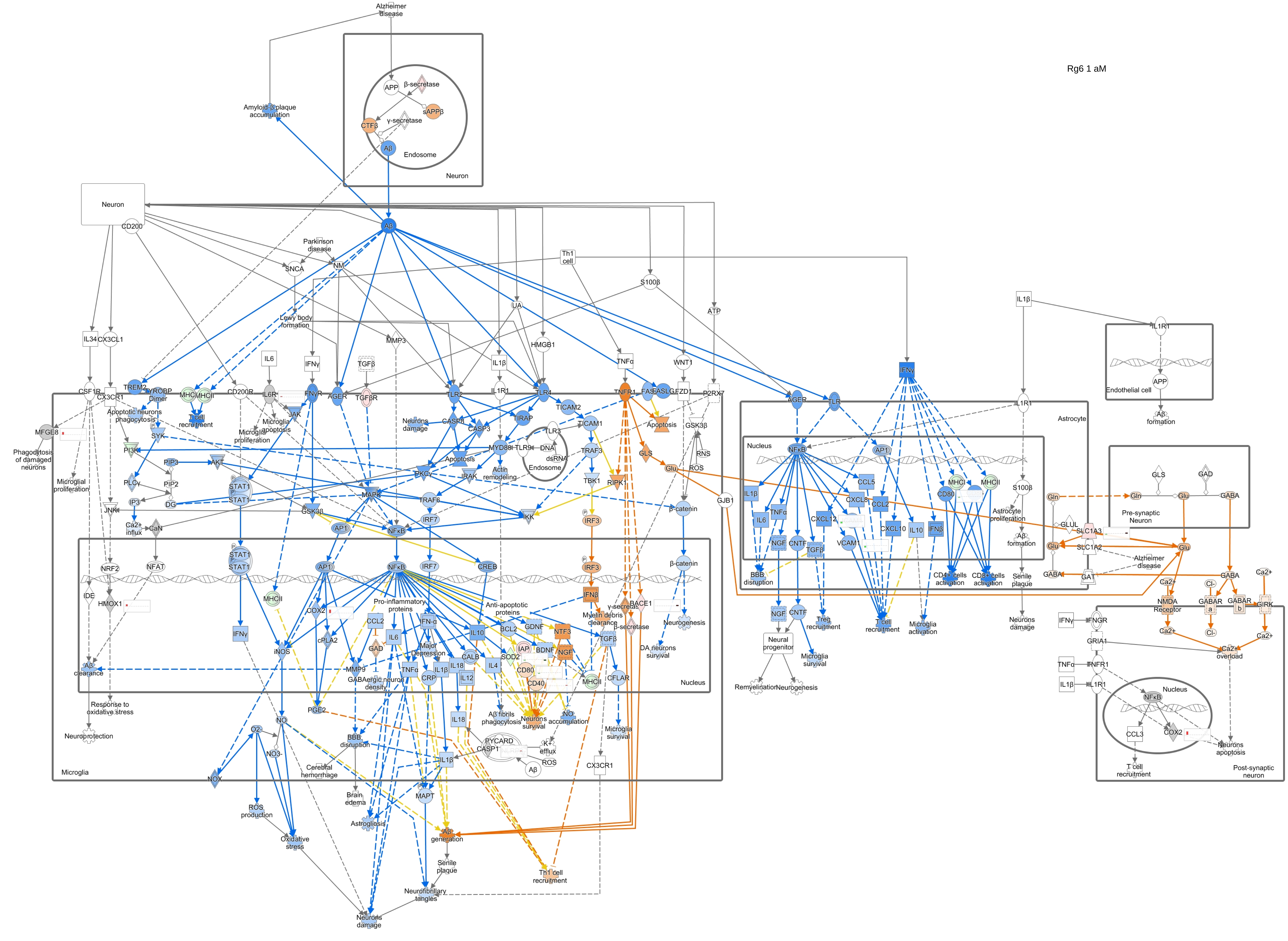

© 2000-2021 QIAGEN. All rights reserved.
