# Supplementary figures and images for: Network Pharmacology of Red Ginseng (Part I): Effects of Ginsenoside Rg5 at Physiological and Sub-Physiological Concentrations
Source: Pharmaceuticals (Basel). 2021 Sep 29;14(10):999. doi: 10.3390/ph14100999 (PMC8537973; doi:10.3390/ph14100999)

100  $\mu$ M

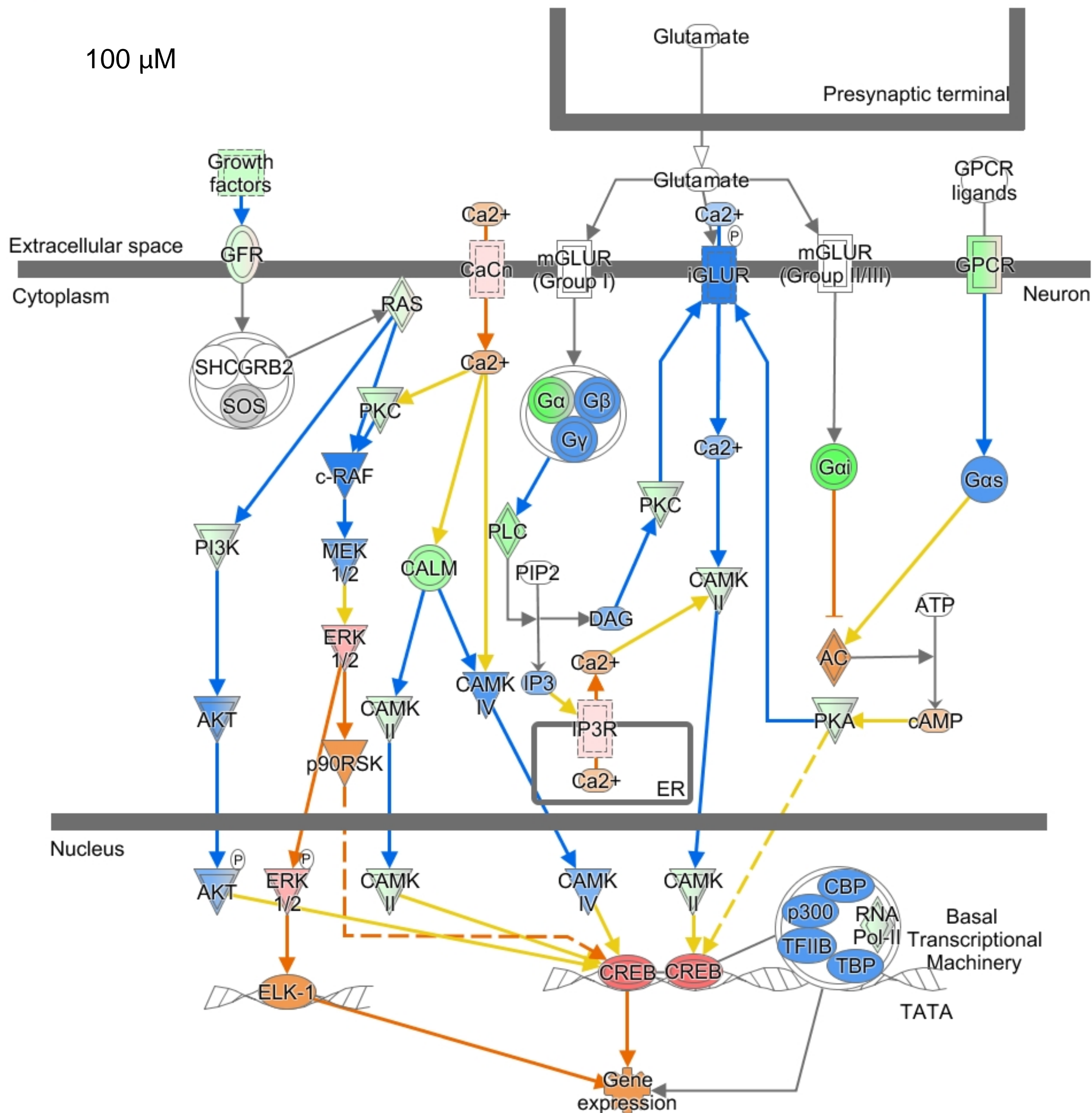

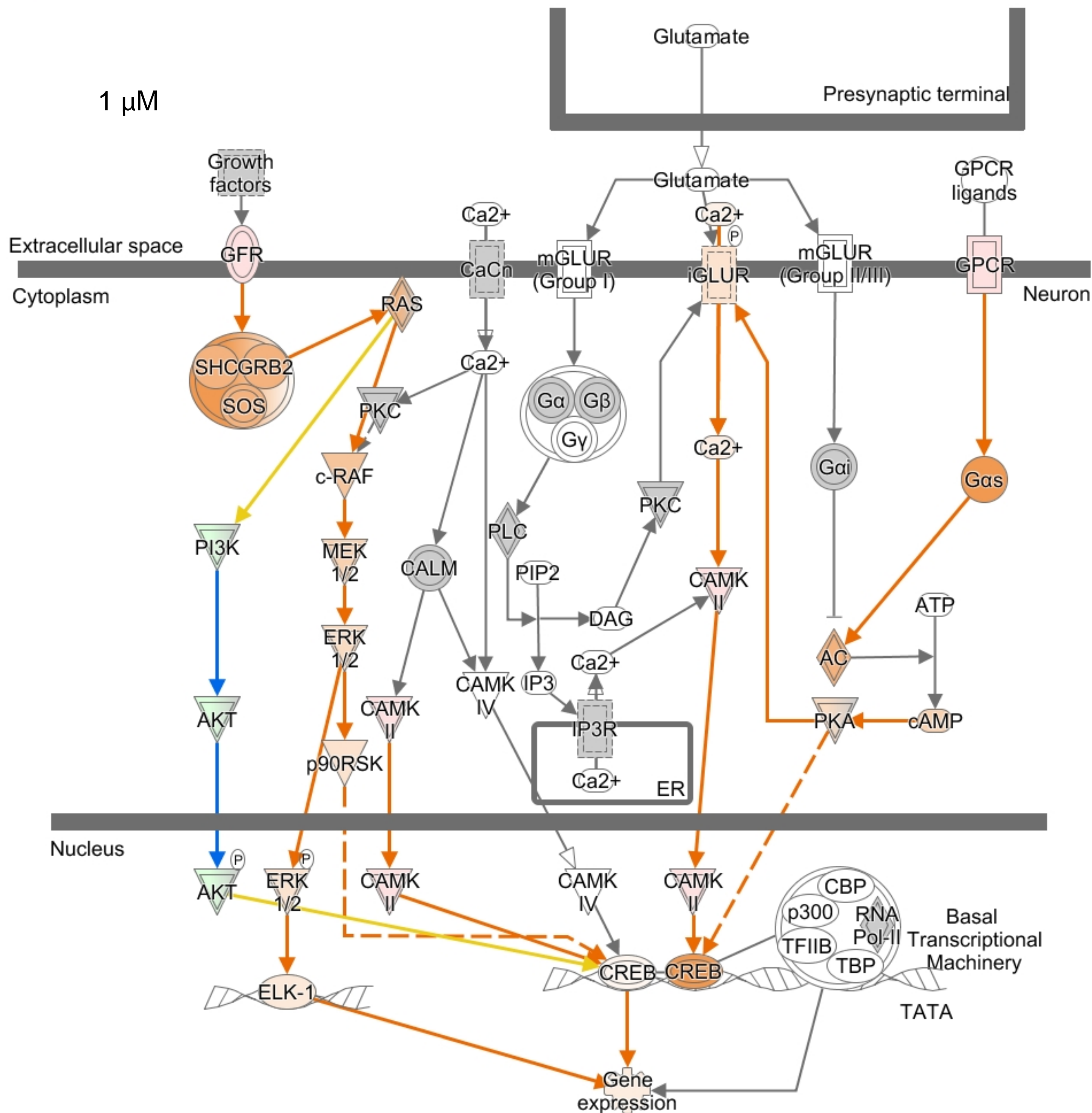

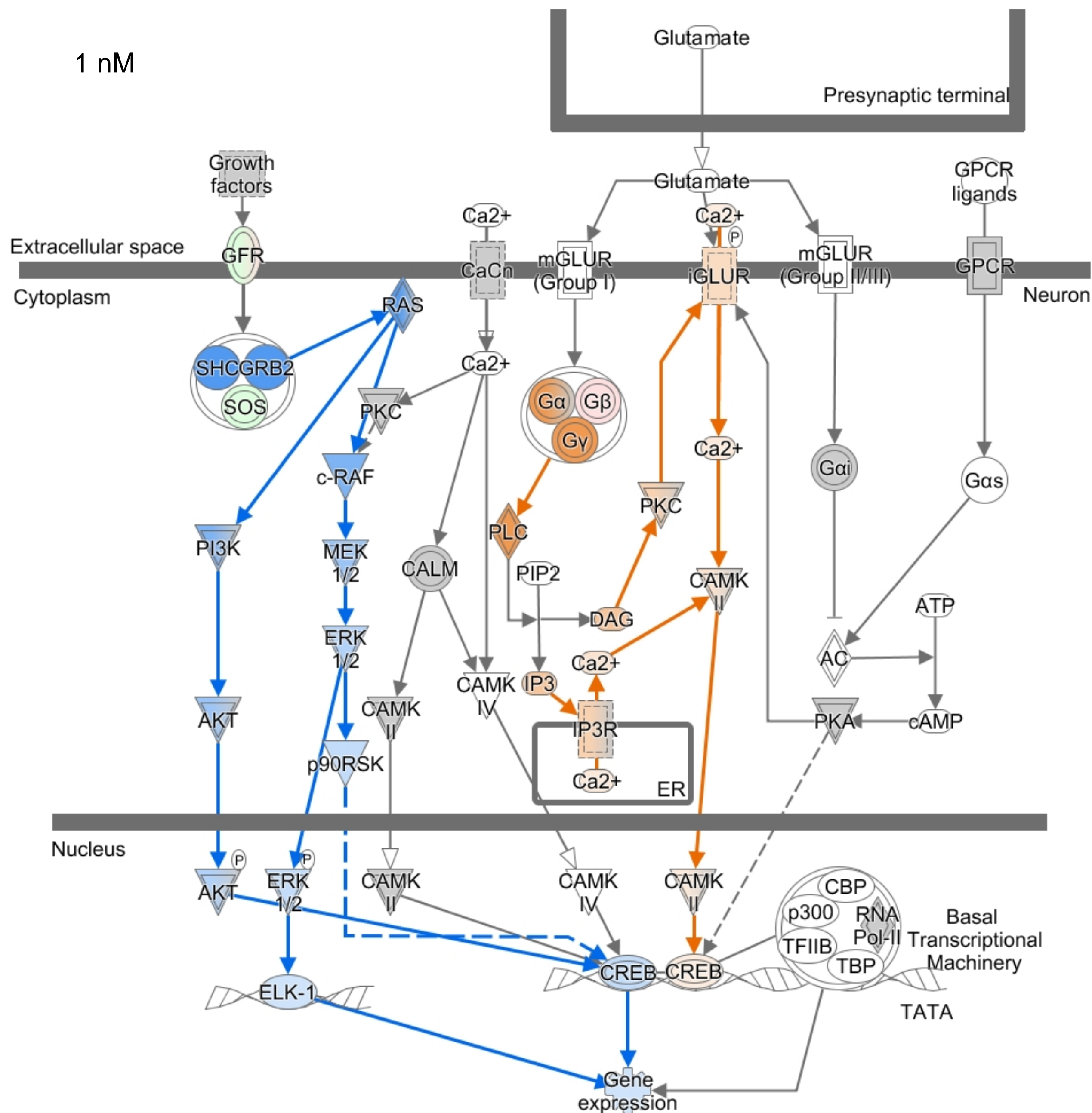

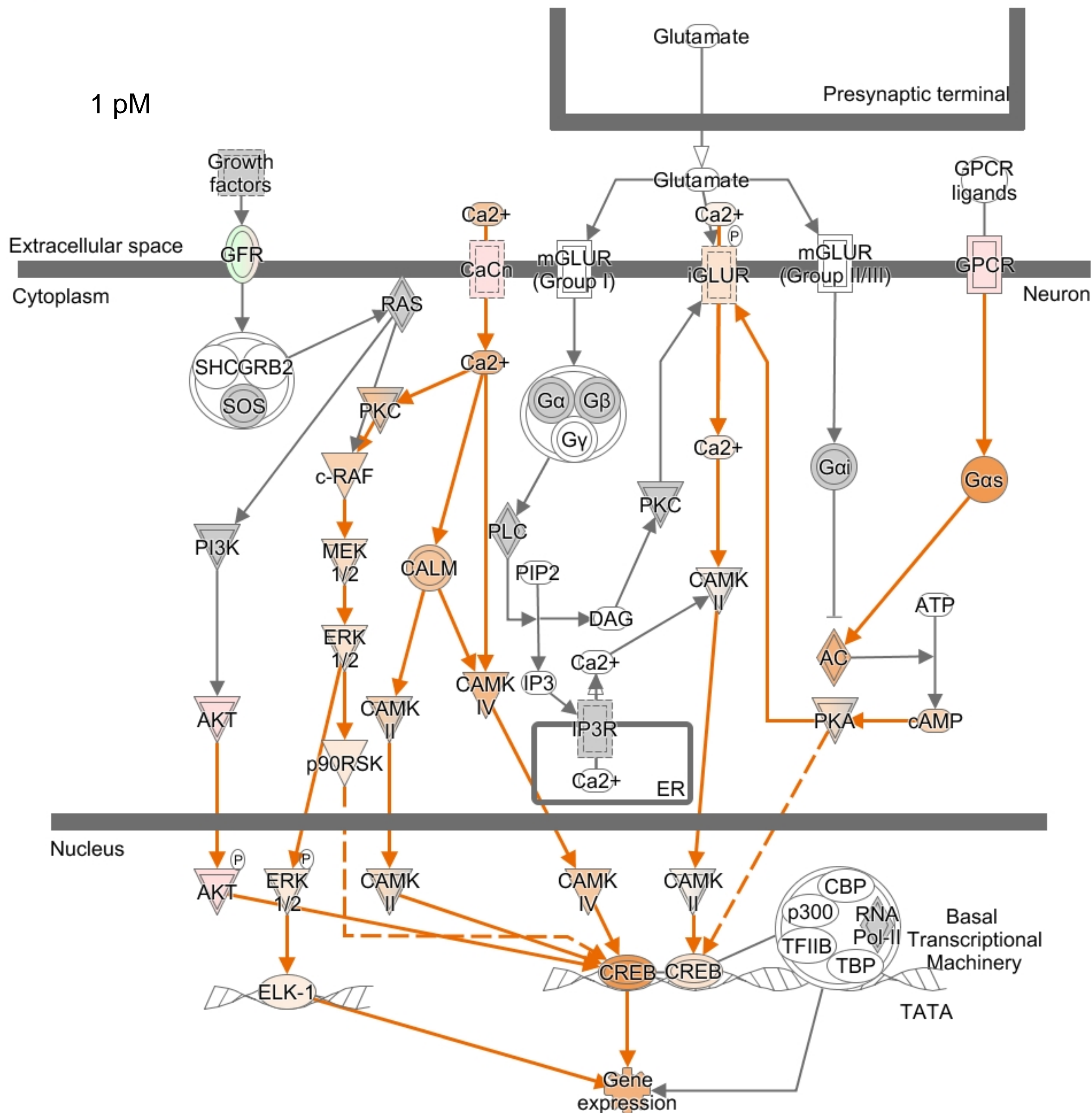

1 fM

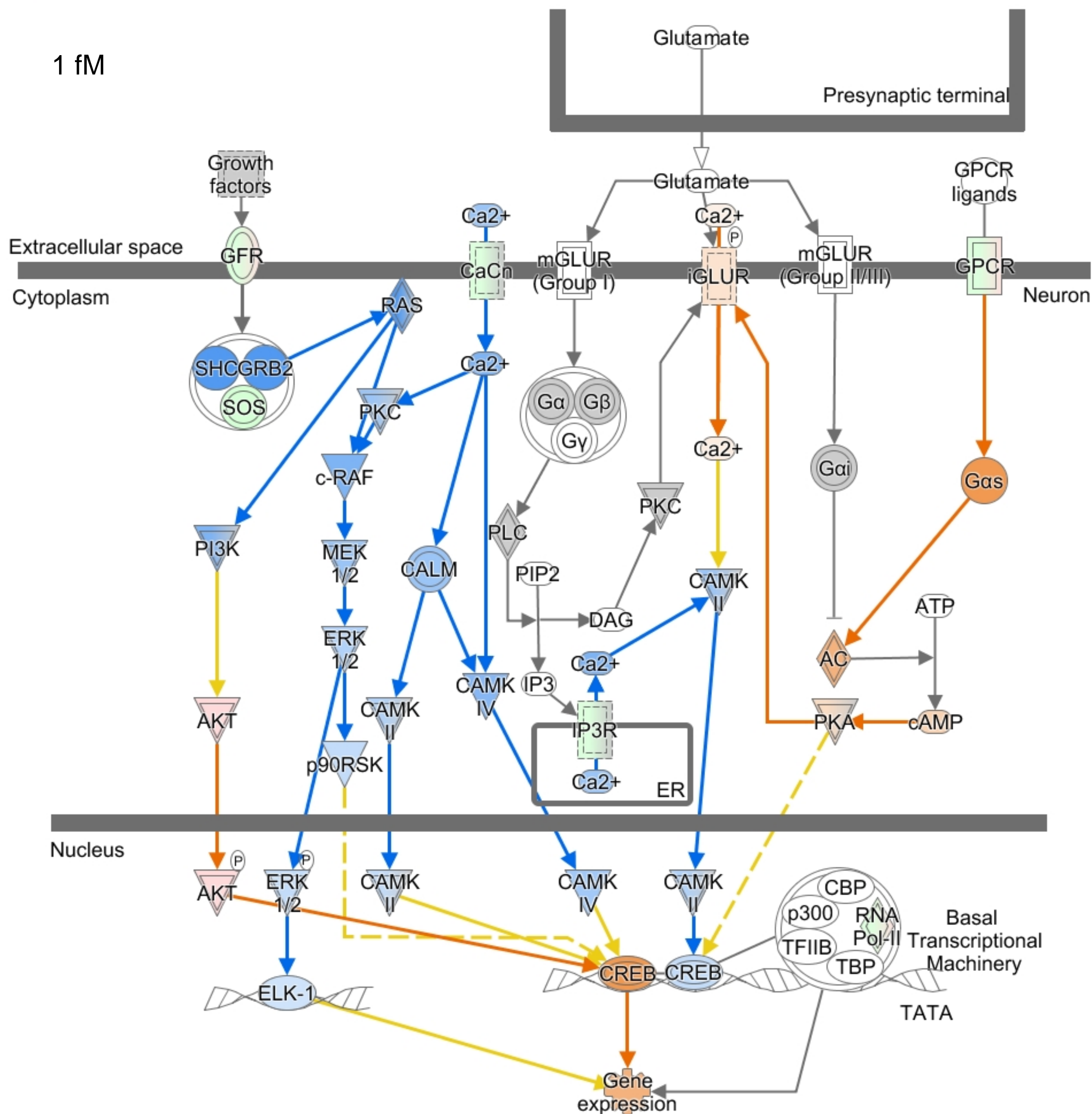

10 aM

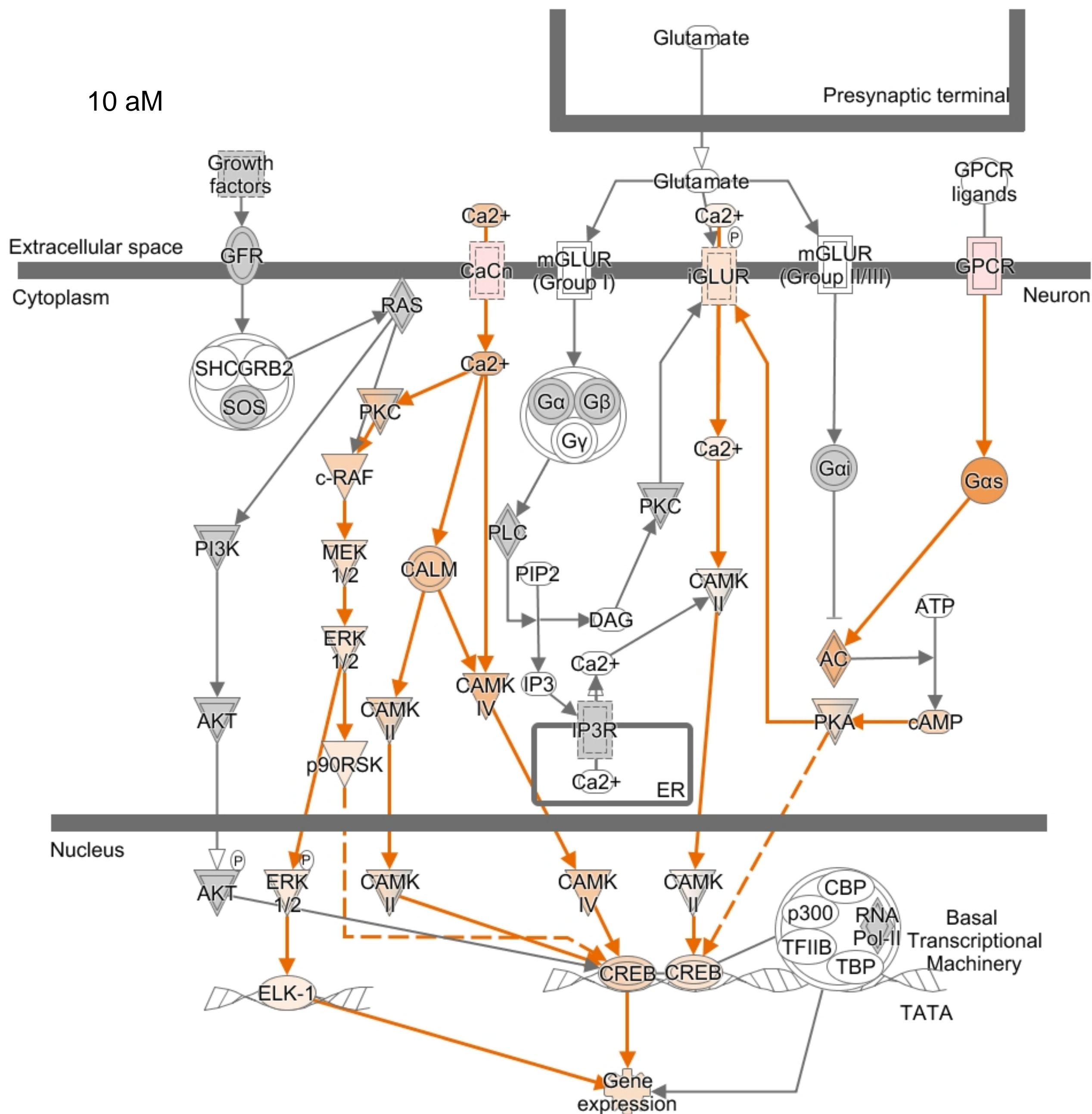

1 aM

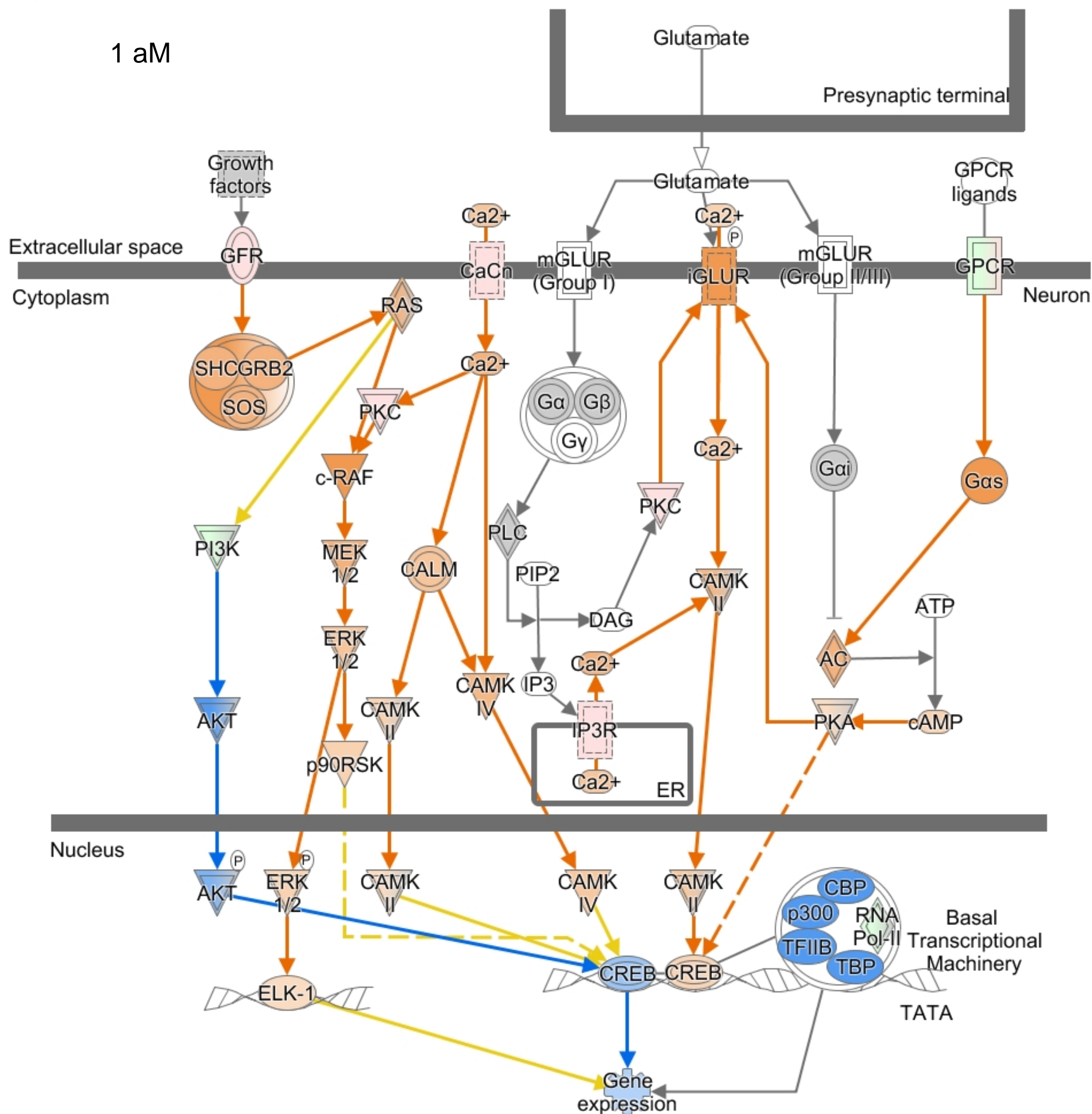

Supplement: Supplementary file 1 [file pharmaceuticals-14-00999-s001.zip › Suppl 4 CREB signalling.pdf]

100  $\mu$ M

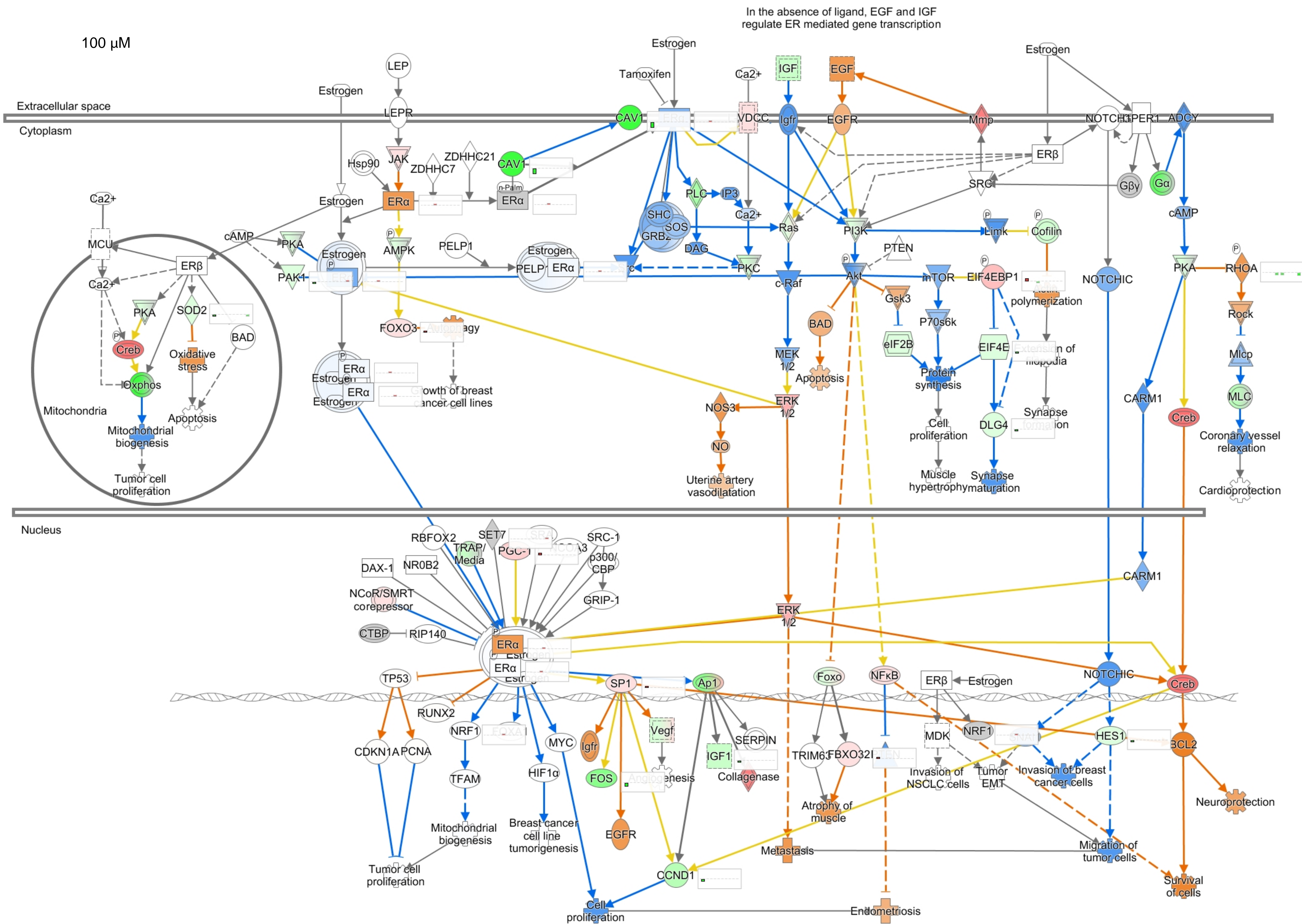

1  $\mu$ M

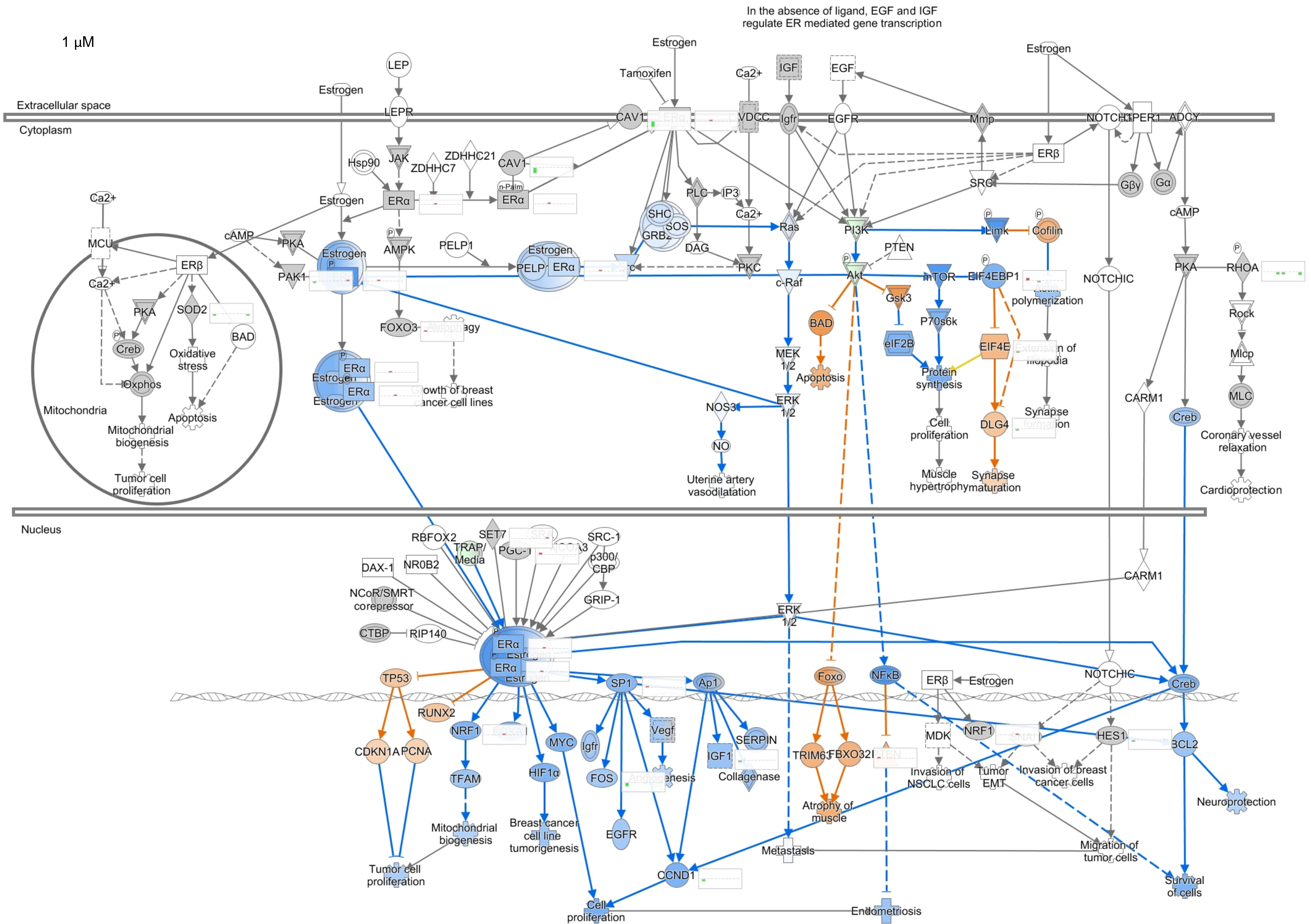

1 nM

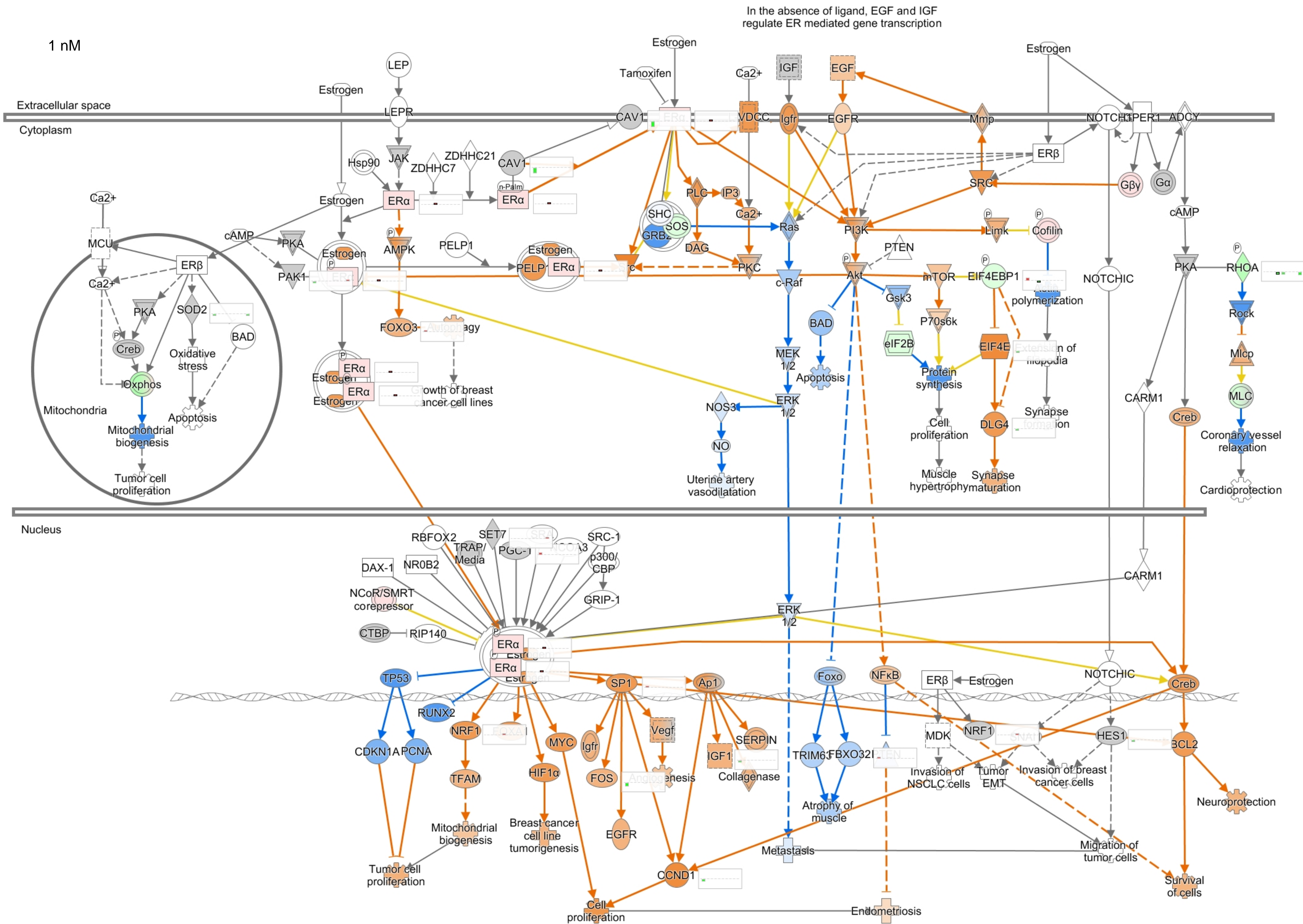

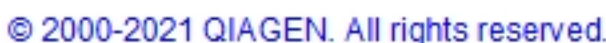

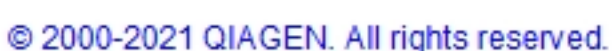

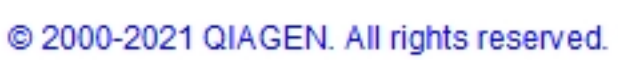

1 aM

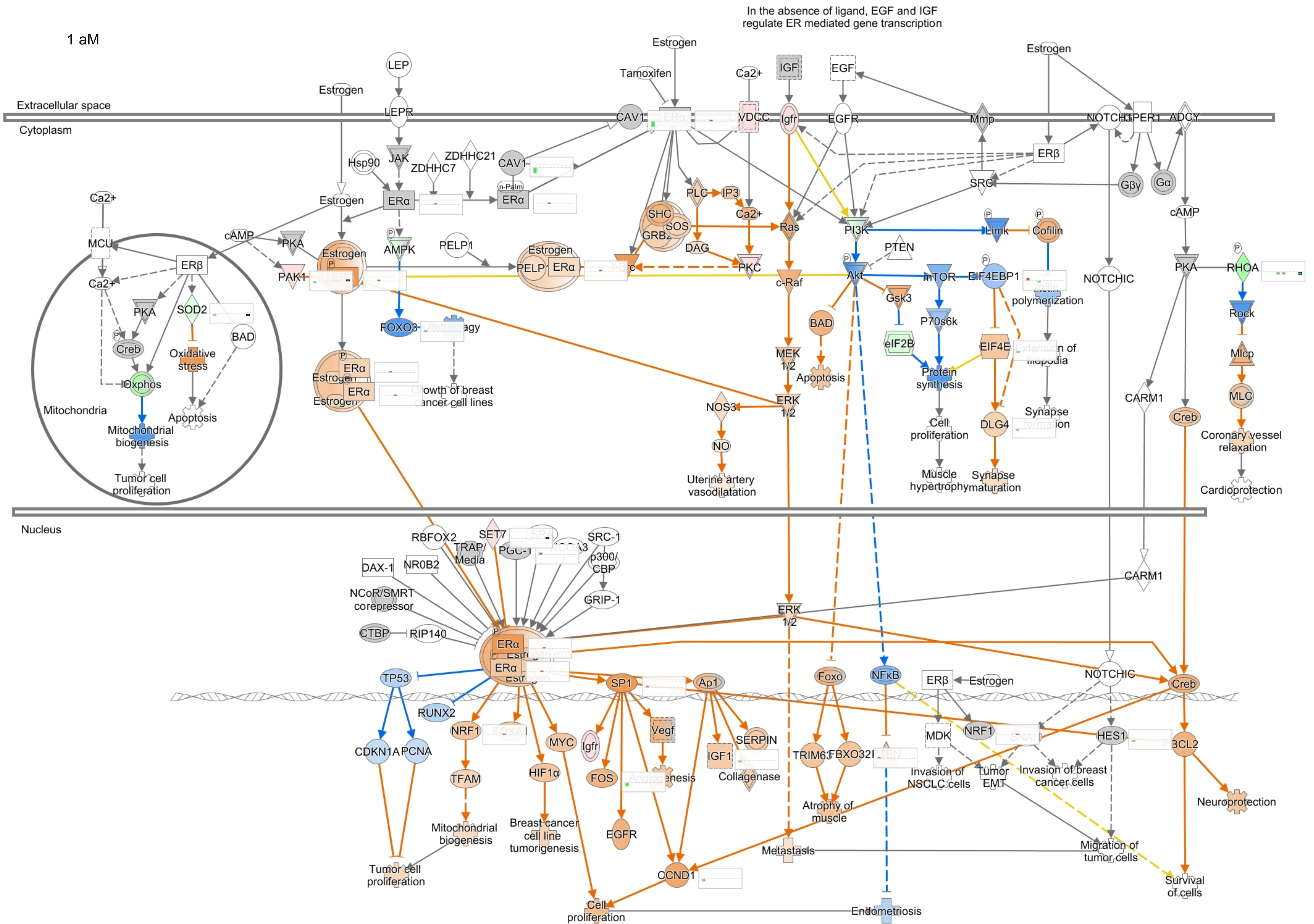

Supplement: Supplementary file 1 [file pharmaceuticals-14-00999-s001.zip › Suppl 6 ER signaling.pdf]

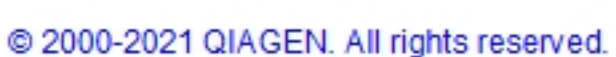

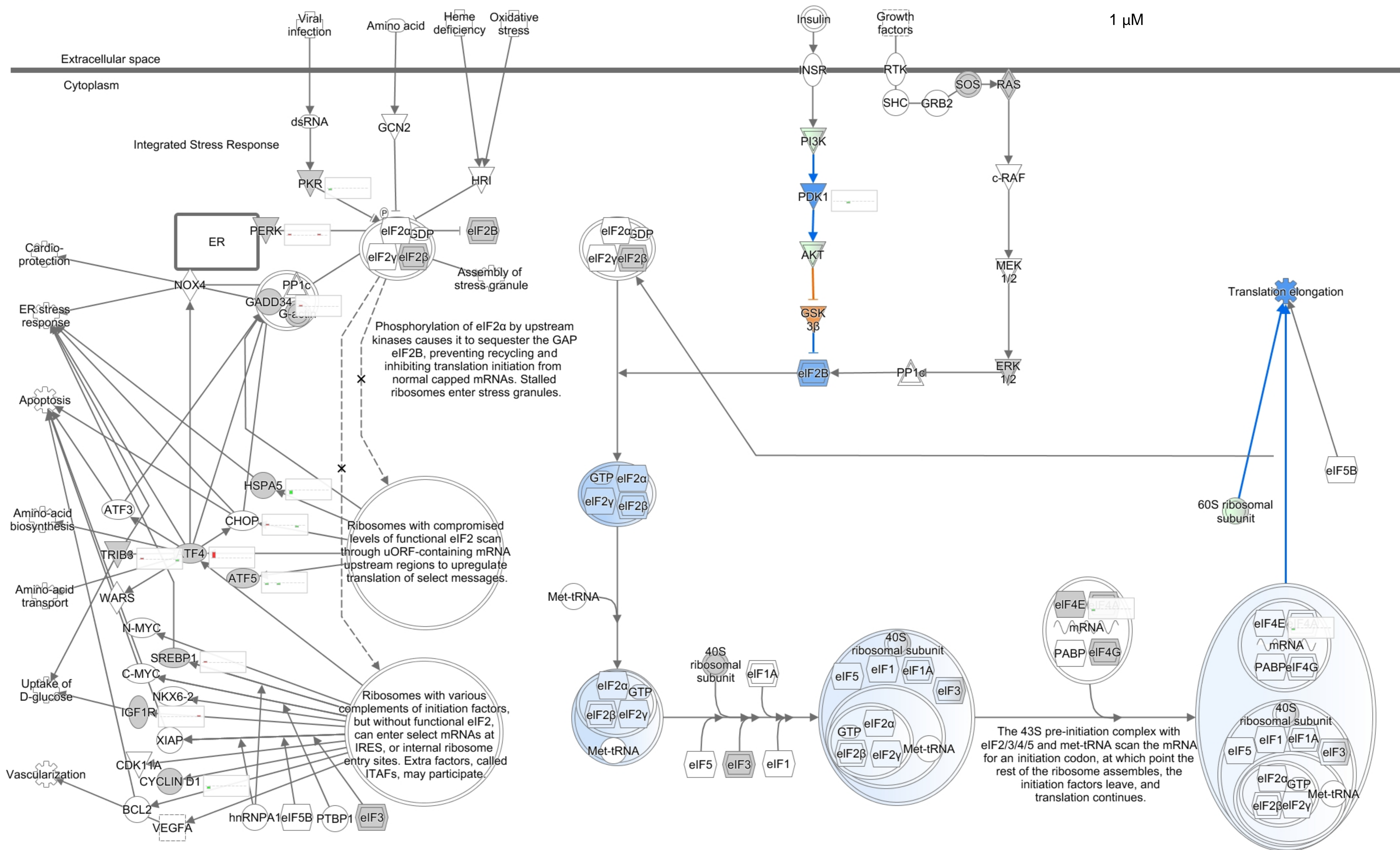

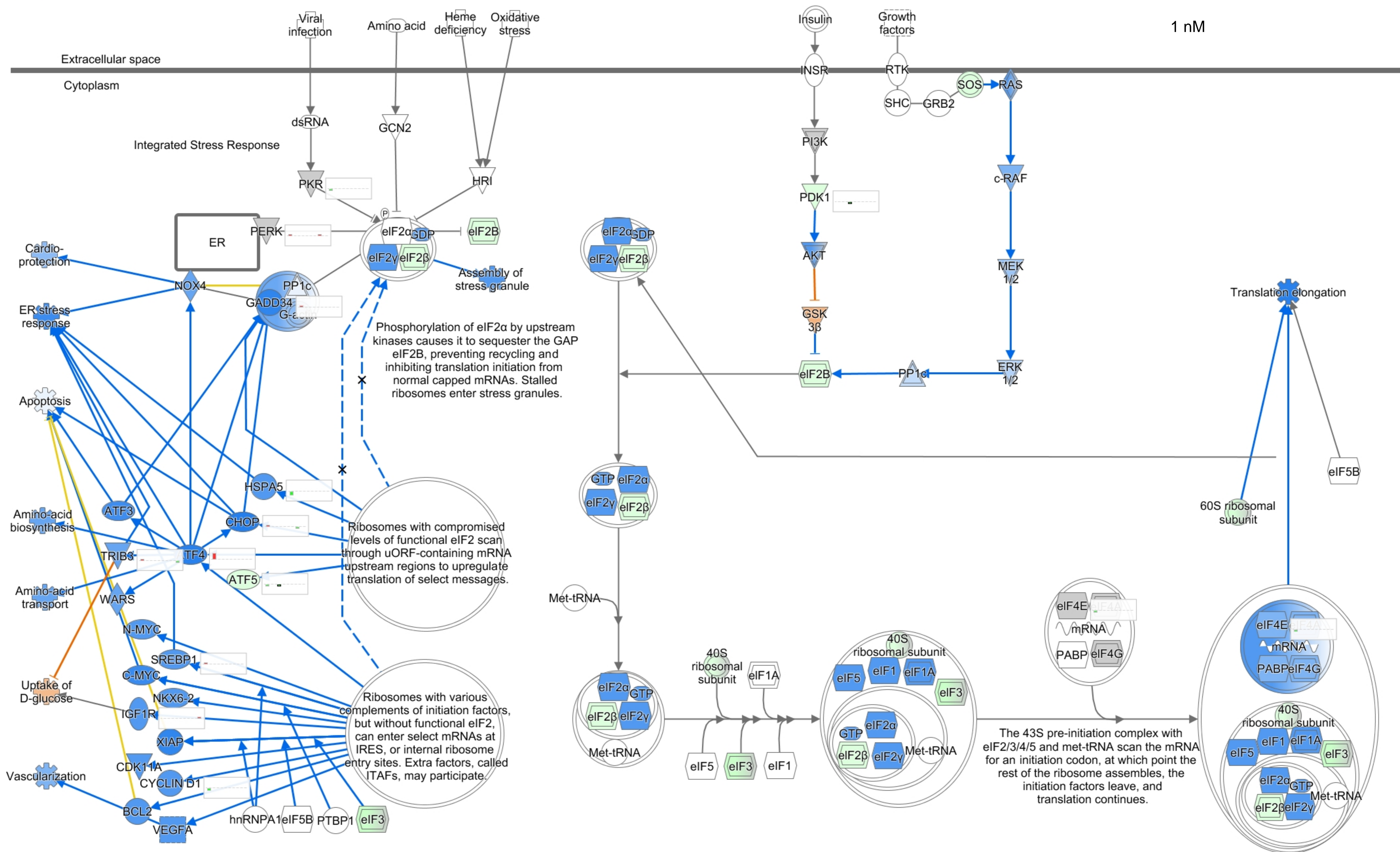

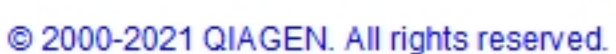

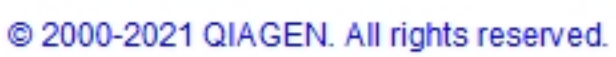

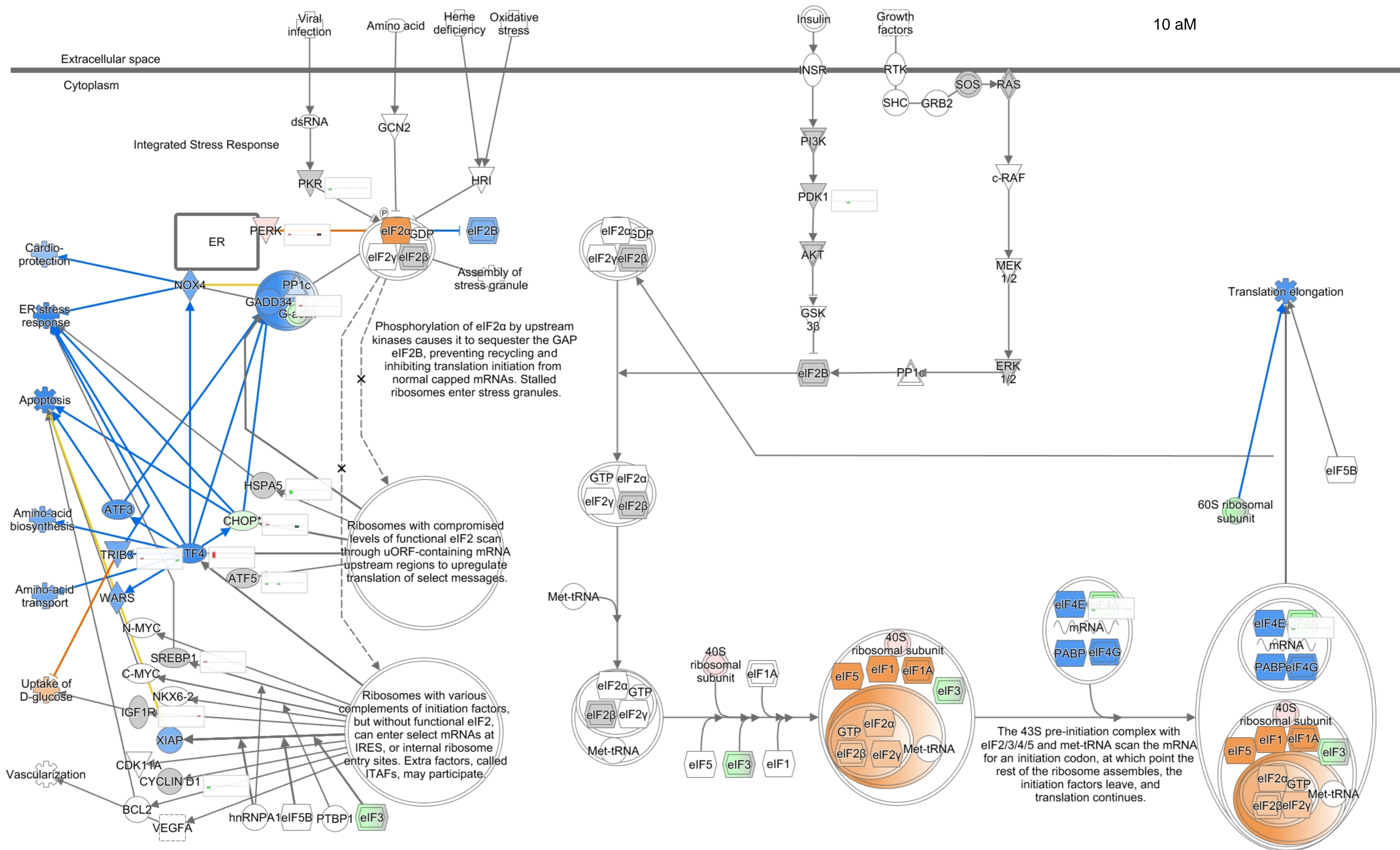

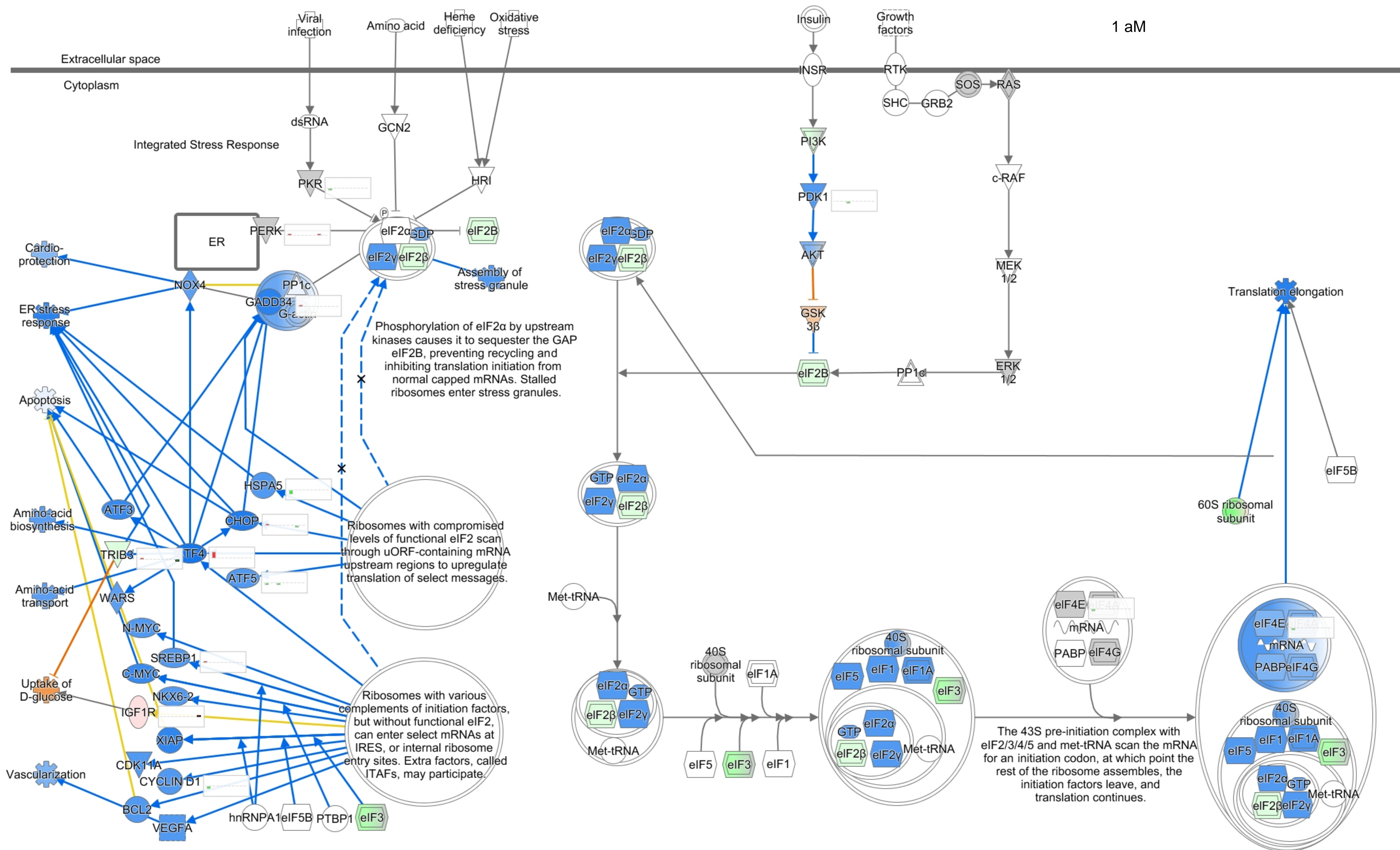

Supplement: Supplementary file 1 [file pharmaceuticals-14-00999-s001.zip › Suppl 8 EIF2 signaling.pdf]

100  $\mu$ M

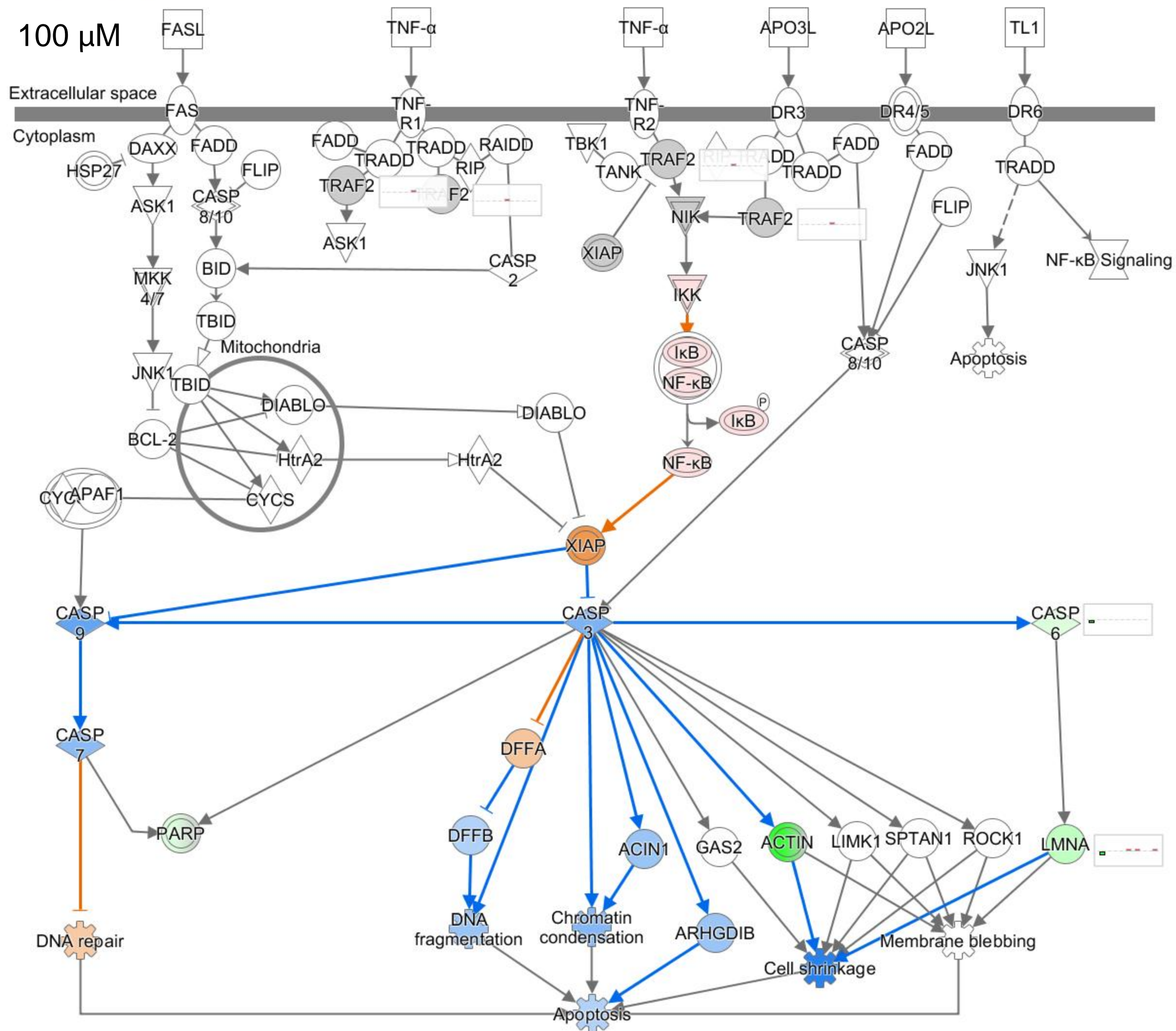

1  $\mu$ M

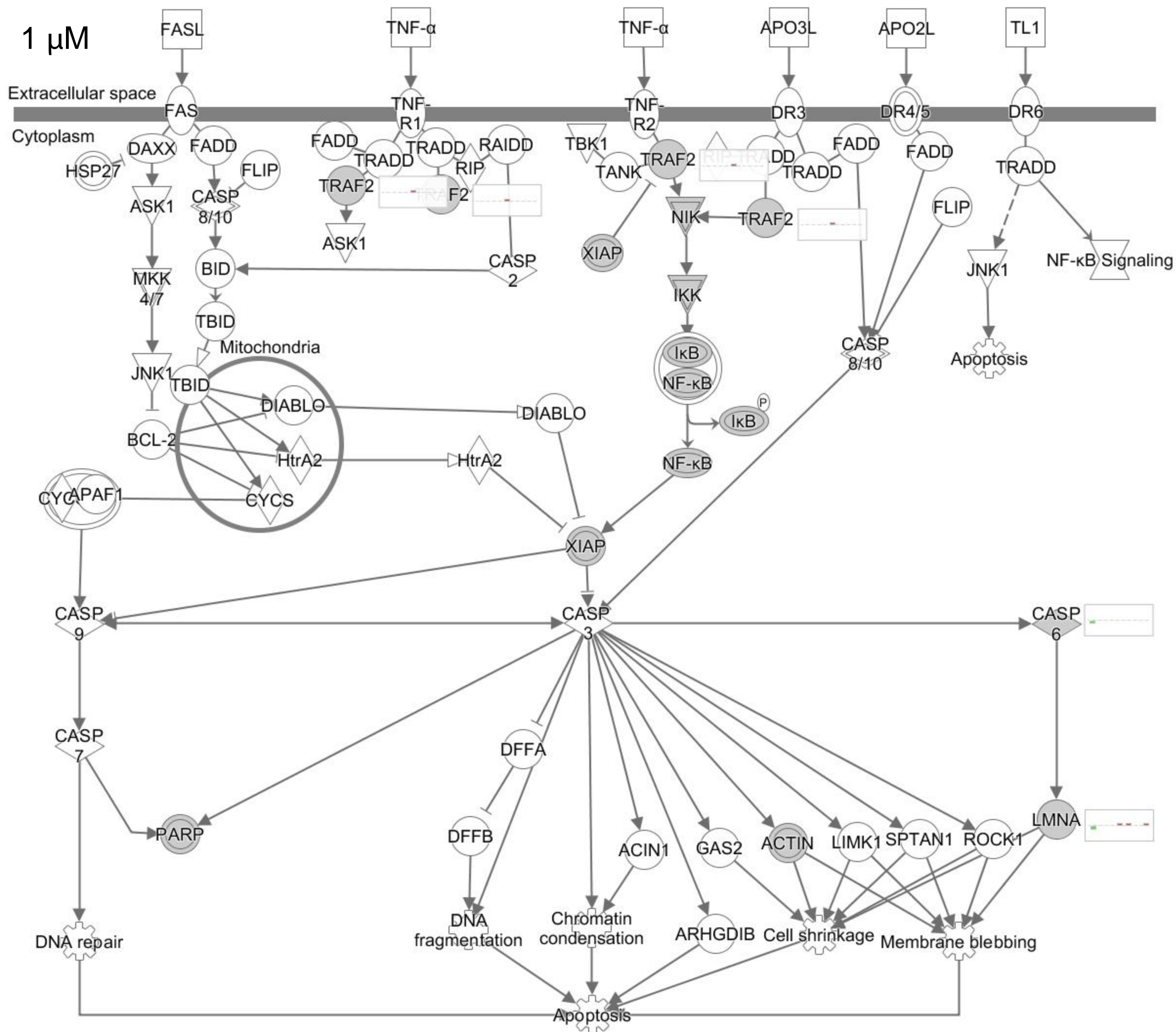

1 nM

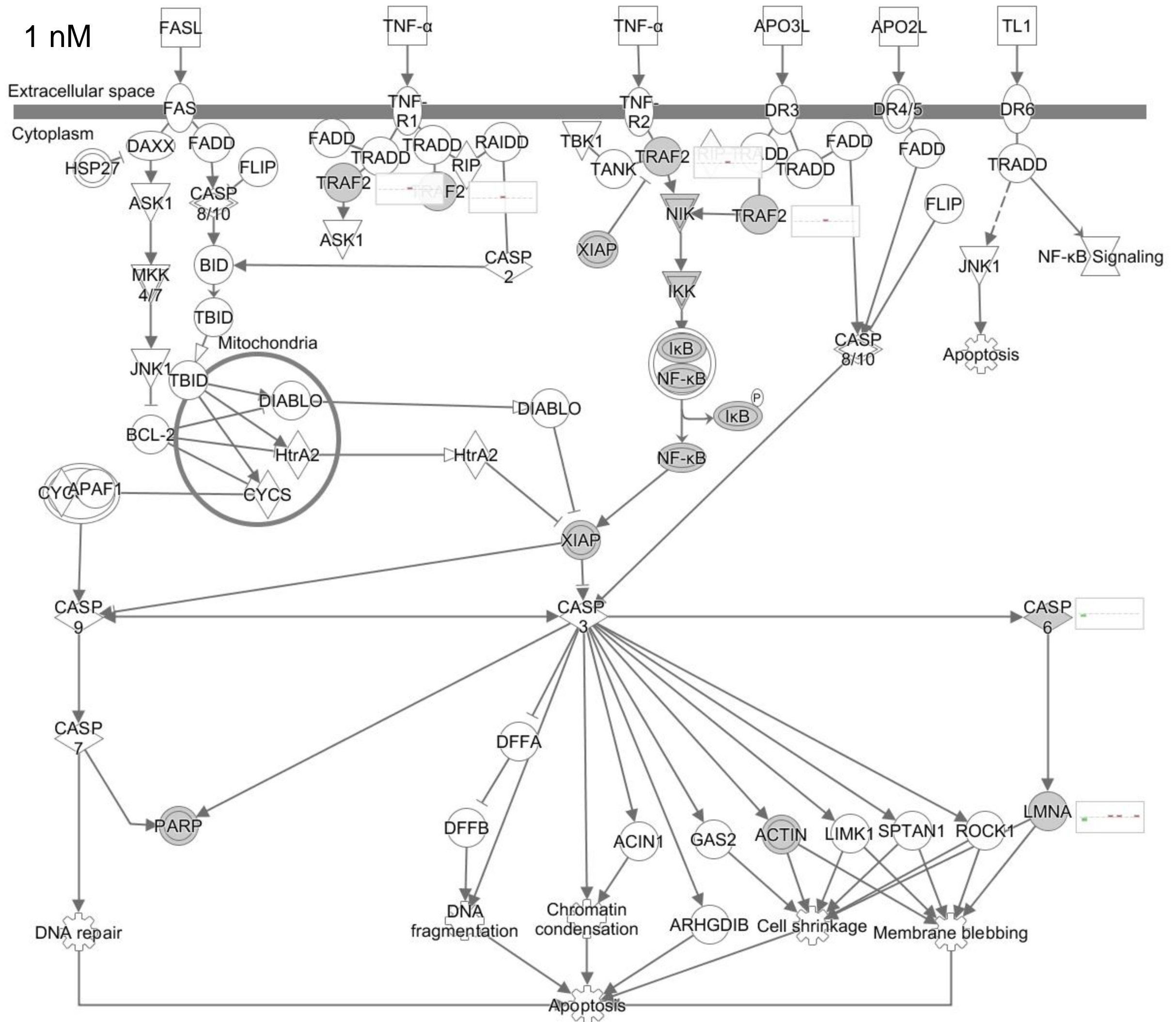

1 pM

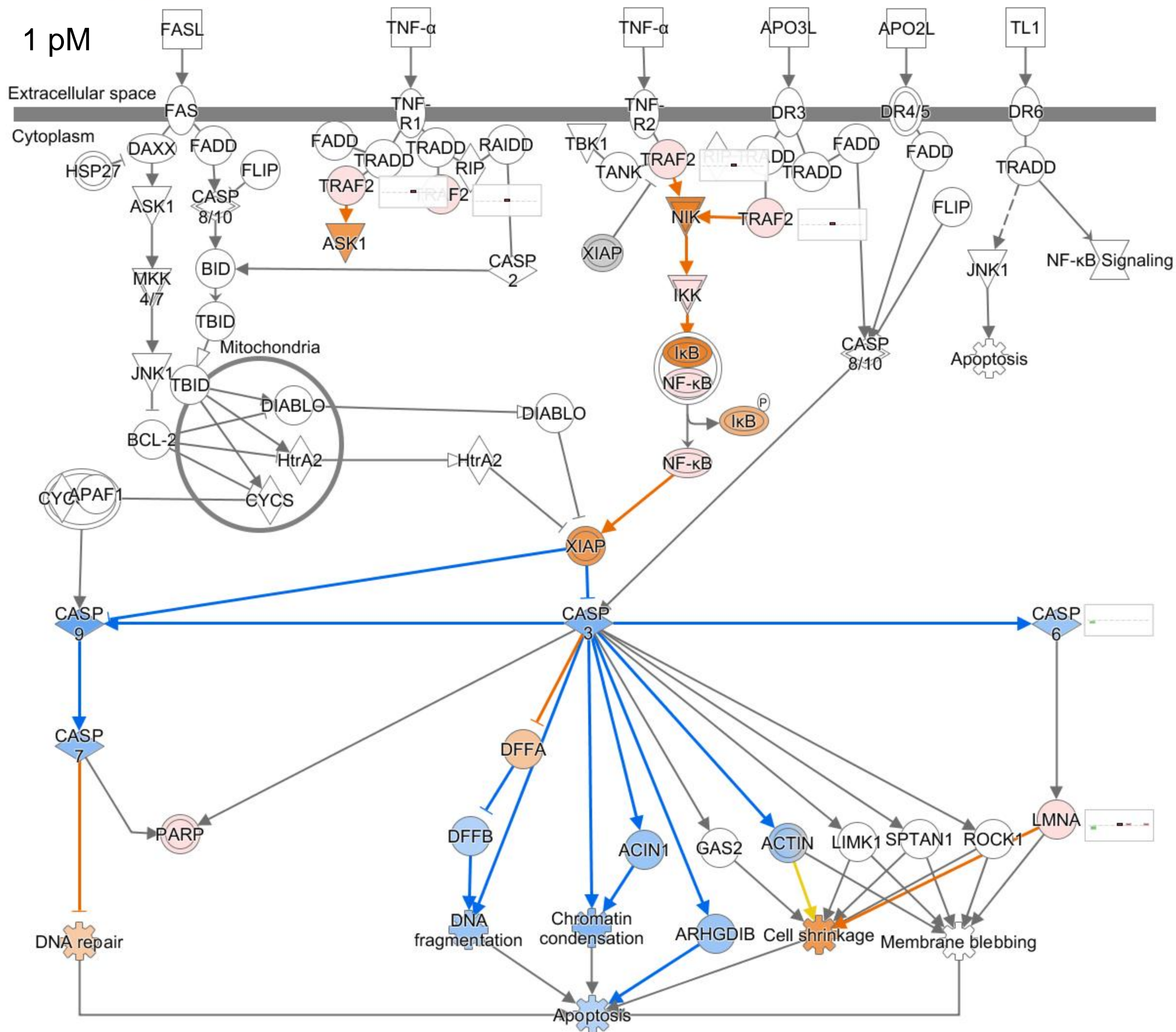

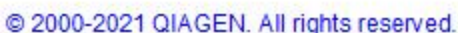

10 aM

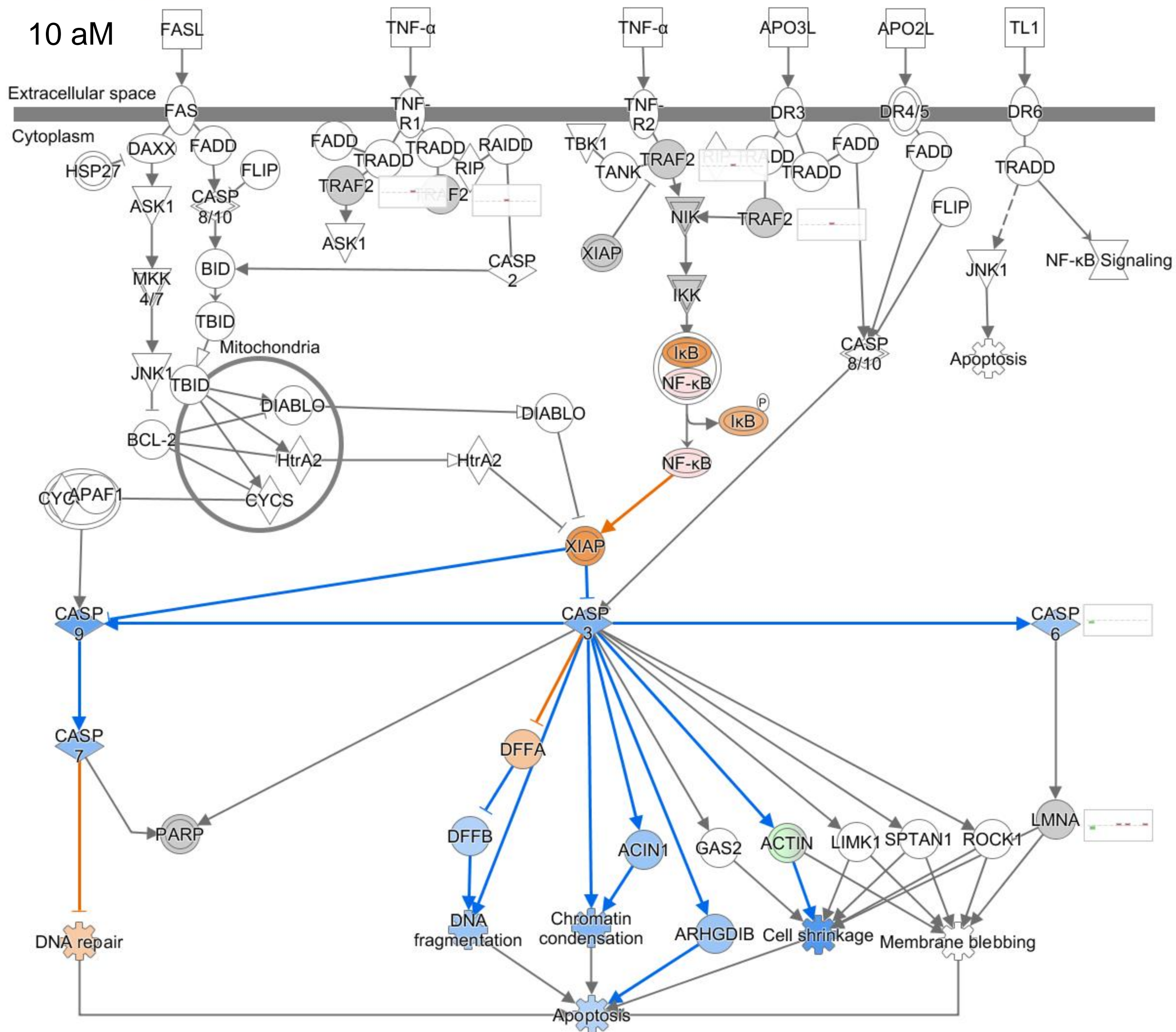

1 aM

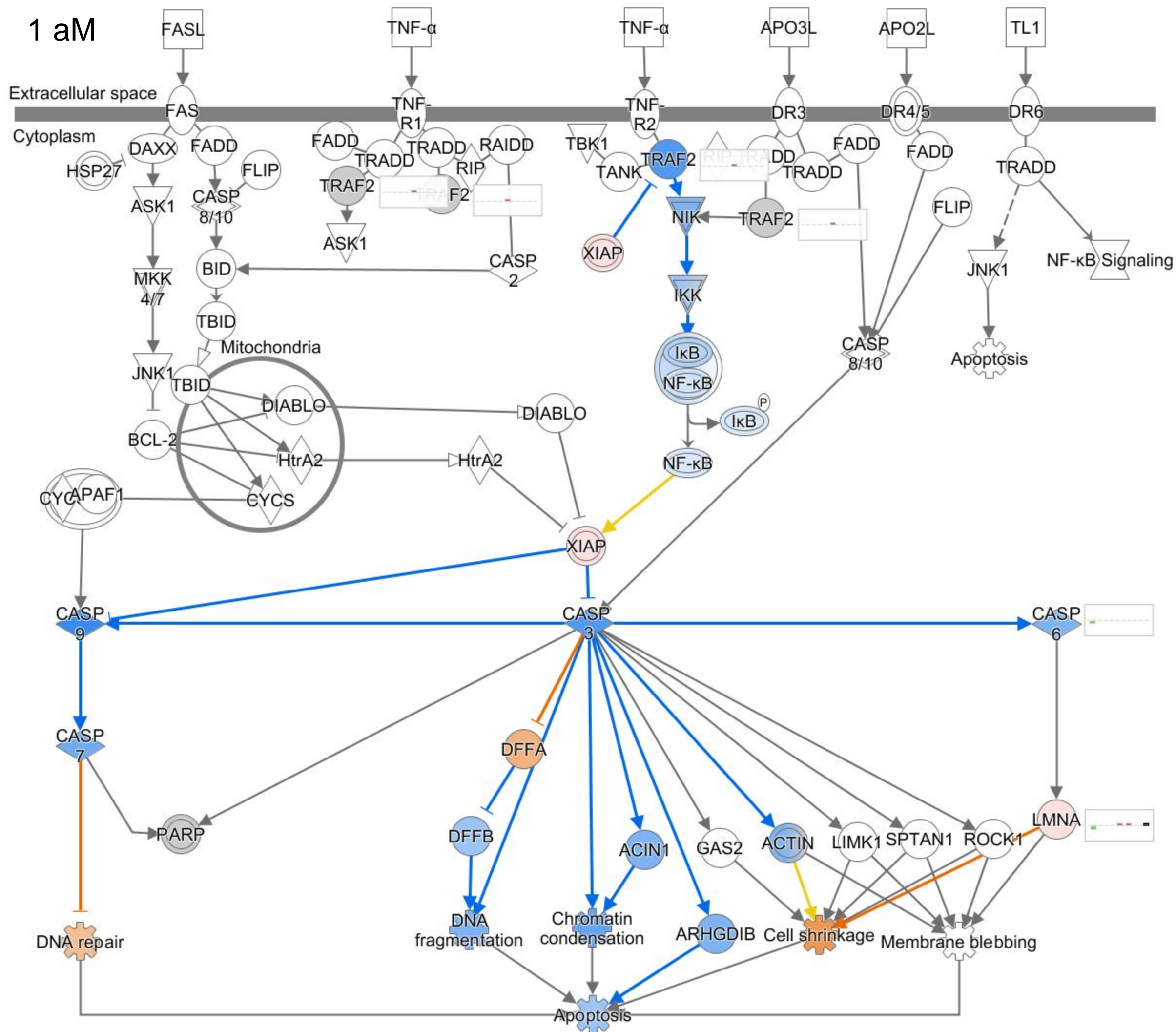

Supplement: Supplementary file 1 [file pharmaceuticals-14-00999-s001.zip › Suppl 9 Death signaling pathway.pdf]

100  $\mu$ M

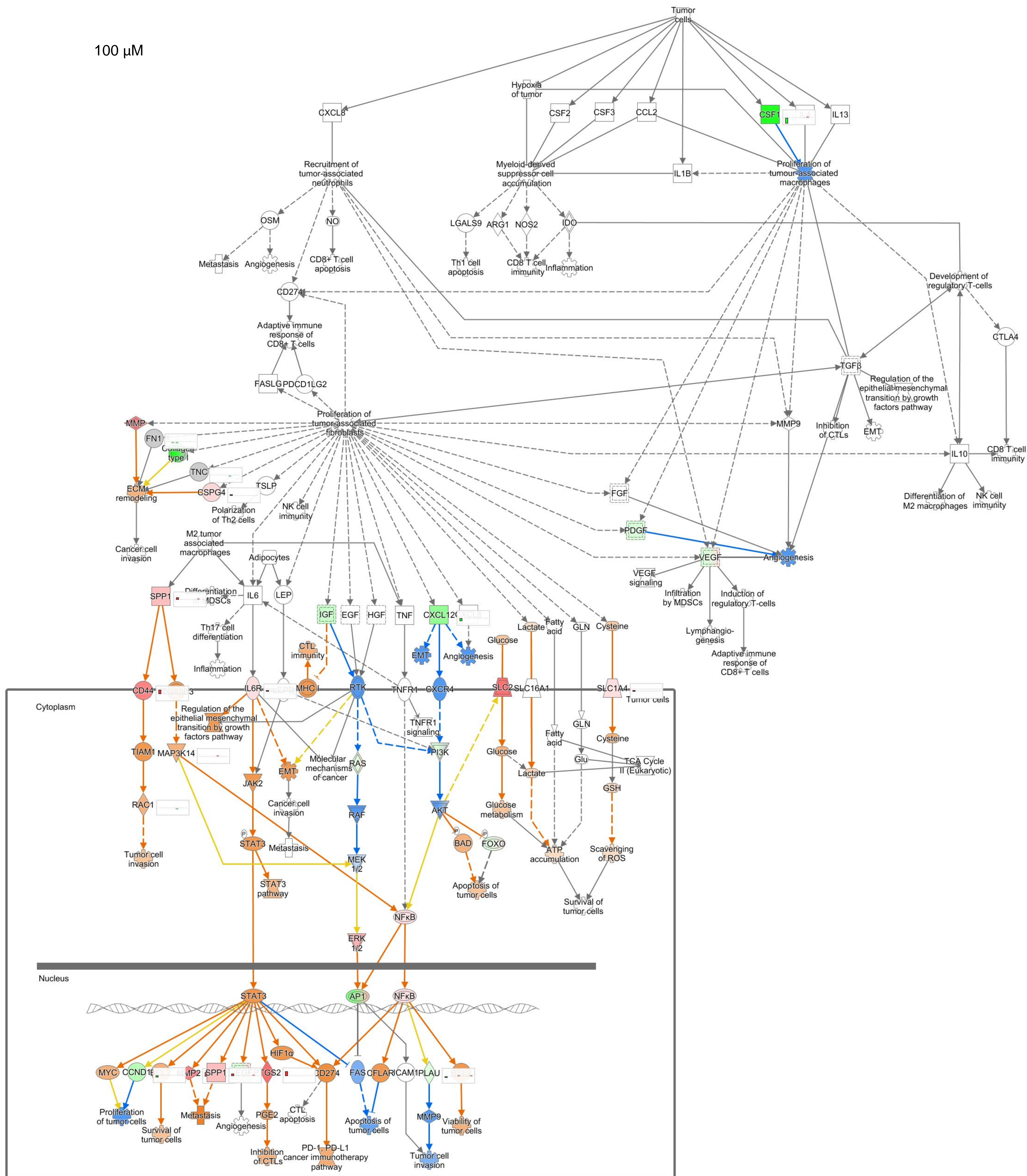

1  $\mu$ M

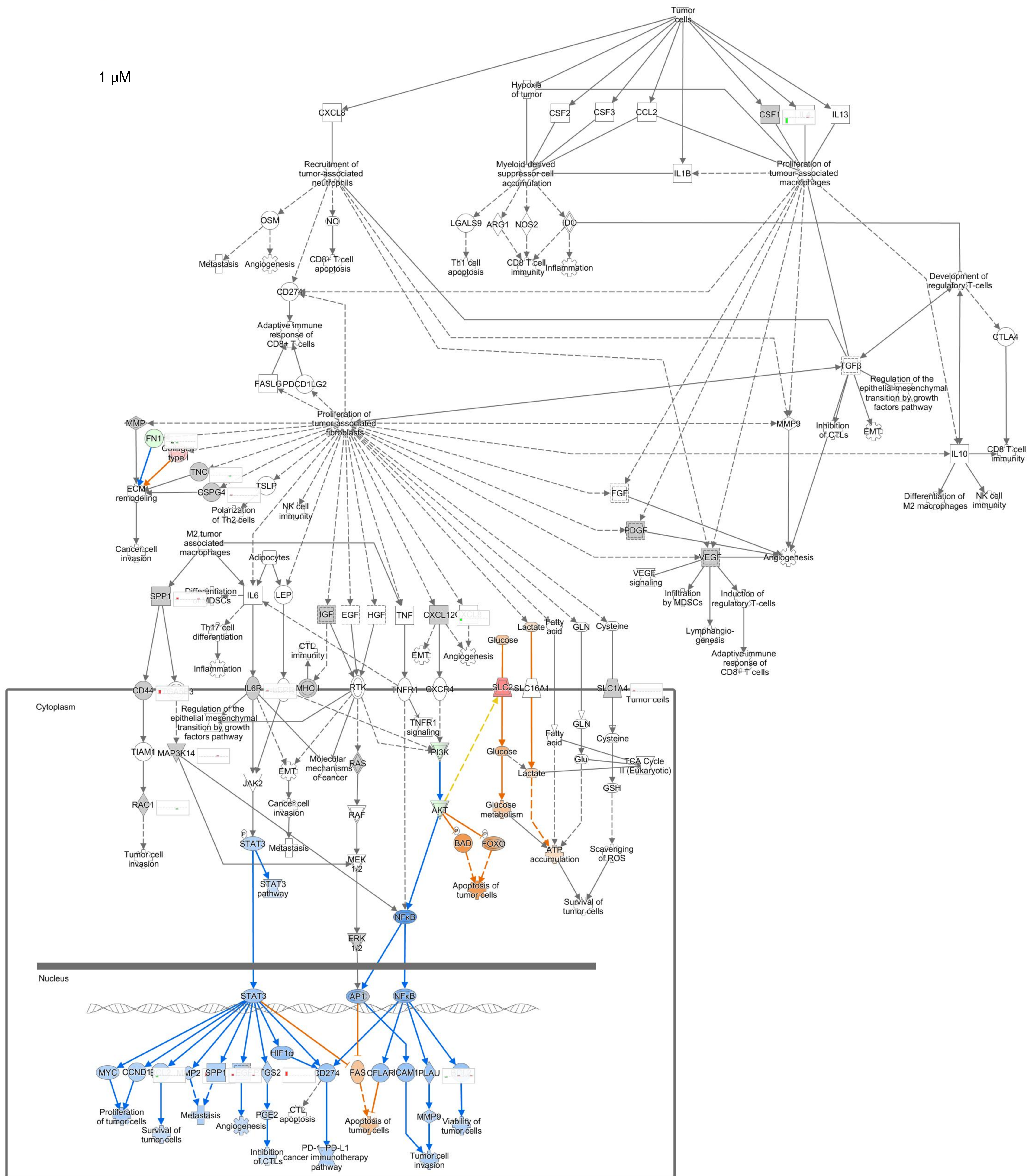

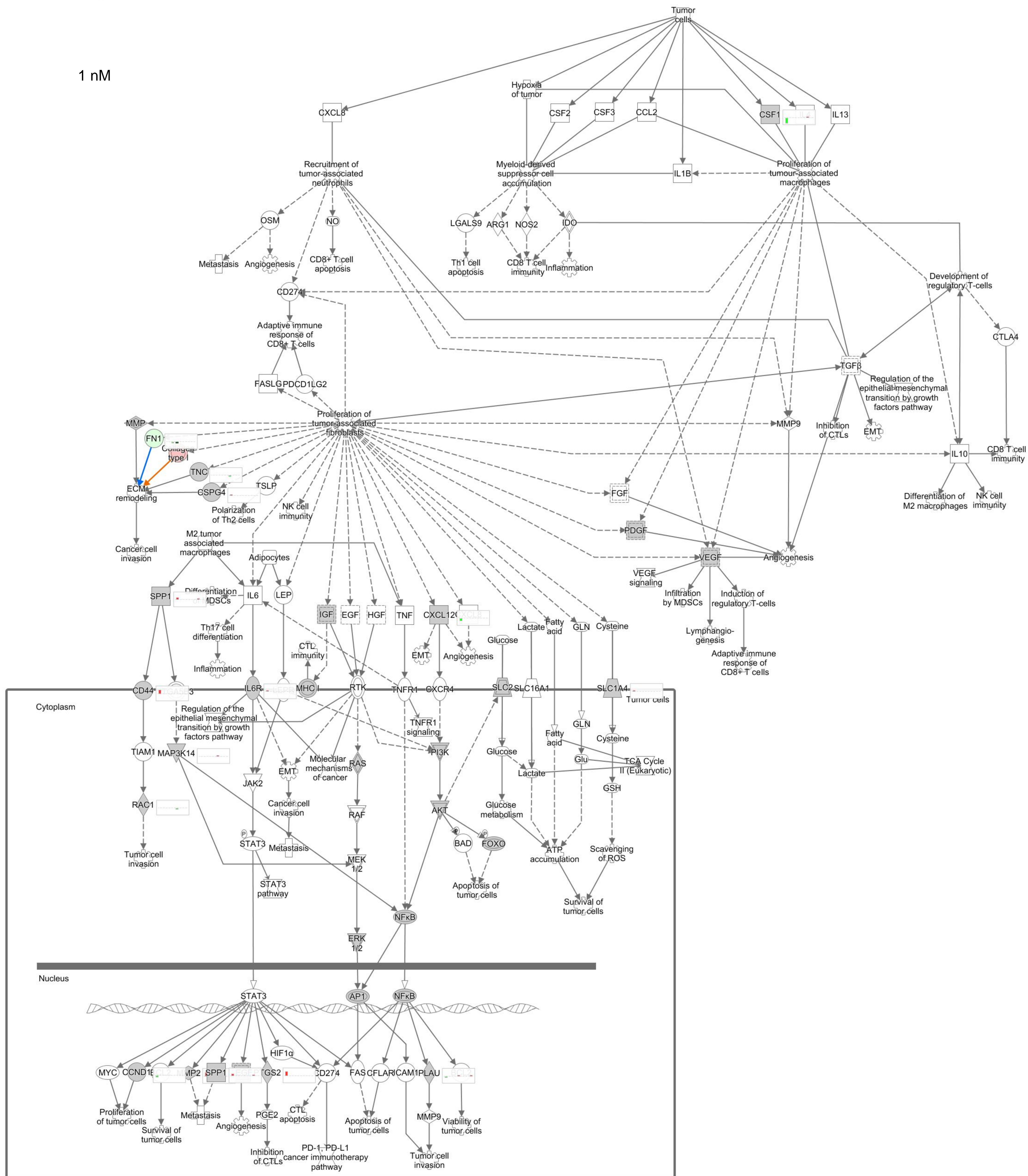

1 pM

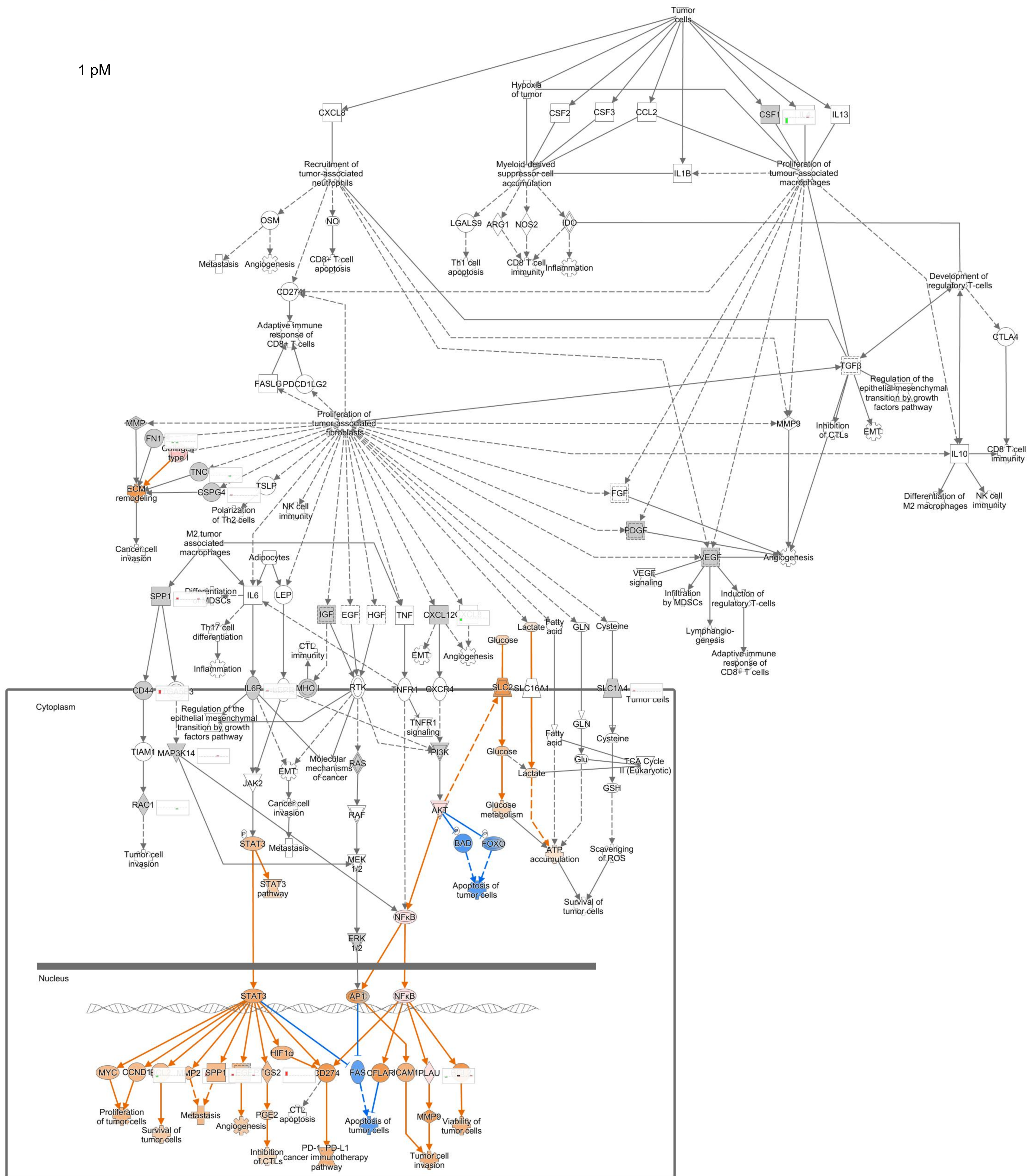

1 fM

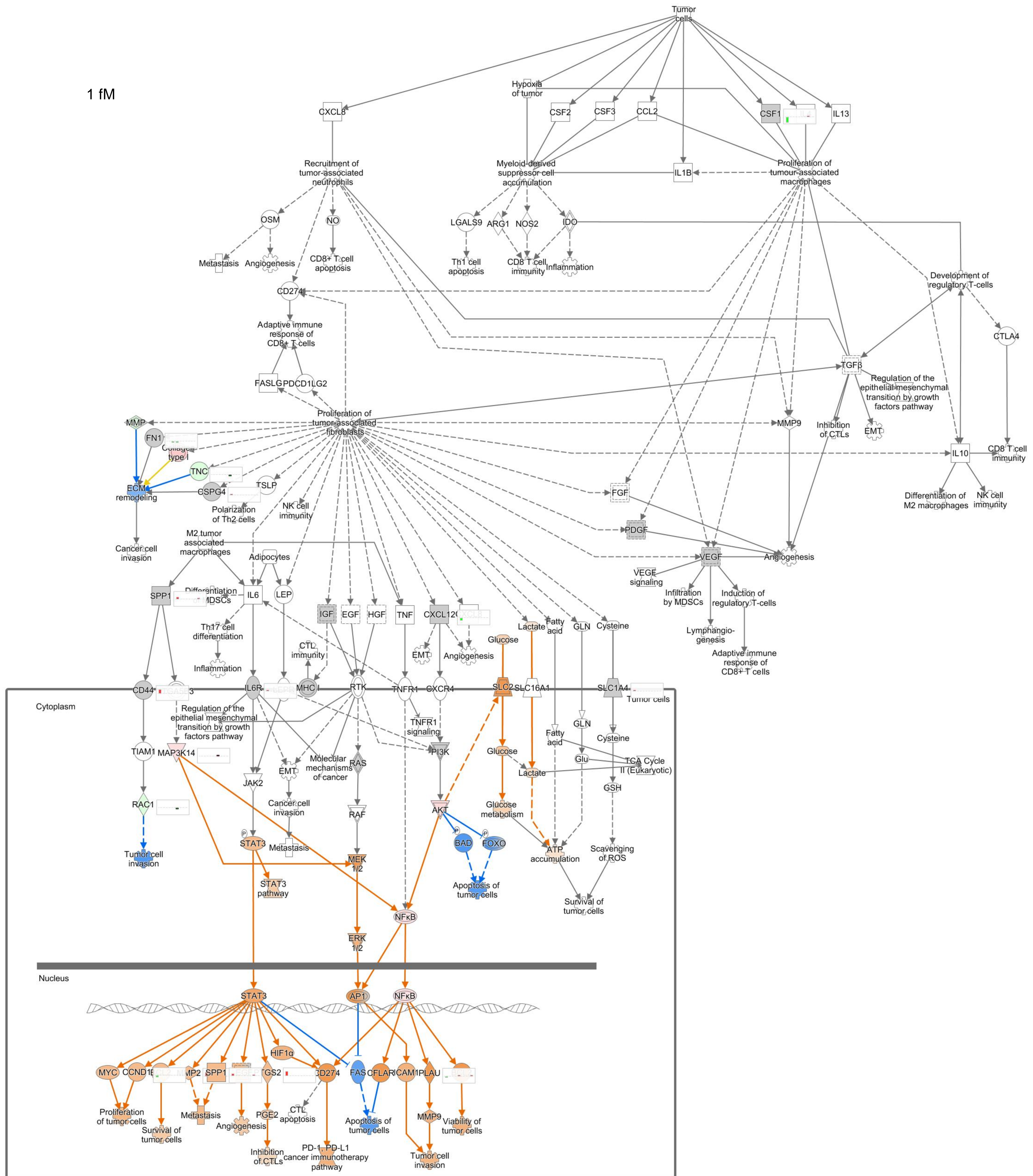

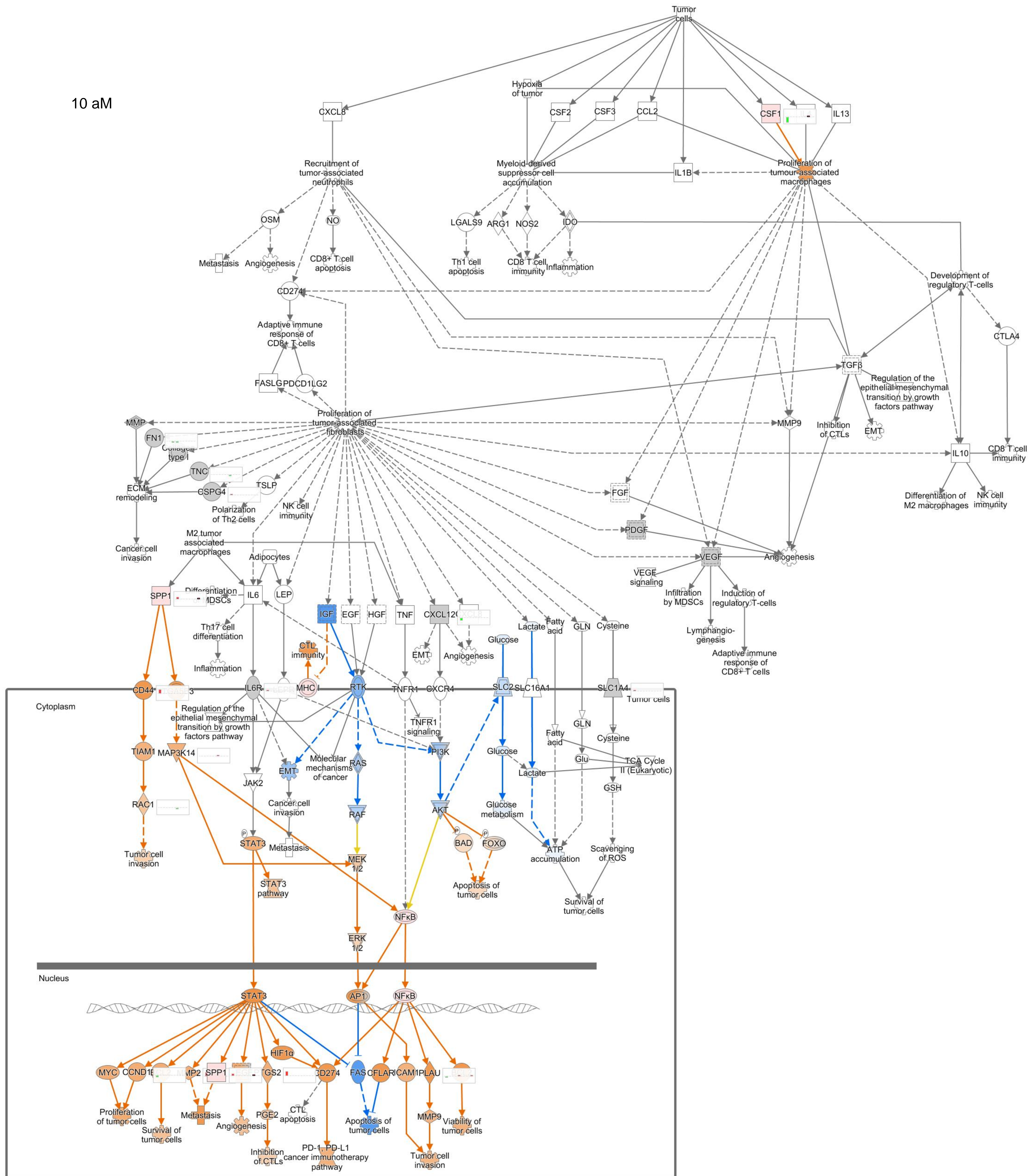

1 aM

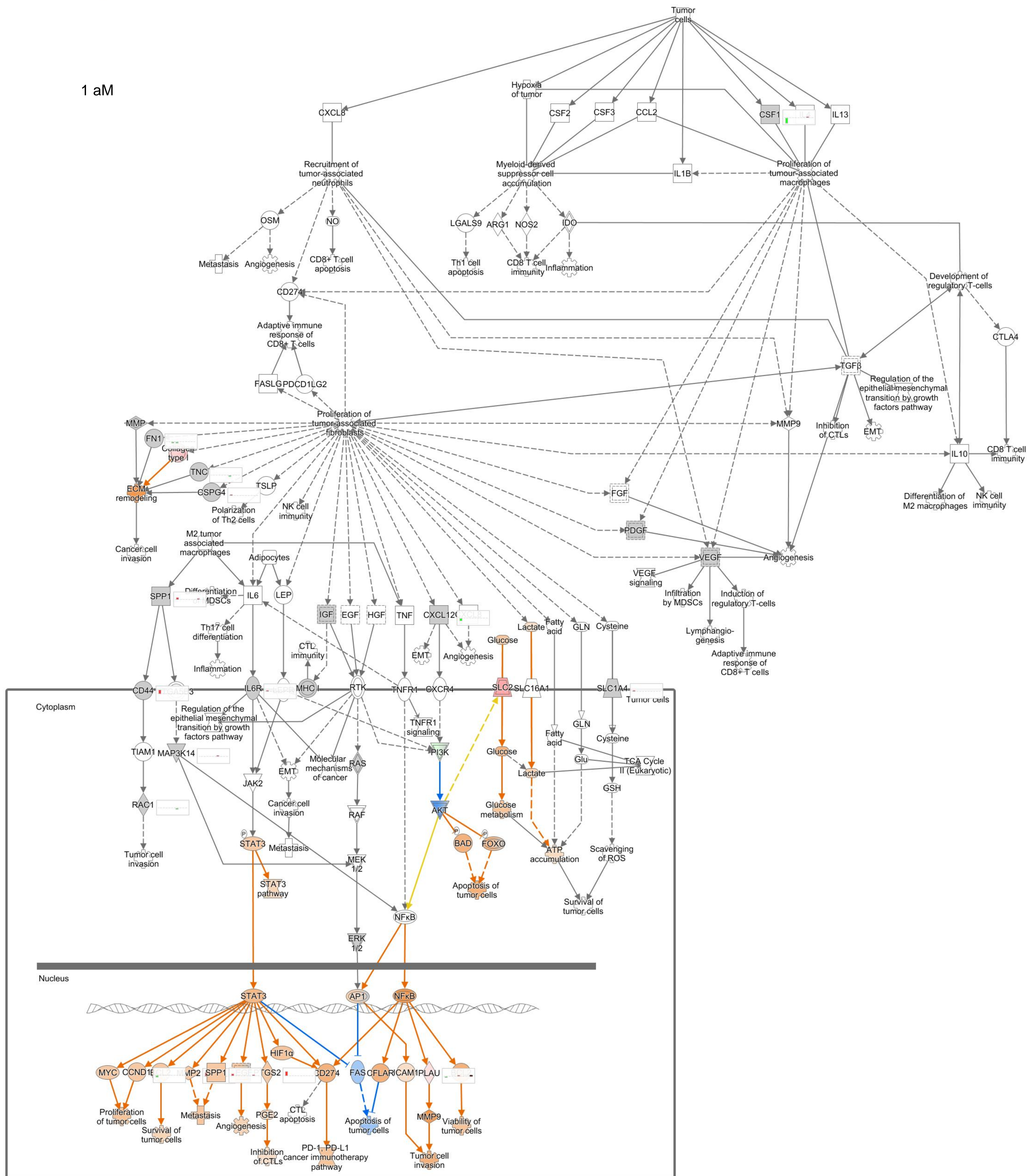

Supplement: Supplementary file 1 [file pharmaceuticals-14-00999-s001.zip › Suppl 10 Tumor microinvirovment pathway.pdf]
